# Ecological conditions experienced by offspring during pregnancy and early post-natal life determine mandible size in roe deer

**DOI:** 10.1371/journal.pone.0222150

**Published:** 2019-09-11

**Authors:** Anna Maria De Marinis, Roberta Chirichella, Elisa Bottero, Marco Apollonio

**Affiliations:** 1 Italian Institute for Environmental Protection and Research (ISPRA), Ozzano dell'Emilia (BO), Italy; 2 Department of Veterinary Medicine, University of Sassari, Sassari, Italy; University of Alberta, CANADA

## Abstract

Population dynamics studies and harvesting strategies often take advantage of body size measurements. Selected elements of the skeletal system such as mandibles, are often used as retrospective indices to describe body size. The variation in mandibular measurements reflects the variation in the ecological context and hence the variation in animal performance. We investigated the length of the anterior and posterior sections of the mandible in relation to the conditions experienced by juveniles of 8–10 months of age during prenatal and early postnatal life and we evaluated these parameters as ecological indicators of juvenile condition as well as female reproductive condition in a roe deer population living in the southern part of the species range. We analyzed a sample of over 24,000 mandibles of roe deer shot in 22 hunting districts in the Arezzo province (Tuscany, Central Italy) from 2005 to 2015 per age class. Mandible total length in juveniles is equal to 90% of total length in adults. In this stage of life the growing of the mandible’s anterior section is already completed while that of the posterior section is still ongoing. Environmental conditions conveyed by forest productivity, agricultural land use, local population density and climate strongly affected the growth of the anterior and posterior sections of the mandibles. Conditions experienced both by pregnant females and offspring played an important role in shaping the length of the anterior section, while the size of the posterior section was found to be related to the conditions experienced by offspring. Temporal changes of the length of the anterior section are a particularly suitable index of growth constraints. Anterior section length in fact differs according to more or less advantageous conditions recorded not only in the year of birth, but also in the previous year. Similarly, the sexual size dimorphism of the anterior section of the roe deer mandible can be used to describe the quality of females above two years of age, as well as habitat value. Hence the anterior section length of the mandible and its sexual size dimorphism are indexes that can provide cues of population performance, because they capture the system’s complexities, while remain simple enough to be easily and routinely used in the majority of European countries where roe deer hunting period extends from early autumn to late spring.

## Introduction

In ungulates, body size is correlated with juvenile/adult survival (e.g. Toigo and Gaillard [1] on ungulates in general; Clutton-Brock et al. [2] on Soay sheep *Ovis aries*, Gaillard et al. [3–4] on roe deer *Capreolus capreolus*, Loison et al. [5] on red deer *Cervus elaphus*, Côté and Festa-Bianchet [6] on mountain goat *Oreamnos americanus*, Festa Bianchet et al. [7] on bighorn sheep *Ovis canadensis*) and reproductive success (e.g. Mc Elligott et al. 2001 on fallow deer *Dama dama* [8], Douhard et al. [9] and Flajšman et al. [10] on roe deer, Albon et al. [11] and Bertouille and De Crombrugghe [12] on red deer, Albon et al. [13] on red deer and moose *Alces alces*). Demographic performance, and hence contribution to population growth, depends on body condition [14]. Therefore, studies on population dynamics and harvesting strategies often take advantage of body size measurements (e.g. Gamelon et al. [15]). The assessment of the causes of variation in body size is thus essential for the management and conservation of sustainable ungulate populations. The role of environment and climate in the growth process has been recognized for a long time. Nevertheless, the interplay between body size and habitat still needs further investigations. Environmental and climatic conditions prevailing during early life are decisive in shaping the architecture of the body and can affect the development both directly and indirectly through maternal condition (e.g. Forchhammer et al. [16] on Soay sheep, Weladji and Holand [17] on reindeer *Rangifer tarandus*). In ungulates the number, size and growth of offspring are determined by the energy allocated by the mother [18]. Maternal allocation may differ between ungulate species in relation to different strategies of acquisition and allocation of resources [19]. Females may rely on stored resources to sustain the costs of reproduction (capital breeders), or may not accumulate body reserves and rather use the energy acquired during the reproductive period (income breeders; see Stephens et al. [20]). In capital breeders, female body weight fluctuates both seasonally and annually. In income breeders, instead, female body weight varies only weakly with season and year. Small ungulates like roe deer belong to the group of high expenditure species, and are closer to the income breeder strategy of energy utilization [21]. The roe deer, a highly valued game species, has one of the largest distribution ranges among wild ungulates and is widespread and abundant almost all over the European continent [22]. Across its pan-European distribution, the species faces a wide diversity of environmental and climatic conditions and the female characteristics and their reproductive performance depends on these conditions [10,23]. Habitat transformations and weather changes during the crucial period of gestation and the earliest life stage, have profound, permanent effects on offspring and can influence their performance [24]. To date, the environmental and climatic influence on pregnant female condition has been little studied in wild populations, and there is currently no consensus about the expected direction of variations in this condition as a function of environmental and climatic harshness [25].

In ungulates, selected elements of the skeletal system are often used as retrospective indices of body size [26]. The mandible is one of the first bones in the body to ossify [27]. As Høye and Forchhammer [28] reported for roe deer, the medio-anterior section of the mandible reaches 95% of asymptotic size already at 2–4 months *post partum*, whereas the posterior section reaches 95% of asymptotic size at 14–16 months *post partum*. Hence, the size of the medio-anterior section of the mandible depends exclusively on resource availability *in utero* and during the first months *post partum*, while the size of the posterior section of the mandible reflects post-natal condition. The ultimate mandible size, the pattern of mandibular growth and the rate of mandibular development also change in relation to the population (e.g., Dvořák et al. [29] in Slovakia, Wustinger et al. [30] in Poland, Høye and Forchhammer [28] in Denmark, Zanneśe et al. [26] in France, Labus et al. [31] in Serbia, Avdić et al. [32] in Bosnia and Herzegovina, Hanzal et al. [33] in Czech Republic). Variability in quantity and quality of food resources determines the feeding habits of a population, which is manifested in morphological differences in mandible size [34–37]. Thus, analyses of mandibular measurements describe the relationship population/habitat quality [38–40] and provide clues to index population performance [41]. These complex interactions can cause differential responses of males and females to environmental/climatic factors which result in variations of the degree of sexual dimorphism across different ecological contexts [42]. For example, poor habitat quality results in decreased body sexual size dimorphism [43]; younger age classes of deer are usually more sensitive to environmental variation [44]

We studied a sample of over 24,000 mandibles of roe deer shot in 22 hunting districts in the Arezzo province (Tuscany, Central Italy). We analyzed the mandible total length per age classes for a population living in the southern part of the species range. We investigated factors (population density, environment and climate) experienced by the reproductive part of the female population (does above 2 years of age) during the gestation period and by the offspring (juveniles < 1 year old) during their first months of life. We tested if the variation of these factors per year influenced mandible size. About sexual dimorphism we assessed differences between sexes in an intermediate key stage of development for a rapid growth species such as the age class 8–10 months, whereas most published data have concerned neonates or adults [45–46].

The ecological indicators are used to monitor the relationship between the ecological context and the population condition and are based on body development [47]. We evaluated mandible measurements at juvenile stage as ecological indicators of the body condition of both juveniles and adult females.

## Materials and methods

### Study area

In Tuscany (Central Italy), roe deer hunting is only allowed in hunting districts: in the Arezzo province (3,235 km^2^; 43° 28′ N, 11° 53′ E), there are 22 hunting districts of about 95 km^2^ each, subdivided in 1,910 hunting grounds of 1.09 km^2^ ± 0.53 S.D. (S1 Fig). The hunting districts are evenly distributed throughout the province, covering the 64.54% of the whole area. The northern part of the Arezzo province is mostly mountainous, including the Apennine chain and other secondary chains; 66% of this area is forested, with oaks (*Quercus cerris* and *Q*. *pubescens*) being the dominant species along with beech (*Fagus sylvatica*) and sweet chestnut (*Castanea sativa*), while conifers only amount to 6.5%. The southern part includes the lower course of the Arno River and Chiana Valley, the Chianti Hills and some low mountains; approximately 50% of this area consists of cultivated fields and only 32% is wooded.

The climate is temperate-continental, with a mean temperature ranging from 1.4°C in January to 24.9°C in July. The study area harbors a rich wild ungulate community: besides roe deer, which is present in 80% of the province, there are wild boar (*Sus scrofa*), fallow deer (*Dama dama*), red deer and mouflon (*Ovis aries musimon*). Wild boar is homogeneously distributed across the whole province, whereas red deer, fallow deer and mouflon are more localized [48]. Wolf (*Canis lupus*), with an estimated number of 25 packs [49], and red fox (*Vulpes vulpes*) are also present.

### Data collection

A total of 24,972 roe deer (12,026 females and 12,946 males) were legally shot during the annual harvest (August 1^st^-September 30^th^ and January 1^st^-March 15^th^) from 2005 to 2015 in 22 hunting districts of Arezzo province. Each mandible was registered by the Provincial Government and made available to us for measurements. The hunters had hunting permits and culling times/methods/hunting bags were regulated by the Regional Laws n. 3 of 12/1/1994 (and subsequent modifications/integrations), n. 20 of 10/6/2002 (and subsequent modifications/integrations) and n. 10 of 9/2/2016. The animals were not killed for research purposes. Thus, research activities did not require any specific authorization to be conducted.

The sample was divided into 4 age classes (see S1 Table for major details about the criteria used to assess the 4 age classes and S2 Table for the sample size), according to teeth eruption and exposed dentin on M_3_ [23,50]. Date of culling, sex, body weight and hunting zone of each roe deer were recorded. Body weight is dressed weight (i.e. live weight minus viscera and bleedable blood).

#### Mandible variables (Fig 1)

Total length was measured with a digital caliper to the nearest 0.01 mm. The length of the anterior and posterior sections was measured only on a subsample of 2,161 mandibles of juveniles (8–10 months; data reported in S3 Table), collected during the following hunting periods: 2013 (225 females and 171 males), 2014 (477 females and 410 males) and 2015 (474 females and 404 males). Age class was identified based on teeth eruption stages.

**Fig 1:**
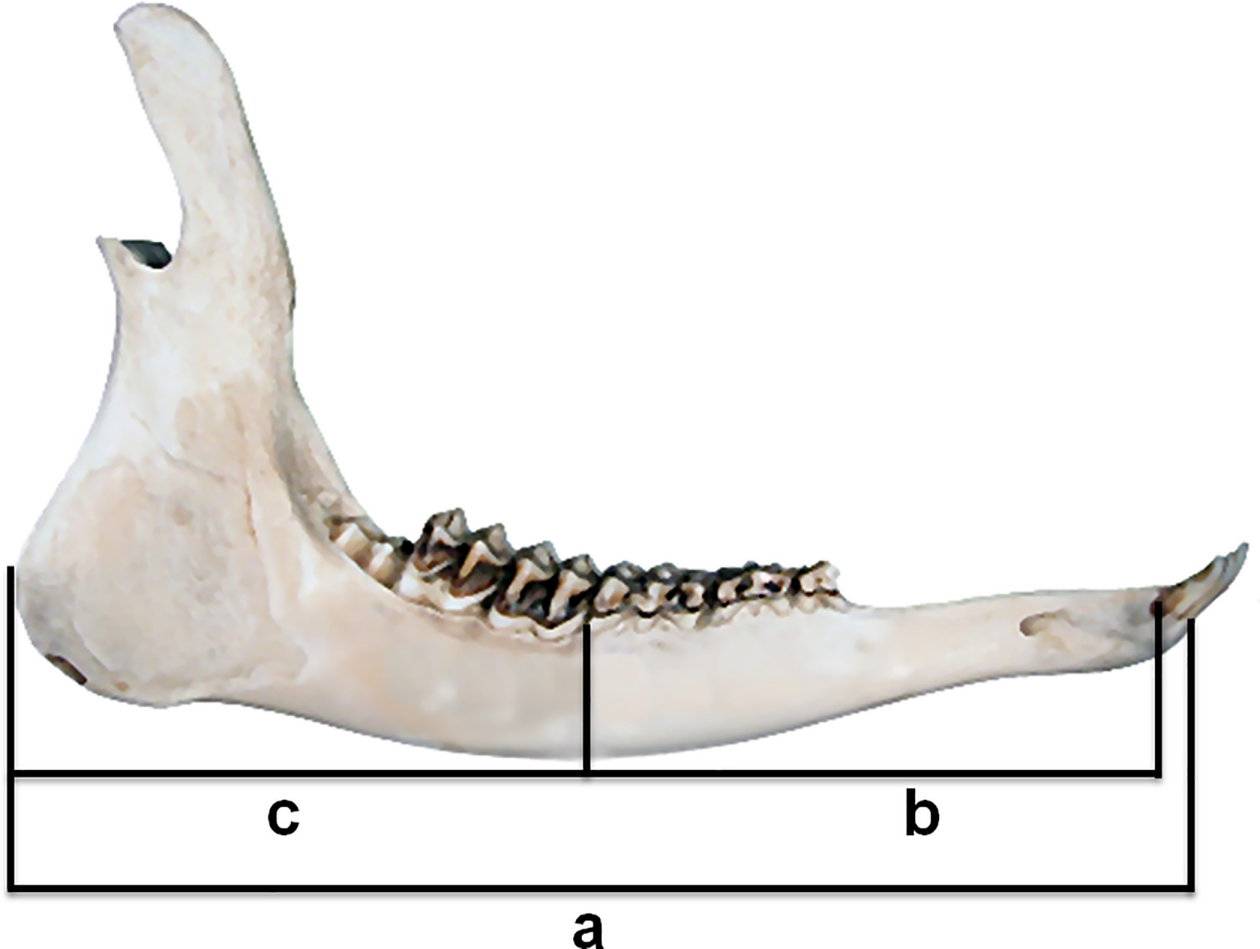
Measurements on roe deer mandible. a: total length measured from the anterior margin of the alveolus of I_1_ to the posterior margin of the *processus angularis*; b: length of the anterior section measured from the posterior margin of the alveolus of C to the posterior margin of the alveolus of P_4_; c: length of the posterior section measured from the posterior margin of the alveolus of P_4_ to the posterior margin of the *processus angularis*.

The hunting period 2013–2015 was characterized by peculiar climatic conditions. The annual average temperature recorded in 2015 was the highest since 1961, with an average temperature increase equal to +1.70°C in central Italy (maximum average value in summer +4.27°C) and annual precipitation was 17% lower than the climatological averages of the area [51]. Indeed, year 2015 is in third place in the ranking of the driest years since 1961 [51]. Such exceptional climatic conditions are suitable to study how weather and environment can affect the mandible size in a fast developing species.

#### Environmental and climatic variables (S4 Table)

To the purpose of our analysis, the ecological conditions experienced by reproductive females were defined by: i) mean seasonal temperature and total precipitation in the initial (December-February) and final (March-May) period of pregnancy; ii) winter harshness and duration (December-March) during pregnancy, assessed with MODIS snow cover data; iii) forest productivity in the pre-rut period (March-mid July), in the rut period (mid July-August), and in the early diapause (September-October), assessed with MODIS fPAR data [52].

The ecological conditions experienced by offspring were defined by: i) mean seasonal temperature and total precipitation in the first summer (June-mid September) and first autumn-early winter (mid September-December); ii) winter harshness and duration during the first winter of life, assessed as reported above; iii) forest productivity in the early life until the beginning of autumn, assessed as reported above. The birth period was defined as the period between the first and the last record of females with newborn [53]; this period was estimated to last about 30 days, with a peak in mid May (data available from standardised field observations). These environmental and climatic variables were measured in relation to the hunting grounds together with the percentage of agricultural land.

#### Population density (S4 Table)

Roe deer densities were obtained by drive censuses [54–57]. Data were collected in May and June on a network of 187 permanent sample areas (0.44 km^2^ ± 0.26 S.D. on a total area of 81.16 km^2^; see S1 Fig for the locations of the network of sample areas and S5 Table for their number, extent and percentage in each hunting district) homogenously distributed throughout the study area and representative of the landscape structure of the hunting grounds in each district. On average the 4% of the surface area in each district was sampled (see S5 Table). In support of this methodological approach, [58] considered the reliability of this density estimation method and suggested that at higher roe deer densities (more than 5–7 heads/100 ha as in our study area), especially in period with low aggregation level of the species as during our monitoring period (i.e., May-June), drive counts can provide reliable information on population size. However, the proportion of area counted in this study (4%) was lower than that tested in Borkowski et al. [58]’s simulations (10%). Roe deer density at a local scale was calculated by spatial interpolation using the inverse distance weighting method [59] in ArcGis 10.1 (ESRI Inc., Redlands, CA, USA).

Since the growth of the deer mandible is a sensitive process and its length may be considered a sensitive indicator of life time nutritional *status* [60], we reported in Table 1 the relative predicted effects of all the explanatory variables potentially able to affect the mandible size *in utero* and *post partum*.

**Table 1:** Predicted effects on the growth of the anterior and posterior sections of mandible.

	Variables	Predicted effect	
		Anterior section	Posterior section
**Individual**	Offspring body weight	+	+
	Adult female body weight	+	
	Julian date	+	+
**Weather**	*Temperature*		
	Autumn _f_	+	
	Winter _f_	+	
	Spring _f_	+	
	Summer _f+o_	-	-
	Autumn _o_		-
	*Precipitation*		
	Autumn _f_	-	
	Winter _f_	-	
	Spring _f_	+	
	Summer _f+o_	+	+
	Autumn _o_		-
	*Snow cover*_f_	-	
**Environment**	Forest productivity _f+o_	+	+
	Agricultural land _f+o_	+	+
**Population**	Density _f+o_	-	-

Expected effects of the explanatory variables recorded in each hunting zone of the Arezzo province (Tuscany, Central Italy) on the growth of the anterior and posterior sections of mandible. Further details on the explanatory variables are reported in S3 Table.

_f_: related to the adult females; _o_: related to the offspring; _f+o_: related to the adult females and offspring.

### Statistical analysis

We derived the total mandible length through the 4 age classes and for each class we calculated the percentage on total length achieved in the 4^th^ age class (i.e., ≥27 months).

The sexual size dimorphism (SSD) of the anterior and posterior section of the mandible was evaluated through the following ratio: $SSD=\frac{average\;section\;length\;♂-average\;section\;length\;♀}{average\;section\;length\;♂}$. Differences in the length of anterior and posterior section per sex and year were tested throughout *t* test and ANOVA test.

General Linear Models (GLM, family = gaussian) were fitted with R software version 3.4.4 [61] to examine variation in the growing rates of the anterior and posterior sections. Models were fitted with all biologically meaningful combinations of predictors reported in Table 1 and described in S4 Table. We added the Julian date as independent variable in order to eliminate the effect of the culling date. To avoid problems of multicollinearity, highly correlated predictors (Pearson correlation coefficient r_p_ ≥ 0.7) were not selected for the same model. Although the number of environmental factors we analyzed was high, only two pairwise correlations were higher than 0.7 (S6 Table), the threshold usually retained for collinearity [62]. In such cases, we only retained one of the highly correlated variables. We used the Information-Theoretic (IT) approach based on Akaike information criterion (AIC; [63–64]) to select the best fitting models. All models were averaged under delta AIC = 2, weighed by AIC weights.

## Results

### Mandible size

**Fig 2:**
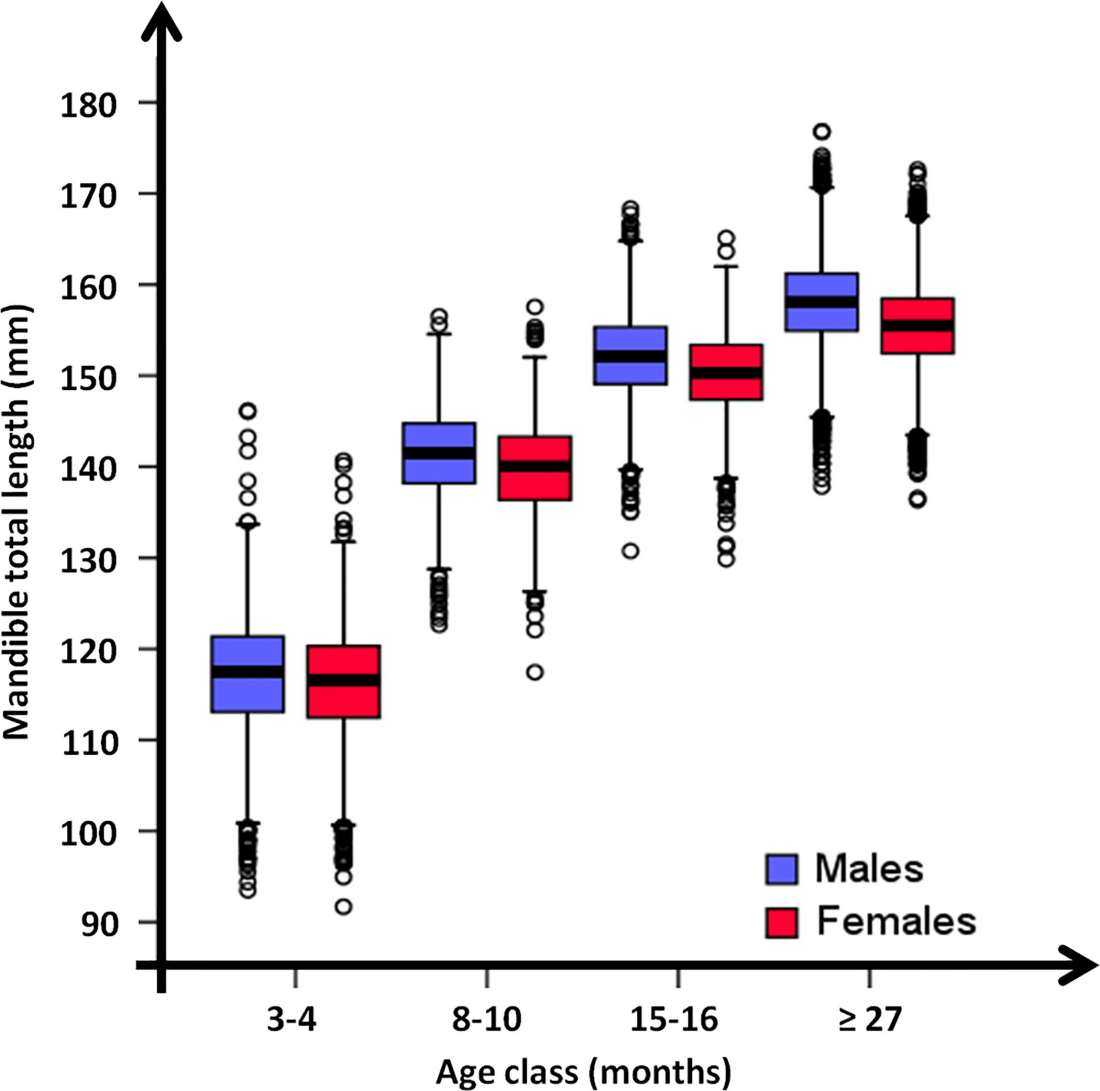
Mandible growth. Mandible total length in relation to the age class of 24,972 roe deer (12,026 females and 12,946 males) legally shot during the annual harvest (August 1^st^-September 30^th^ and January 1^st^-March 15^th^) from 2005 to 2015 in Arezzo province (Tuscany, Central Italy).

**Fig 3:**
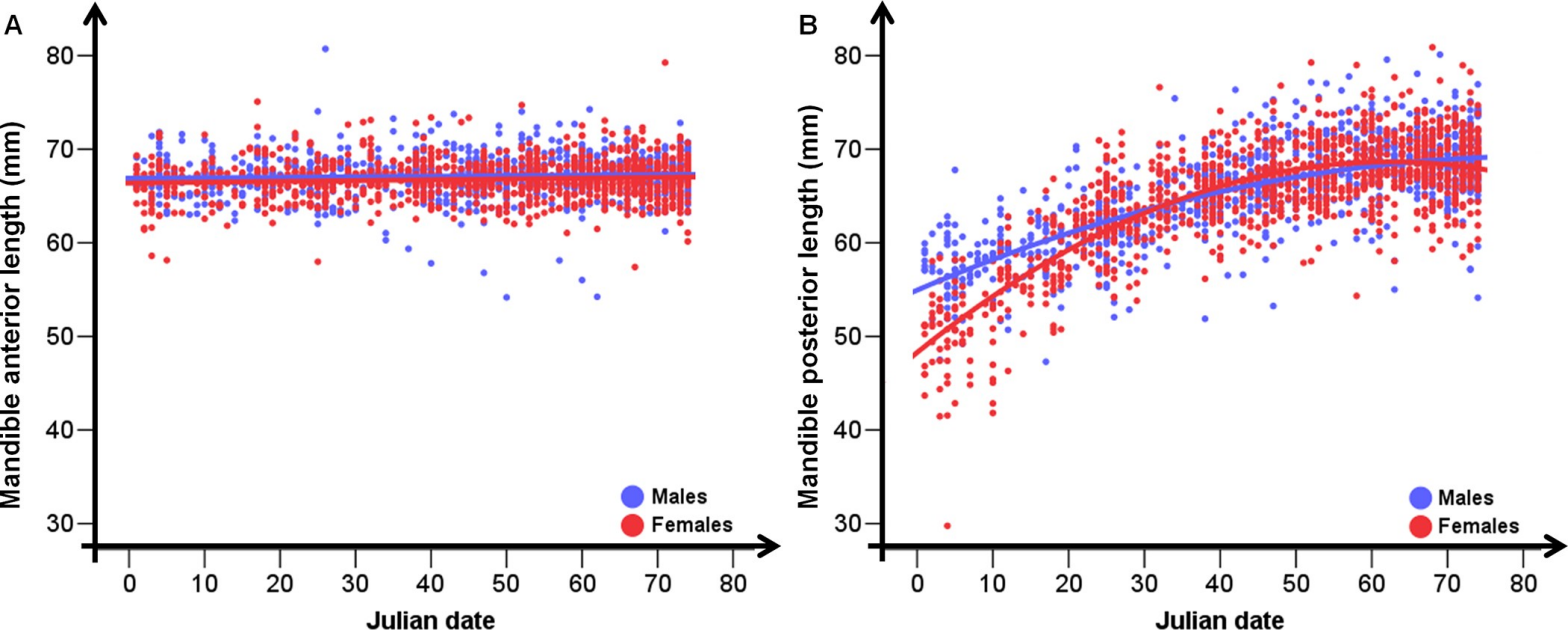
Mandible sections growth. Length of the anterior (A) and posterior (B) section of 2,161 mandibles of juveniles (8–10 months; 1,176 females and 985 males), collected during the 2013–2015 hunting seasons in the Arezzo province (Tuscany, Central Italy), in relation to the Julian date (1^st^ January-15^th^ March).

Average total length was 155.44 mm in adult females and 158.04 mm in adult males (Fig 2 and S2 Table). In the first winter of life (age class 8–10 months), the mandible length was equal to 90% of the total length reported for the age class ≥ 27 months. The growing of the anterior section of the mandible was already completed; average length was 66.69 mm in females and 67.14 mm in males (t = 3.69, *p*<0.01; Fig 3). The growing of the posterior section was not completed; the measurement was different between sexes only in January (t = 4.09, *p*<0.01; Fig 3); in this month females’ growth rate was higher than males’.

**Fig 4:**
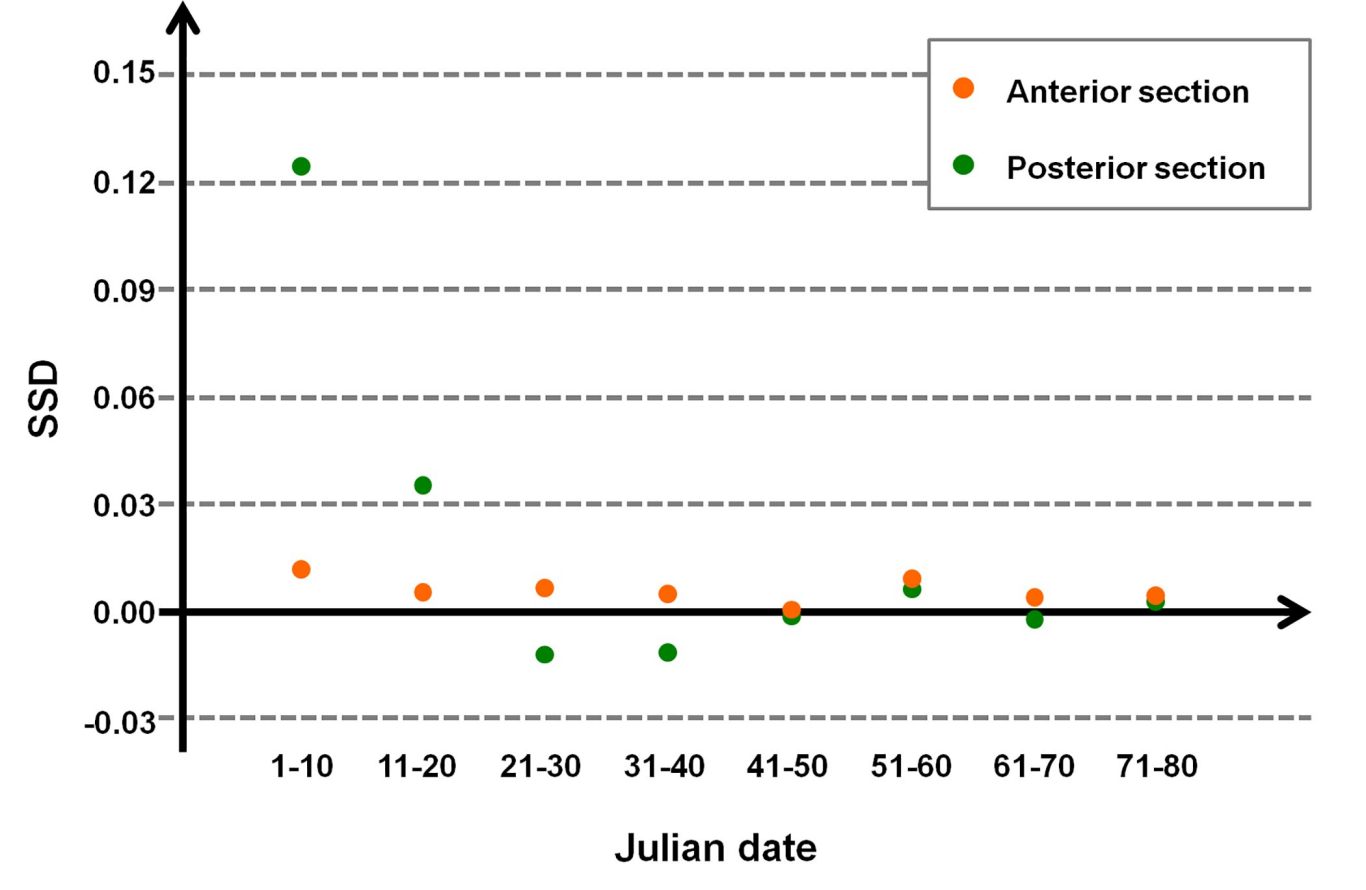
Sexual size dimorphism (SSD). SSD in the length of the anterior and posterior section of 2,161 mandibles of juveniles (8–10 months; 1,176 females and 985 males), collected during the 2013–2015 hunting seasons in the Arezzo province (Tuscany, Central Italy), in relation to the Julian date (1^st^ January-15^th^ March).

The size and variation of sexual dimorphism on the anterior and posterior sections of the mandible were reported in Fig 4. The anterior section showed a very low sexual size dimorphism (average SSD = 0.006) that remained practically constant during the first winter of life. The posterior section showed a higher value of sexual size dimorphism (SSD = 0.12) at the beginning of January; this value decreased during this month until it reached that of the anterior section.

$$\mathrm{SSD}=\frac{section\;average\;length\;♂-section\;average\;length\;♀}{section\;average\;length\;♂}$$

### Factors affecting mandible size

**Fig 5:**
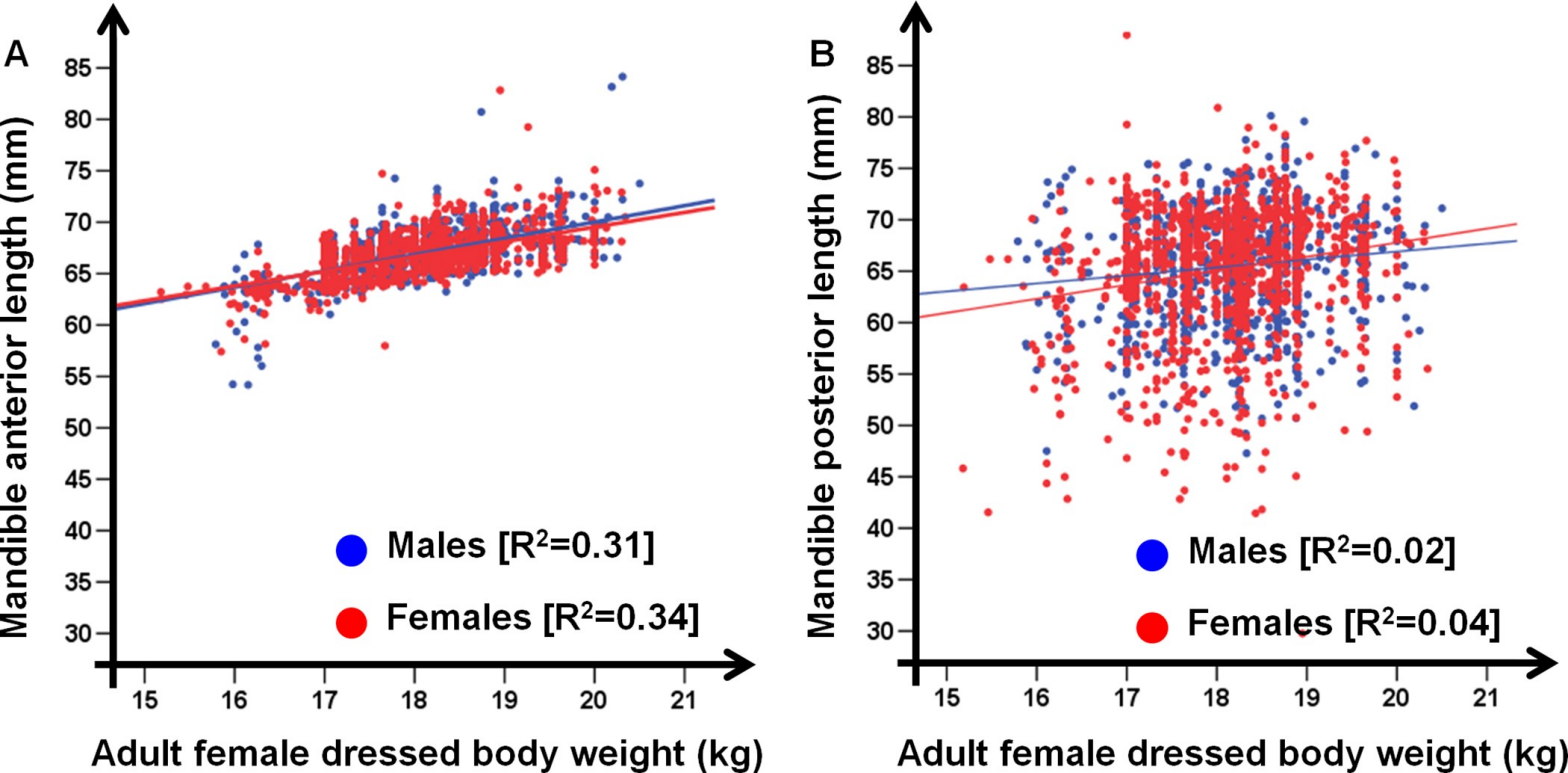
Mandible sections length. Length of the anterior (A) and posterior (B) section of 2,161 mandibles of juveniles (8–10 months; 1,176 females and 985 males), collected during the 2013–2015 hunting seasons (1^st^ January-15^th^ March) in the Arezzo province (Tuscany, Central Italy), in relation to the dressed body weight of adult females (≥ 2 years). R^2^ is reported for each regression line.

The final models describing length variation in the anterior and posterior sections of the mandible were reported in Table 2 (see S7 Table for major details about model selection procedure). Section development was influenced by sex, with males having bigger mandibles than females. Winter high temperatures, spring rainfall, forest productivity and agricultural land use showed a positive effect in determining the final length of the anterior section, while a negative effect was associated to higher summer temperature and higher local population density. Offspring and reproductive females’ dressed body weight played an important role in this process (Fig 5) with positive effects on the anterior section size. The growth of the posterior section is more related to the conditions experienced by the offspring, with a positive effect of forest productivity and juvenile dressed body weight and a negative effect of summer-autumn high temperature and autumn rainfall. Agricultural land use and higher local population density affected also the size of the posterior section respectively in a positive and negative way. The statistically significant effect of the Julian date confirmed that the growth process is still under way in roe deer at 8–10 months of age (Fig 5).

**Table 2:** Averaged models.

	Variables	Anterior section				Posterior section			
		Estimate	SE	t value	*P*	Estimate	SE	t value	*p*
**Intercept**		59.18	3.13	37.49	<0.01	49.17	2.58	18.15	<0.01
**Individual**	Offspring body weight	2.21	0.32	7.58	<0.01	3.11	0.42	12.34	<0.01
	Adult female body weight	3.65	0.50	11.81	<0.01				
	Sex	0.20	0.08	2.14	0.02	0.89	0.16	2.85	<0.01
	Julian date	0.18	0.06	1.71	0.06	1.94	0.24	5.97	<0.01
**Weather**	*Temperature*								
	Autumn _f_								
	Winter _f_	0.24	0.08	2.19	0.02				
	Spring _f_								
	Summer _f+o_	-1.43	0.29	-3.96	<0.01	-2.93	0.53	-6.74	<0.01
	Autumn _o_					-0.28	0.16	-0.39	0.07
	*Precipitation*								
	Autumn _f_								
	Winter _f_								
	Spring _f_	0.19	0.10	0.78	0.08				
	Summer _f+o_								
	Autumn _o_					-0.39	0.16	-0.71	0.04
	*Snow cover*_f_								
**Environment**	Forest productivity _f+o_	2.58	0.49	8.72	<0.01	1.51	0.34	3.14	<0.01
	Agricultural land _f+o_	0.99	0.11	3.14	<0.01	1.12	0.29	2.85	<0.01
**Population**	Density _f+o_	-1.24	0.36	-4.51	<0.01	-0.89	0.37	-0.77	0.03
**Combined effects**	Sex × Summer Temperature _f+o_	-	-	-	-	-1.43	0.29	3.01	<0.01
	Sex × Agricultural land _f+o_	-	-	-	-	0.21	0.11	0.49	0.010

Parameter estimates, standard errors, t value and *p* value of the best models (ΔAIC ≤ 2) explaining the variation in the length of the anterior and posterior sections of the mandible for juvenile roe deer (8–10 months) hunted within the Arezzo Province from January to March over three consecutive hunting seasons (2013–2015). The averaged model was weighed through AIC weights (anterior section: R^2^ = 0.42; posterior section: R^2^ = 0.38). Further details on the explanatory variables and model selection procedure are reported respectively in S4 and S7 Tables.

**Fig 6:**
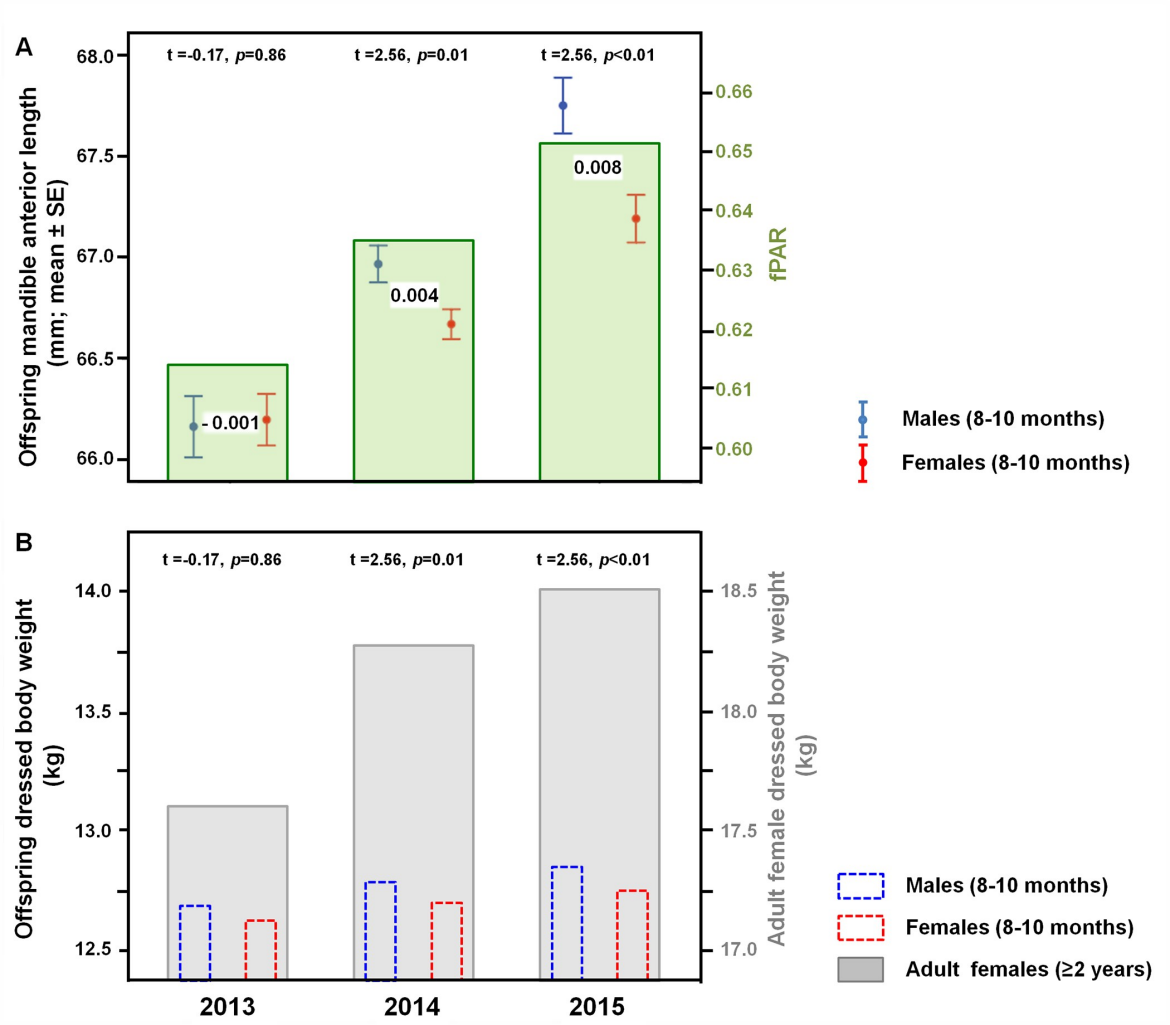
Mandible anterior section as an index of growth constraints. A: Length of the anterior section of 2,161 mandibles of juveniles (8–10 months; 1,176 females and 985 males) in relation to the forest productivity (fPAR) in March-October during the 2013–2015 hunting seasons (1^st^ January-15^th^ March) in the Arezzo province (Tuscany, Central Italy). SSD is reported for each year between error bars and differences between sexes were reported (t test). B: Dressed body weight of 2,161 juveniles (8–10 months; 1,176 females and 985 males) in relation to the dressed body weight of adult females (≥ 2 years) during the 2013–2015 hunting seasons (1^st^ January-15^th^ March) in the Arezzo province (Tuscany, Central Italy). Differences between sexes were reported (t test).

On the contrary, the growing of the anterior section was completed before the first winter of life, hence no compensation growth could occur in the successive months. Differences in length of the anterior section per year reflected differences in dressed body weight of juveniles (F = 31.84; *p*<0.01; Fig 6). A greater forest productivity (fPAR) in March-October and a bigger dressed body weight of reproductive females induced an increase in mandible size and body mass of fawns, especially males. The sexual size dimorphism changed per year and reached the highest value in 2015, when environmental and climatic conditions were very favorable (Fig 6).

## Discussion

### Mandible size

The growth of the anterior and posterior sections of the mandible is not assessable only by examining a sample of 8–10 month-old roe deer, because at this age the development of the first section is already completed, while that of the second section is still ongoing. The posterior section showed a sex-specific growth: it grew faster in females than in males. This different growth rate is likely due to the trade-off between growth and energy allocation to reproduction, which occurs earlier in females than in males [43]. The timing of the first reproductive event constitutes, in fact, a major physiological and energetic constraint that shapes sex-specific patterns in skeletal development [9]. Hence, conditions in early life mostly affect body growth of females and can induce long-lasting effects on their performance. The longer duration of body growth in males confers on them a greater flexibility so that they can select a capital breeder tactic, that allows the accumulation of reserves to offset reproductive costs [43]. Further researches are needed to determine the relationships between the possible compensative growth mechanisms and environmental/climatic factors for females.

### Factors affecting mandible size

Habitat quality expressed through forest productivity, together with environmental conditions, local population density and climate decidedly affected the growth of the anterior and posterior sections of the mandible.

Roe deer is a highly selective feeder [65–66] that can also exhibit opportunistic behavior in relation to food resource availability in a given place, season and/or year [67]. Forest productivity—used here as a proxy of food availability [41] during the last part of gestation, the birth period, and the lactation—sped up mandible growth. Roe deer females do not accumulate body reserves [21] and are thus expected to respond immediately to changes in food resource availability during winter, spring and summer when reproductive energetic costs peak [68], by adjusting the amount of energy allocated to the offspring. Since roe deer fawns remain associated with their mother throughout their first year of life, their growing depends also on the richness of the maternal home range [69]. In fact, this species reaches about 70% of the adult body mass [43] and about 90% of the ultimate total mandible length within the first 8–10 months of life. Spatial heterogeneities in home range habitat quality can induce spatial variability in fawn condition, suggesting that spatial variation could be as important as temporal variation in shaping individual variability [70]. Forest productivity, in turn, obviously depends on environmental conditions and climate (e.g., Bisi et al. [71]). Moreover, longer mandibles were collected in hunting districts with a greater percentage of agricultural land, an important habitat for a species that exhibits behavioural plasticity [72].

Mandible length was also sensitive to changes in population density as shown in other ungulate species (e.g., Azorit et al. [73] and Couturier et al. [74]). However, a density dependent effect should be more pronounced in roe deer because the growth is more rapid, as reported by Zanneśe et al. [26] for hind foot length at a landscape scale.

As for climate conditions, the winter temperature, the spring precipitation experienced by the female during gestation and the subsequent summer temperature experienced during lactation period positively affected the growth of the anterior section of the mandible. Conversely, high temperatures during the first summer and high precipitation during the first autumn experienced by the offspring delayed the growth of the posterior section of the mandible.

Warmer winters, hotter summers and the variation in the amount/pattern of rainfall due to global climate changes [51] seem to have conflicting effects on the pregnant female condition and the growing fawn. Recent studies revealed a mismatch between birth date and vegetation phenology that can decrease the early survival rate in juveniles [75]. Since roe deer birth date is driven by day length rather than resource availability, a variation in birth timing should not occur [75]. Therefore, given the forecast of future climatic conditions and considering the development of offspring as a critical stage in roe deer population dynamics [76], global climate change is expected to affect both individual and population level.

In years characterized by favorable environmental/climatic conditions, such as 2015, the anterior section of the mandible was more developed and the sexual size dimorphism was higher. Fawn condition depends on pregnant female condition and well-fed, heavier females produce larger embryos than lighter or primiparous females. Embryonic development differs between male and female embryos, the latter being smaller and weighing less than males [77]. Therefore, favorable environmental and climatic conditions result in increased body sexual size dimorphism.

## Conclusions

Given the increase of roe deer populations in Europe, assessing the relationship between a population and its habitat shall be necessary in order to make appropriate management decisions on a quantitative basis [78]. Ecological indicators can provide a measurable assessment of such relationships and its fluctuations in time and space. Monitoring changes of these indicators supplies a new basis for setting hunting quotas to preserve the population-habitat balance. To date, the length of the mandible has generally been used as index of quality and performance of individuals in a population. Mandibles development rate is less marked in stable environments; however, with varying levels of competition, under changing management practices and climatic changes, a fast maturing bone is pertinent as an index, because it restricts the variability in growth conditions responsible for the asymptotic size of the individual skeletal part.

Our study revealed that the length of the anterior section of the roe deer mandible is particularly suitable as an ecological indicator. This measurement indexes prenatal and early development, minimizing the time window where growth constraints can influence asymptotic size, directly and/or indirectly through the effects of female condition.

In accordance to Høye and Forchhammer [28], we suggest that the length of the anterior section of the mandible rather than the total length of the mandible is an index of growth constraints. The performance variability can be related to the health condition of the pregnant females, which, in turn, are related to the different home-range quality [69]. Differences in mandible size are influenced by favorable conditions recorded not only in the year of birth but also in the previous year. Such pervasive effects of environmental/climatic conditions are related to the very sedentary habits of adult roe deer [70]. Similarly, the sexual size dimorphism of the anterior section of the roe deer mandible can be used to describe reproductive female and habitat quality. Hence, these indexes provide cues of population performance.

Finally, a further reason to promote the use of this indicator is that the data collection involves only roe deer under 1 year of age, whose age class can be correctly determined and who are particularly sensitive to the variation in environmental and climatic conditions because of its requirements for rapid growth [79–80]. Data on the length of the anterior section of the mandible could be acquired from offspring hunted from the early autumn to the late spring, thus overlapping the roe deer hunting periods of the majority of European countries [22]. This indicator is therefore able to capture the system’s complexities while remaining simple enough to be easily and routinely used [81–82].

## Supporting information

S1 Dataset Data collection. Roe deer mandibular data (sheet 1), offspring and adult female data (sheet 2), hunting ground and population data (sheet 3), remote sensing data (sheet 4) and climate data (sheet 5) used in the present study.
(XLS)

S1 Fig Study area. Map of the study site located in Arezzo province (43° 28′ N, 11° 53′ E; in black in the left panel), Tuscany (in grey in the left panel), Central Italy. This area includes 1,910 hunting zones where 24,972 roe deer (12,026 females and 12,946 males) were legally shot during the annual harvest (August 1st—September 30th and January 1st—March 15th) from 2005 to 2015. Hunting zones were divided into three elevation classes and the percentage of territory covered by each class is shown in brackets. Red points represent centroids of a network of 187 permanent sample areas monitored by drive censuses in May and June (0.44 km2 ± 0.26 S.D. on a total area of 81.16 km2).
(DOCX)

S1 Table Age classes in roe deer. Diagnostic characters used to identify age classes observing molar arcade. These characters were selected examining mandibles (N = 330) collected in Northern Apennines (Arezzo province, Tuscany, Central Italy; Capitani et al. 2005) and aged counting cementum layers on M1 root.
(DOCX)

S2 Table Sample size and mandible growth rate. Mandible total length (mean value [X] and standard deviation [SD]) for 24,972 roe deer (12,026 females and 12,946 males) legally shot during the annual harvest (August 1st—September 30th and January 1st—March 15th) from 2005 to 2015 in Arezzo province (Tuscany, Central Italy). The percentage of total length is reported for each age class both for males and females.
(DOCX)

S3 Table Data collection on mandible measurements. Anterior and posterior sections on a sample of 2,161 mandibles of juveniles (8–10 months; see Fig 1 in the paper for major details about how these measurements were obtained), collected during the annual harvest (August 1st—September 30th and January 1st—March 15th) from 2013 to 2015 in Arezzo province (Tuscany, Central Italy).
(DOCX)

S4 Table List and description of independent variables. Variables associated to roe deer mandible growth recorded in the 2013–2015 period in each hunting zone of Arezzo province (Tuscany, Central Italy). Pregnancy corresponds to post-diapause period.
(DOCX)

S5 Table Network of permanent monitoring sample areas for roe deer. Hunting area, number, extent and percentage of areas monitored by drive censuses in each hunting district of the Arezzo province (Tuscany, Central Italy) in May and June. See S1 Fig for the location of the network of permanent sample areas.
(DOCX)

S6 Table Correlation matrix for selected independent variables. Pearson correlation coefficient (rp) among environmental and climatic variables associated to roe deer mandible growth recorded in the 2013–2015 period in each hunting ground of Arezzo province (Tuscany, Central Italy). See S4 Table for the description of each variable.
(DOCX)

S7 Table Set of most parsimonious models. Top model set containing models explaining the variation in the length of the anterior (A) and posterior (B) sections of the mandible for juvenile roe deer (8–10 months) hunted within the Arezzo Province from January to March over three consecutive hunting seasons (2013–2015) with a ΔAIC that is ≤2. The best model in bold. [AIC = Akaike Information Criterion; 𝚫AIC = AIC (model)–AIC (best model); Wt = AIC weights]. See S4 Table for the description of independent variables.
(DOCX)
