# Supplementary material for: Ecological conditions experienced by offspring during pregnancy and early post-natal life determine mandible size in roe deer
Source: PLoS One. 2019 Sep 11;14(9):e0222150. doi: 10.1371/journal.pone.0222150 (PMC6738612; doi:10.1371/journal.pone.0222150)

**Ecological conditions experienced by offspring during pregnancy and early post-natal life determine mandible size in roe deer.**

PLoS ONE

Anna Maria De Marinis, Roberta Chirichella^*^, Elisa Bottero, Marco Apollonio

** Department of Veterinary Medicine, University of Sassari, via Vienna 2, I-07100 Sassari, Italy;* [*rchirichella@uniss.it*](mailto:rchirichella@uniss.it)

**S1 Fig. Study area.** Map of the study site located in Arezzo province (43° 28′ N, 11° 53′ E; in black in the left panel), Tuscany (in grey in the left panel), Central Italy. This area includes 1,910 hunting zones where 24,972 roe deer (12,026 females and 12,946 males) were legally shot during the annual harvest (August 1^st^ - September 30^th^ and January 1^st^ - March 15^th^) from 2005 to 2015. Hunting zones were divided into three elevation classes and the percentage of territory covered by each class is shown in brackets. Red points represent centroids of a network of 187 permanent sample areas monitored by drive censuses in May and June (0.44 km^2^ ± 0.26 S.D. on a total area of 81.16 km^2^).


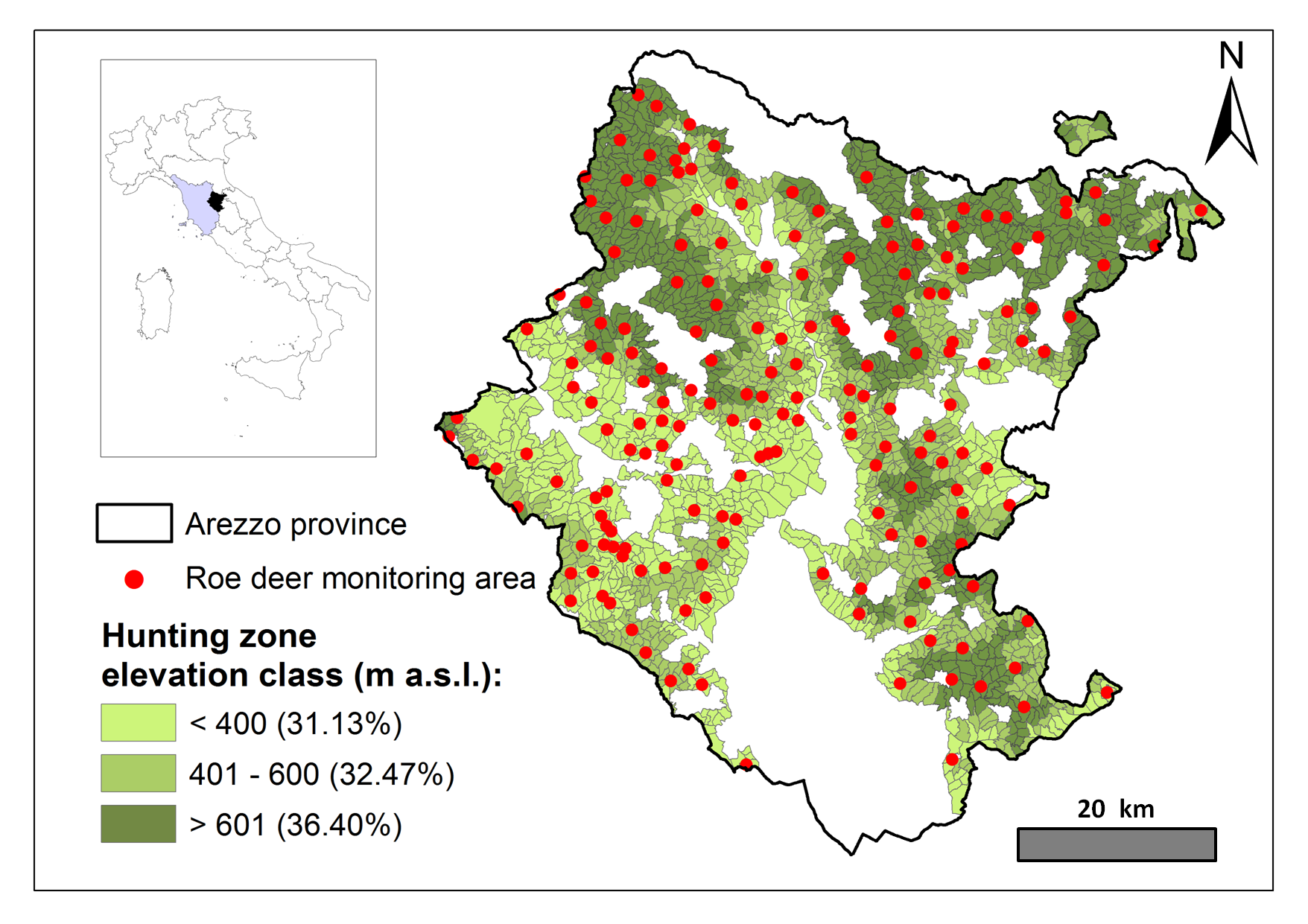

Supplement: S1 Fig — Map of the study site located in Arezzo province (43° 28′ N, 11° 53′ E; in black in the left panel), Tuscany (in grey in the left panel), Central Italy. This area includes 1,910 hunting zones where 24,972 roe deer (12,026 females and 12,946 males) were legally shot during the annual harvest (August 1st—September 30th and January 1st—March 15th) from 2005 to 2015. Hunting zones were divided into three elevation classes and the percentage of territory covered by each class is shown in brackets. Red points represent centroids of a network of 187 permanent sample areas monitored by drive censuses in May and June (0.44 km2 ± 0.26 S.D. on a total area of 81.16 km2). (DOCX) [file pone.0222150.s002.docx]
