# Supplementary material for: Ecological conditions experienced by offspring during pregnancy and early post-natal life determine mandible size in roe deer
Source: PLoS One. 2019 Sep 11;14(9):e0222150. doi: 10.1371/journal.pone.0222150 (PMC6738612; doi:10.1371/journal.pone.0222150)
Supplement: S1 Table — Diagnostic characters used to identify age classes observing molar arcade. These characters were selected examining mandibles (N = 330) collected in Northern Apennines (Arezzo province, Tuscany, Central Italy; Capitani et al. 2005) and aged counting cementum layers on M1 root. (DOCX) [file pone.0222150.s003.DOCX]

**Ecological conditions experienced by offspring during pregnancy and early post-natal life determine mandible size in roe deer.**

PLoS ONE

Anna Maria De Marinis, Roberta Chirichella^*^, Elisa Bottero, Marco Apollonio

** Department of Veterinary Medicine, University of Sassari, via Vienna 2, I-07100 Sassari, Italy;* [*rchirichella@uniss.it*](mailto:rchirichella@uniss.it)

**S1 Table. Age classes in roe deer.** Diagnostic characters used to identify age classes observing molar arcade. These characters were selected examining mandibles (N= 330) collected in Northern Apennines (Arezzo province, Tuscany, Central Italy; Capitani et al. 2005) and aged counting cementum layers on M_1_ root**.**

| **Age class (months)** | **Molar arcade** | | | | **Diagnostic character** | | | |  |
| --- | --- | --- | --- | --- | --- | --- | --- | --- | --- |
|  | Premolars | M_1._ | M_2_ | M_3_ | |  |  | |  |
| 3-4 | Deciduous | Present |  |  | | 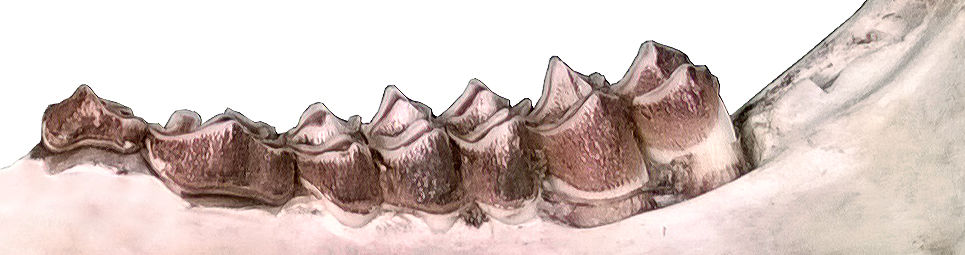 | | |  |
| 8-10 | Deciduous | Present | Present | Present not completely erupted | | 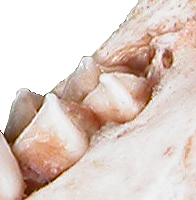  not protruded through the bone | | 5^th^ cusp of M_3_  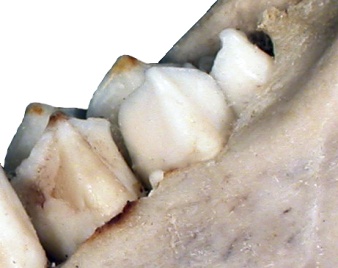  protruded but not fully and not colored  because still inside the gum |  |
| 15-16 | Permanent | Present | Present | Present completely erupted | | 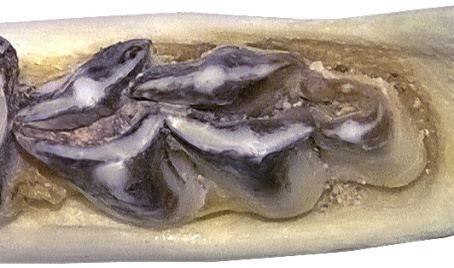  5^th^ cusp of M_3_ without exposed dentine | | | |
| ≥ 27 | Permanent | Present | Present | Present | | 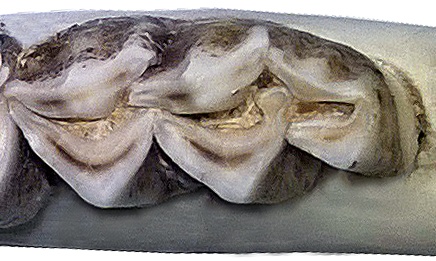  5^th^ cusp of M_3_ with exposed dentine | | | |

Capitani C, Mattioli L, Apollonio M. Progetto di monitoraggio integrato degli ungulati nei distretti di gestione appenninici della Provincia di Arezzo. 2005; Arezzo, Italy. [In Italian]
