# Supplementary material for: Ecological conditions experienced by offspring during pregnancy and early post-natal life determine mandible size in roe deer
Source: PLoS One. 2019 Sep 11;14(9):e0222150. doi: 10.1371/journal.pone.0222150 (PMC6738612; doi:10.1371/journal.pone.0222150)
Supplement: S2 Table — Mandible total length (mean value [X] and standard deviation [SD]) for 24,972 roe deer (12,026 females and 12,946 males) legally shot during the annual harvest (August 1st—September 30th and January 1st—March 15th) from 2005 to 2015 in Arezzo province (Tuscany, Central Italy). The percentage of total length is reported for each age class both for males and females. (DOCX) [file pone.0222150.s004.docx]

**Ecological conditions experienced by offspring during pregnancy and early post-natal life determine mandible size in roe deer.**

PLoS ONE

Anna Maria De Marinis, Roberta Chirichella^*^, Elisa Bottero, Marco Apollonio

** Department of Veterinary Medicine, University of Sassari, via Vienna 2, I-07100 Sassari, Italy;* [*rchirichella@uniss.it*](mailto:rchirichella@uniss.it)

**S2 Table. Sample size and mandible growth rate.** Mandible total length (mean value [X] and standard deviation [SD]) for 24,972 roe deer (12,026 females and 12,946 males) legally shot during the annual harvest (August 1^st^ - September 30^th^ and January 1^st^ - March 15^th^) from 2005 to 2015 in Arezzo province (Tuscany, Central Italy). The percentage of total length is reported for each age class both for males and females.

|  |  |  | **Mandible total length (mm)** | | | |
| --- | --- | --- | --- | --- | --- | --- |
| **Age class (months)** | **Sex** | **N** | **X** | **SD** | **%** |  |
| 3-4 | **♂** | 2,437 | 117.16 | 6.76 | 73.13 |  |
|  | **♀** | 2,131 | 116.30 | 6.36 | 74.82 |  |
| 8-10 | **♂** | 1,804 | 142.30 | 5.01 | 89.43 |  |
|  | **♀** | 2,091 | 140.11 | 4.86 | 90.02 |  |
| 15-16 | **♂** | 3,004 | 152.11 | 4.82 | 96.25 |  |
|  | **♀** | 1,848 | 150.19 | 4.82 | 96.62 |  |
| ≥27 | **♂** | 5,701 | 158.04 | 4.96 | 100.00 |  |
|  | **♀** | 5,956 | 155.44 | 4.80 | 100.00 |  |
