# Supplementary material for: Ecological conditions experienced by offspring during pregnancy and early post-natal life determine mandible size in roe deer
Source: PLoS One. 2019 Sep 11;14(9):e0222150. doi: 10.1371/journal.pone.0222150 (PMC6738612; doi:10.1371/journal.pone.0222150)
Supplement: S3 Table — Anterior and posterior sections on a sample of 2,161 mandibles of juveniles (8–10 months; see Fig 1 in the paper for major details about how these measurements were obtained), collected during the annual harvest (August 1st—September 30th and January 1st—March 15th) from 2013 to 2015 in Arezzo province (Tuscany, Central Italy). (DOCX) [file pone.0222150.s005.docx]

**Ecological conditions experienced by offspring during pregnancy and early post-natal life determine mandible size in roe deer.**

PLoS ONE

Anna Maria De Marinis, Roberta Chirichella^*^, Elisa Bottero, Marco Apollonio

** Department of Veterinary Medicine, University of Sassari, via Vienna 2, I-07100 Sassari, Italy;* [*rchirichella@uniss.it*](mailto:rchirichella@uniss.it)

**S3 Table. Data collection on mandible measurements.** Anterior and posterior sections on a sample of 2,161 mandibles of juveniles (8-10 months; see Fig. 1 in the paper for major details about how these measurements were obtained), collected during he annual harvest (August 1^st^ - September 30^th^ and January 1^st^ - March 15^th^) from 2013 to 2015 in Arezzo province (Tuscany, Central Italy).

|  |  |  |  |  | **Mandible measurements (mm)** | |
| --- | --- | --- | --- | --- | --- | --- |
| **Roe deer ID** | **Hunting district** | **Julian date** | **Year** | **Sex** | **Anterior section** | **Posterior section** |
| 1 | #6 | 24 | 2015 | Female | 70.50 | 55.50 |
| 2 | #4 | 1 | 2014 | Female | 64.19 | 51.09 |
| 3 | #4 | 60 | 2014 | Female | 64.45 | 72.70 |
| 4 | #6 | 5 | 2015 | Male | 66.04 | 50.68 |
| 5 | #4 | 25 | 2014 | Female | 67.14 | 63.14 |
| 6 | #4 | 9 | 2014 | Male | 63.11 | 62.30 |
| 7 | #4 | 50 | 2014 | Female | 64.18 | 70.83 |
| 8 | #4 | 43 | 2015 | Female | 67.06 | 65.92 |
| 9 | #4 | 62 | 2013 | Male | 54.23 | 69.99 |
| 10 | #4 | 47 | 2014 | Male | 56.79 | 73.29 |
| 11 | #4 | 3 | 2013 | Male | 64.50 | 47.51 |
| 12 | #4 | 3 | 2013 | Female | 58.61 | 44.36 |
| 13 | #4 | 12 | 2013 | Female | 63.96 | 46.31 |
| 14 | #4 | 10 | 2013 | Male | 68.60 | 58.86 |
| 15 | #5 | 54 | 2014 | Female | 66.38 | 64.48 |
| 16 | #5 | 6 | 2014 | Male | 67.54 | 56.11 |
| 17 | #5 | 7 | 2013 | Female | 67.12 | 52.12 |
| 18 | #5 | 45 | 2013 | Female | 66.06 | 63.30 |
| 19 | #5 | 45 | 2013 | Male | 66.01 | 63.96 |
| 20 | #5 | 31 | 2013 | Female | 67.03 | 65.58 |
| 21 | #5 | 12 | 2013 | Male | 66.43 | 59.45 |
| 22 | #5 | 61 | 2014 | Male | 68.18 | 68.78 |
| 23 | #5 | 59 | 2013 | Male | 67.85 | 69.43 |
| 24 | #6 | 50 | 2014 | Male | 64.88 | 64.13 |
| 25 | #6 | 25 | 2014 | Female | 57.98 | 66.00 |
| 26 | #4 | 5 | 2015 | Male | NA | NA |
| 27 | #6 | 54 | 2014 | Female | 65.59 | 67.42 |
| 28 | #6 | 4 | 2015 | Male | 70.20 | 62.81 |
| 29 | #6 | 38 | 2013 | Female | 62.54 | 63.44 |
| 30 | #6 | 70 | 2013 | Female | 64.16 | 66.08 |
| 31 | #6 | 43 | 2015 | Female | NA | NA |
| 32 | #6 | 73 | 2013 | Female | 65.21 | 67.30 |
| 33 | #6 | 12 | 2013 | Female | 63.89 | 56.26 |
| 34 | #6 | 47 | 2013 | Female | 65.39 | 67.73 |
| 35 | #6 | 22 | 2014 | Male | 68.18 | 58.92 |
| 36 | #6 | 26 | 2014 | Female | 64.82 | 56.98 |
| 37 | #6 | 64 | 2015 | Female | 67.95 | 69.29 |
| 38 | #6 | 51 | 2013 | Female | 66.54 | 66.93 |
| 39 | #6 | 5 | 2013 | Female | 58.14 | 54.65 |
| 40 | #6 | 26 | 2013 | Female | 65.01 | 63.24 |
| 41 | #6 | 69 | 2013 | Female | 68.54 | 69.63 |
| 42 | #6 | 50 | 2015 | Male | 54.17 | 65.22 |
| 43 | #6 | 65 | 2013 | Female | 68.12 | 71.02 |
| 44 | #6 | 66 | 2013 | Female | 67.85 | 71.76 |
| 45 | #6 | 73 | 2013 | Male | 69.01 | 69.13 |
| 46 | #6 | 68 | 2015 | Male | 65.71 | 64.62 |
| 47 | #6 | 20 | 2014 | Female | 70.09 | 56.38 |
| 48 | #6 | 61 | 2014 | Female | 66.59 | 69.92 |
| 49 | #6 | 4 | 2015 | Male | 71.24 | 62.02 |
| 50 | #6 | 74 | 2014 | Female | 66.43 | 73.80 |
| 51 | #6 | 23 | 2014 | Female | 64.28 | 58.84 |
| 52 | #6 | 69 | 2014 | Female | 67.68 | 72.08 |
| 53 | #6 | 67 | 2014 | Female | 57.41 | 63.55 |
| 54 | #6 | 44 | 2014 | Female | 66.52 | 69.47 |
| 55 | #7 | 34 | 2014 | Female | 66.12 | 67.86 |
| 56 | #7 | 57 | 2014 | Male | 67.87 | 66.85 |
| 57 | #7 | 3 | 2013 | Male | 66.54 | 56.91 |
| 58 | #7 | 43 | 2014 | Female | 67.32 | 66.55 |
| 59 | #7 | 51 | 2014 | Female | 67.34 | 65.65 |
| 60 | #7 | 4 | 2015 | Female | 68.81 | 48.85 |
| 61 | #7 | 68 | 2013 | Male | 67.87 | 66.67 |
| 62 | #7 | 5 | 2013 | Male | 66.87 | 58.11 |
| 63 | #7 | 6 | 2013 | Female | 66.44 | 54.32 |
| 64 | #7 | 62 | 2014 | Female | 66.53 | 71.47 |
| 65 | #7 | 72 | 2013 | Male | 67.19 | 66.79 |
| 66 | #7 | 72 | 2014 | Male | 67.81 | 68.67 |
| 67 | #18 | 54 | 2014 | Female | 68.14 | 70.75 |
| 68 | #18 | 37 | 2013 | Male | 67.50 | 65.99 |
| 69 | #18 | 18 | 2014 | Male | 66.83 | 59.15 |
| 70 | #18 | 50 | 2015 | Female | 64.99 | 67.57 |
| 71 | #18 | 72 | 2013 | Female | 65.78 | 70.16 |
| 72 | #18 | 35 | 2013 | Male | 65.39 | 68.50 |
| 73 | #18 | 53 | 2014 | Male | 67.37 | 67.17 |
| 74 | #18 | 2 | 2013 | Female | 61.59 | 54.42 |
| 75 | #18 | 36 | 2014 | Male | 65.21 | 67.18 |
| 76 | #18 | 53 | 2014 | Female | 68.32 | 67.33 |
| 77 | #18 | 61 | 2013 | Male | 68.81 | 68.20 |
| 78 | #18 | 63 | 2013 | Female | 64.25 | 66.09 |
| 79 | #18 | 63 | 2015 | Female | 64.60 | 58.05 |
| 80 | #18 | 16 | 2014 | Male | 67.92 | 59.03 |
| 81 | #18 | 56 | 2014 | Male | 68.72 | 67.29 |
| 82 | #18 | 2 | 2014 | Female | 65.72 | 58.03 |
| 83 | #18 | 52 | 2013 | Female | 66.90 | 66.81 |
| 84 | #18 | 50 | 2015 | Female | 63.27 | 67.11 |
| 85 | #18 | 40 | 2013 | Female | 69.60 | 67.79 |
| 86 | #18 | 69 | 2013 | Female | 68.75 | 73.10 |
| 87 | #18 | 27 | 2013 | Male | 63.72 | 58.89 |
| 88 | #2 | 13 | 2013 | Female | 61.83 | 56.45 |
| 89 | #2 | 72 | 2013 | Female | 63.70 | 70.71 |
| 90 | #2 | 16 | 2014 | Male | 67.88 | 62.36 |
| 91 | #2 | 50 | 2013 | Female | 63.93 | 64.38 |
| 92 | #2 | 67 | 2014 | Male | 67.81 | 69.71 |
| 93 | #2 | 21 | 2013 | Female | 68.14 | 61.79 |
| 94 | #2 | 66 | 2013 | Male | 65.71 | 65.29 |
| 95 | #2 | 23 | 2013 | Female | 66.60 | 60.62 |
| 96 | #2 | 67 | 2014 | Male | 67.34 | 68.98 |
| 97 | #2 | 68 | 2014 | Male | 67.73 | 66.13 |
| 98 | #2 | 65 | 2014 | Female | 66.47 | 72.20 |
| 99 | #2 | 61 | 2014 | Female | 65.12 | 68.53 |
| 100 | #2 | 53 | 2014 | Male | 67.31 | 68.42 |
| 101 | #2 | 2 | 2013 | Female | 66.18 | 47.27 |
| 102 | #2 | 69 | 2013 | Female | 65.07 | 64.30 |
| 103 | #2 | 17 | 2015 | Male | 69.04 | 56.87 |
| 104 | #2 | 72 | 2014 | Male | 66.89 | 72.06 |
| 105 | #2 | 19 | 2014 | Male | 67.42 | 63.64 |
| 106 | #2 | 29 | 2014 | Female | 66.14 | 61.37 |
| 107 | #2 | 42 | 2015 | Female | 69.72 | 63.36 |
| 108 | #2 | 33 | 2014 | Female | 65.18 | 62.69 |
| 109 | #2 | 46 | 2015 | Female | 66.99 | 62.01 |
| 110 | #2 | 11 | 2014 | Female | 64.12 | 56.09 |
| 111 | #2 | 11 | 2014 | Male | 66.71 | 60.13 |
| 112 | #2 | 52 | 2015 | Male | 71.13 | 66.23 |
| 113 | #2 | 55 | 2014 | Female | 68.17 | 70.30 |
| 114 | #2 | 64 | 2014 | Female | 64.23 | 72.59 |
| 115 | #2 | 14 | 2015 | Female | 64.99 | 55.39 |
| 116 | #2 | 12 | 2014 | Female | 67.43 | 59.99 |
| 117 | #2 | 15 | 2014 | Male | 66.58 | 58.28 |
| 118 | #2 | 39 | 2014 | Female | 66.54 | 63.28 |
| 119 | #2 | 28 | 2015 | Male | 71.43 | 65.29 |
| 120 | #2 | 8 | 2014 | Male | 65.00 | 58.89 |
| 121 | #2 | 22 | 2014 | Male | 68.79 | 64.89 |
| 122 | #2 | 46 | 2014 | Male | 66.48 | 66.35 |
| 123 | #3 | 73 | 2013 | Male | 66.15 | 66.97 |
| 124 | #3 | 69 | 2013 | Male | 67.45 | 66.00 |
| 125 | #3 | 57 | 2014 | Female | 66.33 | 67.98 |
| 126 | #3 | 61 | 2013 | Male | 67.89 | 65.98 |
| 127 | #3 | 63 | 2013 | Male | 67.43 | 67.13 |
| 128 | #3 | 68 | 2014 | Male | 67.17 | 66.28 |
| 129 | #3 | 46 | 2014 | Male | 67.58 | 66.24 |
| 130 | #3 | 72 | 2013 | Male | 67.64 | 69.12 |
| 131 | #3 | 72 | 2013 | Female | 67.38 | 70.33 |
| 132 | #3 | 58 | 2014 | Male | 66.12 | 70.59 |
| 133 | #3 | 63 | 2013 | Male | 66.51 | 68.70 |
| 134 | #3 | 68 | 2013 | Female | 66.32 | 69.50 |
| 135 | #3 | 47 | 2013 | Male | 66.21 | 66.34 |
| 136 | #3 | 61 | 2014 | Male | 66.69 | 70.23 |
| 137 | #3 | 44 | 2014 | Female | 67.09 | 66.99 |
| 138 | #3 | 39 | 2014 | Male | 67.87 | 67.11 |
| 139 | #3 | 65 | 2014 | Male | 67.45 | 69.30 |
| 140 | #3 | 12 | 2014 | Female | 66.43 | 57.13 |
| 141 | #3 | 65 | 2014 | Male | 67.53 | 71.47 |
| 142 | #3 | 33 | 2014 | Male | 67.52 | 63.24 |
| 143 | #3 | 69 | 2013 | Female | 66.89 | 66.96 |
| 144 | #3 | 49 | 2013 | Male | 66.78 | 66.27 |
| 145 | #3 | 23 | 2014 | Female | 66.83 | 61.63 |
| 146 | #3 | 47 | 2014 | Female | 67.78 | 64.77 |
| 147 | #4 | 40 | 2014 | Male | 57.81 | 60.89 |
| 148 | #7 | 39 | 2014 | Female | 68.12 | 63.75 |
| 149 | #2 | 68 | 2014 | Male | 66.29 | 69.63 |
| 150 | #2 | 11 | 2014 | Female | 66.46 | 56.82 |
| 151 | #2 | 71 | 2015 | Female | 68.97 | 68.00 |
| 152 | #2 | 2 | 2014 | Male | 68.75 | 51.81 |
| 153 | #2 | 25 | 2014 | Male | 65.28 | 64.33 |
| 154 | #2 | 26 | 2013 | Female | 68.53 | 60.04 |
| 155 | #3 | 71 | 2014 | Male | 67.30 | 66.33 |
| 156 | #12 | 65 | 2014 | Female | 66.74 | 67.68 |
| 157 | #12 | 25 | 2014 | Male | 67.23 | 58.64 |
| 158 | #9 | 22 | 2014 | Male | 70.01 | 65.75 |
| 159 | #9 | 9 | 2013 | Female | 66.32 | 52.33 |
| 160 | #21 | 65 | 2013 | Female | 66.35 | 67.56 |
| 161 | #10 | 37 | 2013 | Male | 66.26 | 68.28 |
| 162 | #10 | 74 | 2015 | Female | 68.45 | 67.22 |
| 163 | #10 | 19 | 2013 | Female | 67.88 | 55.36 |
| 164 | #1 | 24 | 2013 | Female | 67.43 | 64.99 |
| 165 | #11 | 68 | 2013 | Female | 68.76 | 71.11 |
| 166 | #11 | 25 | 2014 | Female | 66.23 | 62.31 |
| 167 | #11 | 20 | 2013 | Female | 66.58 | 56.28 |
| 168 | #11 | 62 | 2013 | Female | 67.88 | 70.36 |
| 169 | #14 | 62 | 2013 | Female | 67.42 | 71.64 |
| 170 | #14 | 73 | 2013 | Female | 63.73 | 65.95 |
| 171 | #14 | 73 | 2013 | Male | 65.28 | 70.33 |
| 172 | #14 | 72 | 2013 | Female | 66.91 | 65.71 |
| 173 | #14 | 58 | 2013 | Female | 65.29 | 72.62 |
| 174 | #14 | 27 | 2013 | Male | 63.52 | 59.86 |
| 175 | #14 | 43 | 2015 | Male | 64.19 | 62.92 |
| 176 | #14 | 73 | 2015 | Male | 68.13 | 57.21 |
| 177 | #21 | 46 | 2014 | Male | 66.87 | 64.78 |
| 178 | #21 | 70 | 2013 | Male | 64.96 | 64.86 |
| 179 | #21 | 68 | 2013 | Female | 68.27 | 66.46 |
| 180 | #21 | 69 | 2013 | Female | 63.75 | 72.30 |
| 181 | #21 | 71 | 2014 | Male | 66.33 | 59.86 |
| 182 | #21 | 39 | 2015 | Female | 67.94 | 66.81 |
| 183 | #18 | 26 | 2014 | Female | 65.91 | 63.41 |
| 184 | #17 | 68 | 2013 | Male | 66.85 | 71.52 |
| 185 | #17 | 74 | 2014 | Male | 65.48 | 69.81 |
| 186 | #17 | 65 | 2013 | Male | 65.45 | 67.69 |
| 187 | #17 | 72 | 2014 | Female | 65.82 | 63.82 |
| 188 | #17 | 43 | 2014 | Male | 65.14 | 62.65 |
| 189 | #12 | 51 | 2014 | Female | 66.32 | 71.70 |
| 190 | #3 | 47 | 2014 | Female | 67.47 | 65.55 |
| 191 | #3 | 57 | 2014 | Male | 67.43 | 68.55 |
| 192 | #3 | 47 | 2014 | Male | 67.91 | 64.80 |
| 193 | #3 | 71 | 2015 | Female | 68.15 | 65.11 |
| 194 | #3 | 40 | 2015 | Male | 67.86 | 67.02 |
| 195 | #3 | 65 | 2013 | Female | 67.43 | 69.99 |
| 196 | #3 | 61 | 2014 | Female | 67.97 | 66.24 |
| 197 | #2 | 70 | 2013 | Male | 66.34 | 67.38 |
| 198 | #2 | 14 | 2013 | Female | 66.58 | 50.27 |
| 199 | #2 | 72 | 2013 | Female | 67.88 | 70.36 |
| 200 | #2 | 11 | 2014 | Male | 67.33 | 61.46 |
| 201 | #2 | 64 | 2014 | Female | 65.72 | 67.72 |
| 202 | #2 | 68 | 2013 | Male | 67.42 | 69.43 |
| 203 | #2 | 50 | 2015 | Female | 68.38 | 66.42 |
| 204 | #2 | 44 | 2014 | Female | 65.32 | 65.37 |
| 205 | #2 | 69 | 2014 | Female | 67.44 | 69.43 |
| 206 | #2 | 66 | 2013 | Male | 63.73 | 65.95 |
| 207 | #9 | 40 | 2013 | Female | 63.74 | 58.17 |
| 208 | #9 | 52 | 2013 | Male | 63.52 | 65.86 |
| 209 | #9 | 41 | 2013 | Female | 66.91 | 64.71 |
| 210 | #9 | 39 | 2015 | Female | 71.21 | 68.13 |
| 211 | #9 | 3 | 2013 | Female | 65.28 | 51.36 |
| 212 | #1 | 55 | 2014 | Female | 66.92 | 72.11 |
| 213 | #1 | 62 | 2013 | Female | 64.96 | 71.86 |
| 214 | #1 | 47 | 2013 | Female | 67.42 | 70.64 |
| 215 | #1 | 59 | 2013 | Female | 63.73 | 65.95 |
| 216 | #1 | 72 | 2014 | Female | 66.59 | 71.84 |
| 217 | #1 | 70 | 2015 | Female | 70.81 | 70.70 |
| 218 | #1 | 51 | 2013 | Female | 65.28 | 70.33 |
| 219 | #1 | 73 | 2013 | Male | 68.27 | 70.46 |
| 220 | #1 | 34 | 2013 | Female | 66.91 | 63.71 |
| 221 | #19 | 62 | 2014 | Male | 66.65 | 67.33 |
| 222 | #19 | 5 | 2015 | Female | 65.97 | 52.91 |
| 223 | #19 | 39 | 2014 | Female | 67.56 | 66.52 |
| 224 | #19 | 12 | 2013 | Male | 66.34 | 59.38 |
| 225 | #8 | 7 | 2013 | Male | 66.58 | 56.28 |
| 226 | #10 | 72 | 2014 | Male | 66.11 | 67.92 |
| 227 | #10 | 65 | 2013 | Male | 67.76 | 70.68 |
| 228 | #10 | 19 | 2013 | Male | 67.54 | 57.91 |
| 229 | #10 | 52 | 2013 | Male | 65.87 | 67.31 |
| 230 | #14 | 47 | 2013 | Male | 65.28 | 69.33 |
| 231 | #14 | 35 | 2013 | Male | 66.91 | 62.71 |
| 232 | #11 | 47 | 2013 | Male | 63.74 | 66.17 |
| 233 | #11 | 58 | 2013 | Female | 70.19 | 78.99 |
| 234 | #11 | 35 | 2013 | Female | 64.96 | 69.86 |
| 235 | #21 | 65 | 2014 | Female | 66.29 | 67.09 |
| 236 | #21 | 65 | 2014 | Female | 67.83 | 70.06 |
| 237 | #21 | 3 | 2013 | Female | 68.27 | 41.46 |
| 238 | #21 | 67 | 2014 | Male | 67.53 | 65.23 |
| 239 | #21 | 58 | 2013 | Male | 63.75 | 72.30 |
| 240 | #21 | 73 | 2013 | Male | 67.33 | 64.46 |
| 241 | #21 | 43 | 2014 | Male | 66.94 | 65.38 |
| 242 | #13 | 40 | 2013 | Male | 67.88 | 67.36 |
| 243 | #13 | 50 | 2014 | Male | 63.82 | 61.53 |
| 244 | #13 | 65 | 2014 | Female | 68.21 | 68.62 |
| 245 | #15 | 61 | 2013 | Male | 66.17 | 66.47 |
| 246 | #20 | 18 | 2015 | Male | 67.98 | 54.48 |
| 247 | #10 | 31 | 2013 | Female | 67.23 | 68.64 |
| 248 | #10 | 1 | 2014 | Male | 66.12 | 57.53 |
| 249 | #10 | 41 | 2014 | Female | 66.21 | 66.45 |
| 250 | #10 | 39 | 2014 | Male | 66.25 | 64.18 |
| 251 | #10 | 30 | 2013 | Female | 67.42 | 63.67 |
| 252 | #10 | 52 | 2013 | Male | 66.31 | 66.31 |
| 253 | #4 | 72 | 2013 | Female | 64.01 | 67.30 |
| 254 | #5 | 27 | 2014 | Male | 67.18 | 60.87 |
| 255 | #5 | 47 | 2015 | Female | 69.81 | 66.75 |
| 256 | #22 | 32 | 2014 | Female | 66.31 | 61.47 |
| 257 | #22 | 68 | 2014 | Female | 67.49 | 61.30 |
| 258 | #22 | 61 | 2014 | Female | 66.58 | 59.09 |
| 259 | #22 | 59 | 2015 | Male | 68.41 | 72.96 |
| 260 | #7 | 57 | 2014 | Female | 67.32 | 66.12 |
| 261 | #18 | 63 | 2013 | Male | 63.73 | 65.95 |
| 262 | #8 | 68 | 2013 | Female | 68.23 | 80.89 |
| 263 | #8 | 72 | 2013 | Male | 68.61 | 70.46 |
| 264 | #8 | 68 | 2013 | Female | 68.32 | 70.84 |
| 265 | #8 | 41 | 2013 | Female | 63.74 | 66.17 |
| 266 | #8 | 71 | 2014 | Female | 68.24 | 73.28 |
| 267 | #8 | 26 | 2013 | Female | 65.28 | 65.33 |
| 268 | #15 | 41 | 2013 | Female | 69.75 | 69.44 |
| 269 | #15 | 4 | 2015 | Male | 68.12 | 62.04 |
| 270 | #15 | 63 | 2015 | Male | 68.27 | 71.99 |
| 271 | #15 | 26 | 2014 | Male | 64.25 | 61.90 |
| 272 | #15 | 20 | 2014 | Female | 63.64 | 57.38 |
| 273 | #15 | 30 | 2014 | Male | 64.91 | 60.65 |
| 274 | #15 | 52 | 2013 | Female | 65.43 | 70.06 |
| 275 | #15 | 60 | 2014 | Male | 70.16 | 74.22 |
| 276 | #15 | 51 | 2013 | Male | 68.74 | 74.41 |
| 277 | #15 | 27 | 2013 | Female | 65.83 | 66.29 |
| 278 | #15 | 25 | 2014 | Male | 63.94 | 55.40 |
| 279 | #15 | 41 | 2013 | Female | 69.14 | 71.43 |
| 280 | #15 | 62 | 2013 | Female | 66.34 | 67.38 |
| 281 | #16 | 26 | 2014 | Female | 66.78 | 60.40 |
| 282 | #16 | 19 | 2013 | Female | 66.58 | 56.57 |
| 283 | #16 | 65 | 2013 | Male | 64.21 | 65.63 |
| 284 | #16 | 72 | 2013 | Female | 70.19 | 78.96 |
| 285 | #16 | 73 | 2014 | Female | 65.01 | 68.15 |
| 286 | #16 | 28 | 2013 | Female | 66.53 | 61.36 |
| 287 | #16 | 72 | 2013 | Female | 64.98 | 68.30 |
| 288 | #9 | 73 | 2013 | Male | 64.96 | 71.86 |
| 289 | #9 | 48 | 2013 | Female | 63.74 | 66.17 |
| 290 | #9 | 44 | 2013 | Male | 65.19 | 68.93 |
| 291 | #9 | 65 | 2014 | Male | 68.76 | 68.77 |
| 292 | #9 | 61 | 2014 | Female | 67.43 | 73.33 |
| 293 | #9 | 70 | 2013 | Female | 68.27 | 70.46 |
| 294 | #9 | 67 | 2014 | Male | 66.71 | 71.10 |
| 295 | #9 | 16 | 2014 | Male | 68.16 | 62.81 |
| 296 | #9 | 69 | 2013 | Female | 63.75 | 67.30 |
| 297 | #9 | 69 | 2013 | Male | 67.33 | 69.46 |
| 298 | #9 | 64 | 2014 | Female | 66.92 | 71.10 |
| 299 | #9 | 55 | 2014 | Female | 69.11 | 70.46 |
| 300 | #9 | 57 | 2014 | Female | 67.92 | 69.95 |
| 301 | #9 | 30 | 2013 | Male | 66.91 | 59.71 |
| 302 | #9 | 69 | 2013 | Female | 65.91 | 69.46 |
| 303 | #9 | 69 | 2013 | Male | 67.43 | 69.99 |
| 304 | #9 | 19 | 2014 | Male | 67.43 | 57.55 |
| 305 | #9 | 34 | 2014 | Male | 67.22 | 67.32 |
| 306 | #9 | 40 | 2014 | Female | 66.13 | 64.85 |
| 307 | #9 | 46 | 2014 | Male | 67.08 | 65.55 |
| 308 | #9 | 26 | 2014 | Female | 66.98 | 62.23 |
| 309 | #1 | 3 | 2013 | Male | 64.96 | 61.86 |
| 310 | #1 | 27 | 2013 | Female | 68.27 | 65.46 |
| 311 | #1 | 69 | 2013 | Female | 63.75 | 67.30 |
| 312 | #1 | 62 | 2013 | Male | 67.33 | 69.46 |
| 313 | #1 | 72 | 2014 | Male | 67.38 | 68.69 |
| 314 | #1 | 41 | 2013 | Female | 63.74 | 66.17 |
| 315 | #1 | 72 | 2013 | Female | 65.91 | 69.46 |
| 316 | #1 | 56 | 2013 | Female | 63.52 | 65.86 |
| 317 | #1 | 37 | 2014 | Female | 67.88 | 67.28 |
| 318 | #1 | 36 | 2014 | Male | 66.93 | 63.43 |
| 319 | #1 | 72 | 2013 | Female | 67.43 | 69.99 |
| 320 | #1 | 61 | 2013 | Female | 66.34 | 67.38 |
| 321 | #1 | 61 | 2013 | Female | 66.58 | 70.31 |
| 322 | #1 | 40 | 2013 | Female | 64.96 | 69.86 |
| 323 | #1 | 32 | 2014 | Female | 66.54 | 64.85 |
| 324 | #1 | 26 | 2014 | Male | 66.41 | 61.57 |
| 325 | #1 | 68 | 2014 | Female | 68.02 | 66.21 |
| 326 | #1 | 62 | 2013 | Male | 67.88 | 70.36 |
| 327 | #1 | 30 | 2013 | Female | 63.75 | 62.30 |
| 328 | #1 | 47 | 2013 | Female | 68.27 | 69.46 |
| 329 | #1 | 72 | 2013 | Male | 66.25 | 70.07 |
| 330 | #1 | 40 | 2014 | Female | 66.44 | 71.82 |
| 331 | #1 | 64 | 2014 | Male | 67.51 | 68.13 |
| 332 | #1 | 61 | 2013 | Male | 67.46 | 71.80 |
| 333 | #1 | 51 | 2013 | Male | 63.75 | 67.30 |
| 334 | #1 | 47 | 2014 | Female | 66.32 | 71.13 |
| 335 | #1 | 68 | 2014 | Female | 67.48 | 66.44 |
| 336 | #1 | 32 | 2014 | Female | 66.12 | 67.49 |
| 337 | #1 | 32 | 2014 | Male | 67.89 | 67.58 |
| 338 | #1 | 72 | 2013 | Female | 64.79 | 66.15 |
| 339 | #1 | 21 | 2013 | Female | 67.33 | 64.46 |
| 340 | #1 | 21 | 2013 | Male | 65.91 | 63.46 |
| 341 | #1 | 59 | 2013 | Male | 67.33 | 69.46 |
| 342 | #1 | 59 | 2013 | Male | 65.91 | 69.46 |
| 343 | #1 | 73 | 2013 | Male | 66.78 | 66.28 |
| 344 | #1 | 67 | 2014 | Female | 69.12 | 68.89 |
| 345 | #1 | 73 | 2015 | Male | 69.22 | 72.42 |
| 346 | #1 | 67 | 2014 | Female | 66.55 | 67.01 |
| 347 | #1 | 72 | 2014 | Male | 67.98 | 65.52 |
| 348 | #1 | 69 | 2014 | Female | 67.08 | 68.18 |
| 349 | #1 | 69 | 2014 | Female | 67.37 | 66.18 |
| 350 | #1 | 61 | 2013 | Female | 67.55 | 67.66 |
| 351 | #1 | 67 | 2014 | Female | 66.25 | 67.49 |
| 352 | #1 | 58 | 2013 | Male | 67.43 | 69.99 |
| 353 | #1 | 59 | 2013 | Female | 66.34 | 67.38 |
| 354 | #1 | 39 | 2014 | Female | 67.96 | 68.45 |
| 355 | #1 | 61 | 2013 | Male | 69.12 | 70.40 |
| 356 | #1 | 42 | 2015 | Female | 66.10 | 71.90 |
| 357 | #1 | 59 | 2015 | Female | 69.17 | 71.95 |
| 358 | #1 | 54 | 2014 | Female | 67.03 | 69.02 |
| 359 | #1 | 72 | 2013 | Female | 66.75 | 66.69 |
| 360 | #1 | 5 | 2013 | Male | 67.43 | 59.99 |
| 361 | #1 | 47 | 2013 | Male | 66.58 | 65.28 |
| 362 | #1 | 61 | 2013 | Female | 67.16 | 68.60 |
| 363 | #1 | 12 | 2013 | Female | 66.34 | 57.38 |
| 364 | #1 | 48 | 2014 | Male | 69.24 | 63.37 |
| 365 | #1 | 48 | 2014 | Female | 68.26 | 64.20 |
| 366 | #1 | 50 | 2014 | Male | 67.43 | 67.55 |
| 367 | #1 | 55 | 2013 | Female | 67.88 | 70.36 |
| 368 | #1 | 14 | 2015 | Female | 67.95 | 55.15 |
| 369 | #12 | 57 | 2015 | Male | 67.05 | 64.34 |
| 370 | #12 | 55 | 2014 | Female | 65.98 | 71.17 |
| 371 | #12 | 72 | 2014 | Female | 66.92 | 67.28 |
| 372 | #12 | 39 | 2014 | Female | 66.78 | 64.56 |
| 373 | #12 | 74 | 2014 | Male | 66.80 | 67.07 |
| 374 | #12 | 23 | 2014 | Female | 66.12 | 62.31 |
| 375 | #12 | 23 | 2014 | Female | 66.13 | 57.84 |
| 376 | #12 | 56 | 2015 | Female | 67.14 | 71.33 |
| 377 | #12 | 59 | 2015 | Female | 68.67 | 66.04 |
| 378 | #12 | 33 | 2014 | Male | 66.51 | 66.16 |
| 379 | #2 | 25 | 2014 | Female | 66.91 | 60.71 |
| 380 | #2 | 68 | 2013 | Male | 67.42 | 71.64 |
| 381 | #2 | 25 | 2014 | Female | 63.74 | 62.17 |
| 382 | #2 | 55 | 2014 | Male | 68.33 | 67.60 |
| 383 | #2 | 51 | 2014 | Male | 68.03 | 68.04 |
| 384 | #2 | 72 | 2013 | Female | 63.73 | 65.95 |
| 385 | #2 | 55 | 2014 | Male | 67.24 | 66.78 |
| 386 | #2 | 58 | 2014 | Female | 66.47 | 67.28 |
| 387 | #2 | 65 | 2014 | Female | 66.92 | 72.13 |
| 388 | #2 | 66 | 2013 | Female | 65.28 | 70.33 |
| 389 | #2 | 74 | 2014 | Male | 69.79 | 70.89 |
| 390 | #2 | 72 | 2013 | Female | 66.91 | 65.71 |
| 391 | #2 | 49 | 2013 | Female | 63.74 | 66.17 |
| 392 | #2 | 30 | 2013 | Male | 63.52 | 59.86 |
| 393 | #2 | 5 | 2013 | Female | 64.96 | 55.86 |
| 394 | #2 | 40 | 2013 | Male | 68.27 | 67.46 |
| 395 | #2 | 68 | 2013 | Male | 63.75 | 67.30 |
| 396 | #2 | 30 | 2013 | Female | 67.33 | 60.36 |
| 397 | #2 | 10 | 2013 | Female | 65.91 | 53.46 |
| 398 | #2 | 61 | 2015 | Female | 69.23 | 63.12 |
| 399 | #2 | 68 | 2013 | Male | 67.43 | 69.99 |
| 400 | #2 | 26 | 2014 | Female | 64.96 | 66.86 |
| 401 | #2 | 26 | 2014 | Male | 68.27 | 64.46 |
| 402 | #2 | 3 | 2013 | Female | 66.34 | 58.38 |
| 403 | #2 | 7 | 2013 | Female | 66.58 | 50.28 |
| 404 | #2 | 39 | 2014 | Female | 66.43 | 64.99 |
| 405 | #2 | 74 | 2014 | Female | 67.75 | 69.47 |
| 406 | #2 | 59 | 2015 | Female | 68.15 | 69.93 |
| 407 | #2 | 19 | 2015 | Male | 71.15 | 63.89 |
| 408 | #2 | 31 | 2015 | Female | 65.19 | 63.83 |
| 409 | #2 | 73 | 2013 | Female | 67.88 | 70.36 |
| 410 | #2 | 72 | 2014 | Female | 67.85 | 71.27 |
| 411 | #17 | 34 | 2014 | Male | 61.03 | 61.66 |
| 412 | #17 | 37 | 2013 | Female | 68.87 | 60.85 |
| 413 | #17 | 71 | 2014 | Male | 63.27 | 65.70 |
| 414 | #17 | 27 | 2014 | Female | 68.79 | 71.82 |
| 415 | #17 | 34 | 2014 | Female | 67.61 | 60.53 |
| 416 | #17 | 56 | 2015 | Male | 66.25 | 72.23 |
| 417 | #17 | 34 | 2013 | Male | 60.28 | 62.70 |
| 418 | #17 | 27 | 2014 | Female | 68.73 | 70.53 |
| 419 | #17 | 71 | 2015 | Male | 66.31 | 74.38 |
| 420 | #17 | 64 | 2014 | Female | 69.98 | 70.57 |
| 421 | #17 | 18 | 2015 | Female | 64.25 | 54.89 |
| 422 | #17 | 33 | 2014 | Male | 66.97 | 63.31 |
| 423 | #17 | 37 | 2013 | Male | 59.36 | 61.94 |
| 424 | #17 | 70 | 2015 | Female | 65.49 | 74.32 |
| 425 | #17 | 53 | 2014 | Female | 62.01 | 64.33 |
| 426 | #17 | 49 | 2015 | Female | 66.87 | 67.42 |
| 427 | #20 | 47 | 2013 | Female | 66.17 | 59.70 |
| 428 | #20 | 49 | 2013 | Male | 67.58 | 64.57 |
| 429 | #20 | 40 | 2014 | Male | 65.41 | 64.24 |
| 430 | #20 | 33 | 2013 | Female | 66.32 | 63.82 |
| 431 | #20 | 33 | 2013 | Male | 66.87 | 61.40 |
| 432 | #18 | 64 | 2014 | Male | 67.34 | 66.65 |
| 433 | #18 | 71 | 2014 | Male | 67.49 | 68.26 |
| 434 | #18 | 24 | 2015 | Male | 67.50 | 66.42 |
| 435 | #18 | 74 | 2014 | Female | 68.60 | 68.27 |
| 436 | #18 | 70 | 2015 | Female | 64.74 | 73.45 |
| 437 | #18 | 74 | 2014 | Male | 65.57 | 73.51 |
| 438 | #18 | 40 | 2014 | Female | 64.01 | 62.33 |
| 439 | #18 | 72 | 2014 | Female | 65.63 | 67.61 |
| 440 | #18 | 11 | 2014 | Male | 65.23 | 59.31 |
| 441 | #18 | 16 | 2014 | Female | 70.02 | 58.45 |
| 442 | #6 | 63 | 2015 | Male | 64.16 | 69.43 |
| 443 | #6 | 61 | 2014 | Male | 66.27 | 68.97 |
| 444 | #6 | 38 | 2013 | Male | 66.23 | 64.89 |
| 445 | #6 | 1 | 2014 | Female | 68.21 | 45.94 |
| 446 | #6 | 2 | 2014 | Female | 65.76 | 49.40 |
| 447 | #7 | 69 | 2013 | Male | 63.75 | 67.30 |
| 448 | #7 | 70 | 2013 | Male | 67.33 | 69.46 |
| 449 | #7 | 67 | 2014 | Female | 67.33 | 66.19 |
| 450 | #7 | 70 | 2013 | Male | 65.91 | 69.46 |
| 451 | #4 | 63 | 2013 | Female | 65.69 | 66.01 |
| 452 | #6 | 52 | 2015 | Female | 65.20 | 64.93 |
| 453 | #3 | 61 | 2014 | Female | 68.17 | 71.01 |
| 454 | #3 | 73 | 2013 | Female | 66.34 | 67.38 |
| 455 | #3 | 17 | 2015 | Female | 69.62 | 57.65 |
| 456 | #3 | 69 | 2014 | Male | 66.43 | 71.38 |
| 457 | #3 | 53 | 2014 | Male | 66.79 | 66.20 |
| 458 | #3 | 72 | 2014 | Male | 66.37 | 69.65 |
| 459 | #3 | 32 | 2015 | Female | 73.12 | 69.25 |
| 460 | #3 | 67 | 2015 | Female | 65.46 | 67.61 |
| 461 | #3 | 70 | 2015 | Male | 67.12 | 63.63 |
| 462 | #13 | 61 | 2013 | Male | 65.91 | 69.46 |
| 463 | #13 | 72 | 2013 | Male | 67.43 | 69.99 |
| 464 | #13 | 71 | 2014 | Female | 67.41 | 68.59 |
| 465 | #13 | 73 | 2013 | Female | 66.34 | 67.38 |
| 466 | #13 | 6 | 2013 | Female | 65.91 | 53.46 |
| 467 | #13 | 68 | 2014 | Female | 65.15 | 70.53 |
| 468 | #13 | 61 | 2013 | Female | 66.58 | 66.28 |
| 469 | #13 | 69 | 2014 | Male | 69.14 | 69.88 |
| 470 | #13 | 62 | 2013 | Male | 67.88 | 70.36 |
| 471 | #13 | 69 | 2013 | Female | 67.42 | 71.64 |
| 472 | #13 | 71 | 2015 | Male | 66.54 | 66.71 |
| 473 | #5 | 15 | 2014 | Male | 66.62 | 58.85 |
| 474 | #5 | 74 | 2014 | Male | 66.61 | 67.24 |
| 475 | #5 | 54 | 2014 | Male | 66.25 | 64.99 |
| 476 | #5 | 74 | 2014 | Female | 66.95 | 70.61 |
| 477 | #5 | 48 | 2014 | Female | 66.87 | 65.08 |
| 478 | #5 | 25 | 2015 | Male | 67.86 | 55.22 |
| 479 | #5 | 44 | 2014 | Male | 66.00 | 63.89 |
| 480 | #5 | 47 | 2014 | Male | 62.13 | 53.25 |
| 481 | #5 | 49 | 2013 | Female | 63.52 | 64.86 |
| 482 | #5 | 73 | 2013 | Male | 64.96 | 71.86 |
| 483 | #5 | 58 | 2013 | Female | 68.27 | 70.46 |
| 484 | #5 | 69 | 2013 | Female | 63.75 | 67.30 |
| 485 | #5 | 2 | 2014 | Female | 64.25 | 51.98 |
| 486 | #5 | 9 | 2013 | Female | 67.33 | 53.46 |
| 487 | #5 | 27 | 2013 | Male | 65.91 | 63.46 |
| 488 | #5 | 67 | 2014 | Female | 68.32 | 66.72 |
| 489 | #5 | 3 | 2013 | Female | 67.43 | 53.99 |
| 490 | #22 | 5 | 2013 | Male | 65.01 | 55.20 |
| 491 | #22 | 21 | 2013 | Female | 63.99 | 59.15 |
| 492 | #22 | 27 | 2013 | Female | 68.71 | 61.63 |
| 493 | #22 | 69 | 2013 | Male | 68.14 | 72.19 |
| 494 | #22 | 72 | 2014 | Female | 66.84 | 58.50 |
| 495 | #22 | 34 | 2013 | Male | 65.75 | 64.79 |
| 496 | #22 | 52 | 2013 | Female | 68.23 | 68.24 |
| 497 | #22 | 43 | 2014 | Female | 65.19 | 60.58 |
| 498 | #22 | 15 | 2015 | Male | 68.55 | 56.50 |
| 499 | #22 | 45 | 2015 | Male | 65.57 | 66.80 |
| 500 | #22 | 18 | 2014 | Female | 64.18 | 56.46 |
| 501 | #22 | 74 | 2014 | Male | 68.15 | 65.53 |
| 502 | #22 | 68 | 2014 | Male | 68.24 | 65.23 |
| 503 | #22 | 38 | 2015 | Male | 69.28 | 60.31 |
| 504 | #22 | 74 | 2015 | Male | 68.03 | 69.29 |
| 505 | #22 | 73 | 2015 | Female | 71.36 | 71.96 |
| 506 | #22 | 59 | 2015 | Male | 84.15 | 63.41 |
| 507 | #22 | 39 | 2015 | Male | 71.37 | 65.64 |
| 508 | #22 | 58 | 2014 | Female | NA | NA |
| 509 | #22 | 71 | 2015 | Male | 69.40 | 72.58 |
| 510 | #22 | 63 | 2013 | Female | 71.99 | 77.69 |
| 511 | #22 | 62 | 2014 | Male | 66.74 | 61.72 |
| 512 | #22 | 30 | 2013 | Male | 63.97 | 58.49 |
| 513 | #22 | 67 | 2014 | Female | 68.94 | 69.17 |
| 514 | #22 | 50 | 2014 | Male | 65.31 | 66.14 |
| 515 | #22 | 54 | 2014 | Female | 65.97 | 67.97 |
| 516 | #22 | 62 | 2013 | Male | 69.15 | 79.57 |
| 517 | #22 | 68 | 2015 | Female | 71.92 | 72.04 |
| 518 | #22 | 70 | 2015 | Female | 68.59 | 70.18 |
| 519 | #22 | 27 | 2013 | Male | 65.49 | 61.32 |
| 520 | #22 | 27 | 2014 | Male | 63.22 | 59.75 |
| 521 | #22 | 44 | 2014 | Male | 66.05 | 66.62 |
| 522 | #22 | 69 | 2014 | Female | 66.22 | 60.25 |
| 523 | #22 | 67 | 2014 | Female | 68.18 | 67.08 |
| 524 | #22 | 57 | 2014 | Male | 66.84 | 66.44 |
| 525 | #22 | 69 | 2014 | Female | 67.45 | 70.00 |
| 526 | #22 | 5 | 2014 | Female | 67.15 | 58.18 |
| 527 | #22 | 44 | 2014 | Male | 67.94 | 69.23 |
| 528 | #22 | 60 | 2015 | Female | 69.22 | 67.70 |
| 529 | #22 | 54 | 2015 | Female | 68.96 | 67.50 |
| 530 | #22 | 60 | 2014 | Male | 68.74 | 71.28 |
| 531 | #22 | 54 | 2014 | Male | 68.66 | 71.56 |
| 532 | #22 | 68 | 2014 | Female | 66.55 | 67.61 |
| 533 | #12 | 59 | 2013 | Female | 66.91 | 65.71 |
| 534 | #12 | 21 | 2013 | Male | 68.27 | 70.32 |
| 535 | #12 | 68 | 2014 | Male | 66.33 | 67.16 |
| 536 | #12 | 35 | 2013 | Male | 63.74 | 64.17 |
| 537 | #12 | 65 | 2014 | Female | 67.54 | 67.22 |
| 538 | #12 | 26 | 2013 | Male | 63.75 | 61.30 |
| 539 | #12 | 57 | 2014 | Female | 66.92 | 65.79 |
| 540 | #12 | 40 | 2014 | Male | 67.07 | 64.37 |
| 541 | #12 | 2 | 2013 | Female | 67.33 | 53.46 |
| 542 | #12 | 59 | 2015 | Male | 68.70 | 71.98 |
| 543 | #12 | 43 | 2015 | Male | 70.55 | 68.95 |
| 544 | #12 | 19 | 2013 | Male | 65.91 | 61.46 |
| 545 | #12 | 10 | 2013 | Male | 67.43 | 59.99 |
| 546 | #12 | 3 | 2015 | Male | 65.98 | 53.14 |
| 547 | #12 | 45 | 2013 | Male | 63.52 | 64.86 |
| 548 | #12 | 46 | 2015 | Female | 66.19 | 67.40 |
| 549 | #12 | 14 | 2013 | Male | 66.34 | 59.38 |
| 550 | #12 | 67 | 2014 | Female | 67.65 | 69.00 |
| 551 | #12 | 61 | 2013 | Female | 67.33 | 69.46 |
| 552 | #12 | 47 | 2013 | Male | 64.96 | 70.86 |
| 553 | #12 | 61 | 2013 | Female | 65.91 | 69.46 |
| 554 | #12 | 72 | 2013 | Female | 67.43 | 69.99 |
| 555 | #12 | 71 | 2014 | Male | 66.73 | 75.66 |
| 556 | #12 | 6 | 2013 | Female | 66.58 | 50.28 |
| 557 | #12 | 74 | 2014 | Female | 67.68 | 66.30 |
| 558 | #12 | 64 | 2014 | Female | 66.23 | 65.88 |
| 559 | #12 | 63 | 2013 | Female | 66.34 | 67.38 |
| 560 | #12 | 17 | 2013 | Female | 67.88 | 60.36 |
| 561 | #12 | 61 | 2013 | Female | 66.58 | 66.28 |
| 562 | #12 | 12 | 2013 | Female | 67.42 | 61.64 |
| 563 | #12 | 12 | 2013 | Male | 63.73 | 57.95 |
| 564 | #12 | 47 | 2015 | Male | 70.33 | 65.70 |
| 565 | #12 | 22 | 2014 | Female | 66.78 | 63.98 |
| 566 | #12 | 57 | 2014 | Female | 66.41 | 68.12 |
| 567 | #12 | 45 | 2013 | Male | 68.27 | 69.46 |
| 568 | #12 | 19 | 2014 | Female | 66.54 | 60.21 |
| 569 | #12 | 69 | 2013 | Male | 67.88 | 70.36 |
| 570 | #12 | 24 | 2013 | Female | 65.28 | 65.33 |
| 571 | #12 | 53 | 2014 | Male | 66.88 | 69.29 |
| 572 | #12 | 46 | 2014 | Male | 68.01 | 60.74 |
| 573 | #12 | 69 | 2013 | Male | 67.42 | 67.14 |
| 574 | #12 | 39 | 2014 | Female | 67.52 | 63.12 |
| 575 | #12 | 54 | 2014 | Female | 66.13 | 67.53 |
| 576 | #12 | 24 | 2014 | Male | 66.32 | 61.55 |
| 577 | #12 | 71 | 2015 | Male | 68.44 | 70.32 |
| 578 | #12 | 48 | 2013 | Male | 63.75 | 66.30 |
| 579 | #12 | 73 | 2013 | Female | 63.73 | 65.95 |
| 580 | #12 | 54 | 2014 | Female | 66.43 | 63.53 |
| 581 | #12 | 74 | 2015 | Female | 68.83 | 70.22 |
| 582 | #20 | 63 | 2013 | Male | 64.02 | 55.00 |
| 583 | #20 | 41 | 2013 | Male | 64.13 | 56.52 |
| 584 | #20 | 44 | 2014 | Female | 66.56 | 66.30 |
| 585 | #20 | 28 | 2015 | Female | 68.62 | 62.24 |
| 586 | #20 | 40 | 2014 | Female | 66.52 | 65.17 |
| 587 | #20 | 25 | 2014 | Female | 67.05 | 61.12 |
| 588 | #20 | 60 | 2014 | Male | 66.54 | 60.22 |
| 589 | #20 | 61 | 2014 | Female | 66.30 | 68.46 |
| 590 | #20 | 19 | 2014 | Male | 66.32 | 54.99 |
| 591 | #20 | 51 | 2014 | Male | 67.43 | 64.59 |
| 592 | #20 | 49 | 2015 | Male | 67.88 | 62.42 |
| 593 | #20 | 51 | 2014 | Female | 66.82 | 64.40 |
| 594 | #20 | 26 | 2014 | Female | 64.47 | 56.19 |
| 595 | #20 | 39 | 2014 | Female | 65.45 | 67.09 |
| 596 | #20 | 46 | 2014 | Male | 66.97 | 64.97 |
| 597 | #20 | 46 | 2014 | Male | 65.54 | 58.22 |
| 598 | #20 | 52 | 2015 | Female | 69.08 | 64.88 |
| 599 | #20 | 40 | 2014 | Female | 66.72 | 63.93 |
| 600 | #20 | 74 | 2015 | Male | 63.28 | 66.76 |
| 601 | #20 | 2 | 2013 | Female | 66.14 | 52.51 |
| 602 | #20 | 20 | 2013 | Male | 66.86 | 55.56 |
| 603 | #20 | 42 | 2015 | Female | 68.10 | 68.97 |
| 604 | #20 | 7 | 2013 | Female | 63.21 | 45.82 |
| 605 | #20 | 39 | 2014 | Male | 67.81 | 67.84 |
| 606 | #20 | 63 | 2013 | Female | 65.56 | 60.81 |
| 607 | #20 | 47 | 2014 | Female | 66.21 | 87.97 |
| 608 | #20 | 58 | 2013 | Male | 69.33 | 68.32 |
| 609 | #20 | 33 | 2014 | Female | 66.02 | 59.06 |
| 610 | #20 | 30 | 2014 | Female | 64.58 | 56.96 |
| 611 | #20 | 17 | 2013 | Male | 65.98 | 58.00 |
| 612 | #20 | 3 | 2013 | Female | 64.72 | 47.40 |
| 613 | #20 | 47 | 2013 | Female | 66.43 | 70.85 |
| 614 | #20 | 24 | 2013 | Male | 67.11 | 62.88 |
| 615 | #20 | 55 | 2014 | Female | 64.33 | 64.70 |
| 616 | #20 | 40 | 2014 | Male | 68.08 | 62.75 |
| 617 | #20 | 62 | 2014 | Male | 64.38 | 70.83 |
| 618 | #9 | 46 | 2015 | Male | 68.75 | 67.91 |
| 619 | #9 | 50 | 2015 | Female | 62.34 | 71.85 |
| 620 | #9 | 15 | 2015 | Female | 67.17 | 55.24 |
| 621 | #9 | 50 | 2015 | Male | 68.38 | 71.97 |
| 622 | #9 | 3 | 2015 | Female | 69.19 | 52.76 |
| 623 | #9 | 49 | 2015 | Female | 63.02 | 66.97 |
| 624 | #9 | 31 | 2015 | Female | 72.90 | 67.88 |
| 625 | #9 | 63 | 2015 | Female | 73.06 | 67.66 |
| 626 | #9 | 74 | 2015 | Female | 69.39 | 70.88 |
| 627 | #9 | 11 | 2015 | Female | 68.60 | 54.73 |
| 628 | #9 | 63 | 2015 | Female | 68.74 | 74.49 |
| 629 | #9 | 38 | 2015 | Female | 69.60 | 64.94 |
| 630 | #9 | 61 | 2015 | Female | NA | NA |
| 631 | #9 | 61 | 2014 | Female | 68.08 | 71.17 |
| 632 | #9 | 46 | 2015 | Male | 69.09 | 69.04 |
| 633 | #9 | 56 | 2015 | Female | 65.85 | 65.44 |
| 634 | #9 | 46 | 2014 | Male | 66.11 | 66.34 |
| 635 | #9 | 17 | 2015 | Male | 69.37 | 56.35 |
| 636 | #9 | 43 | 2014 | Female | 67.37 | 66.41 |
| 637 | #9 | 43 | 2015 | Male | 73.75 | 71.11 |
| 638 | #9 | 25 | 2015 | Female | 64.97 | 56.73 |
| 639 | #9 | 1 | 2014 | Male | 66.12 | 57.16 |
| 640 | #9 | 44 | 2014 | Male | 67.65 | 67.67 |
| 641 | #9 | 9 | 2014 | Male | 66.62 | 60.10 |
| 642 | #9 | 29 | 2014 | Female | 67.15 | 63.77 |
| 643 | #9 | 71 | 2015 | Female | 68.97 | 73.42 |
| 644 | #9 | 18 | 2015 | Female | 69.87 | 62.44 |
| 645 | #21 | 72 | 2014 | Male | 67.21 | 66.23 |
| 646 | #21 | 53 | 2015 | Male | 68.13 | 69.38 |
| 647 | #21 | 58 | 2014 | Female | 66.32 | 68.55 |
| 648 | #21 | 53 | 2014 | Male | 67.43 | 64.78 |
| 649 | #21 | 74 | 2014 | Male | 67.29 | 54.13 |
| 650 | #21 | 70 | 2015 | Female | 66.03 | 70.94 |
| 651 | #21 | 72 | 2014 | Male | 67.76 | 66.25 |
| 652 | #21 | 44 | 2013 | Female | 66.58 | 67.28 |
| 653 | #21 | 54 | 2014 | Male | 67.15 | 68.83 |
| 654 | #21 | 23 | 2013 | Female | 67.88 | 60.36 |
| 655 | #21 | 68 | 2015 | Female | 65.01 | 61.37 |
| 656 | #21 | 73 | 2015 | Female | 65.55 | 71.04 |
| 657 | #21 | 41 | 2013 | Female | 67.42 | 70.64 |
| 658 | #21 | 21 | 2013 | Male | 63.73 | 69.95 |
| 659 | #21 | 71 | 2014 | Female | 66.61 | 68.68 |
| 660 | #21 | 69 | 2013 | Female | 65.28 | 77.33 |
| 661 | #21 | 74 | 2014 | Male | 66.87 | 66.96 |
| 662 | #21 | 6 | 2013 | Male | 66.91 | 51.71 |
| 663 | #21 | 66 | 2013 | Female | 63.74 | 63.17 |
| 664 | #21 | 48 | 2014 | Male | 67.32 | 60.22 |
| 665 | #21 | 68 | 2015 | Female | 66.37 | 63.90 |
| 666 | #21 | 66 | 2013 | Male | 63.52 | 71.86 |
| 667 | #21 | 72 | 2013 | Female | 64.96 | 71.86 |
| 668 | #21 | 26 | 2013 | Male | 68.27 | 60.46 |
| 669 | #21 | 43 | 2014 | Female | 68.08 | 59.14 |
| 670 | #21 | 27 | 2013 | Male | 63.75 | 57.82 |
| 671 | #21 | 46 | 2014 | Female | 67.41 | 65.57 |
| 672 | #13 | 74 | 2014 | Female | 68.51 | 68.21 |
| 673 | #13 | 55 | 2013 | Male | 67.42 | 71.64 |
| 674 | #13 | 47 | 2013 | Male | 63.73 | 64.95 |
| 675 | #13 | 25 | 2014 | Female | 66.13 | 61.59 |
| 676 | #13 | 41 | 2013 | Male | 65.28 | 69.33 |
| 677 | #13 | 63 | 2015 | Female | 68.01 | 67.68 |
| 678 | #13 | 73 | 2013 | Male | 63.73 | 65.95 |
| 679 | #13 | 49 | 2013 | Male | 66.91 | 64.71 |
| 680 | #13 | 72 | 2014 | Female | 67.61 | 69.54 |
| 681 | #13 | 64 | 2014 | Male | 68.34 | 69.55 |
| 682 | #13 | 61 | 2014 | Female | 67.14 | 69.53 |
| 683 | #13 | 72 | 2013 | Male | 65.28 | 70.33 |
| 684 | #13 | 52 | 2013 | Male | 63.74 | 67.17 |
| 685 | #13 | 65 | 2013 | Male | 66.91 | 65.71 |
| 686 | #13 | 57 | 2014 | Male | 69.97 | 70.97 |
| 687 | #13 | 68 | 2014 | Female | 67.82 | 70.94 |
| 688 | #13 | 21 | 2013 | Female | 66.58 | 61.28 |
| 689 | #13 | 74 | 2014 | Male | 70.71 | 66.61 |
| 690 | #13 | 65 | 2013 | Female | 63.74 | 67.17 |
| 691 | #13 | 48 | 2014 | Female | 67.48 | 69.95 |
| 692 | #13 | 50 | 2014 | Male | 64.71 | 64.63 |
| 693 | #13 | 49 | 2013 | Female | 67.33 | 68.46 |
| 694 | #13 | 63 | 2013 | Female | 63.52 | 65.86 |
| 695 | #15 | 6 | 2014 | Male | 64.95 | 55.55 |
| 696 | #15 | 50 | 2014 | Male | 64.83 | 64.01 |
| 697 | #15 | 41 | 2013 | Male | 67.91 | 67.34 |
| 698 | #15 | 69 | 2013 | Male | 66.53 | 65.96 |
| 699 | #15 | 41 | 2013 | Male | 64.55 | 66.99 |
| 700 | #15 | 39 | 2014 | Male | 68.92 | 66.25 |
| 701 | #15 | 59 | 2013 | Female | 64.13 | 68.54 |
| 702 | #15 | 24 | 2013 | Male | 65.72 | 63.56 |
| 703 | #15 | 52 | 2013 | Female | 65.43 | 64.84 |
| 704 | #15 | 19 | 2014 | Female | 65.49 | 57.79 |
| 705 | #15 | 45 | 2015 | Male | 64.12 | 66.17 |
| 706 | #15 | 58 | 2014 | Female | 61.05 | 54.33 |
| 707 | #15 | 25 | 2015 | Male | 67.81 | 63.08 |
| 708 | #15 | 47 | 2013 | Female | 67.19 | 64.30 |
| 709 | #15 | 40 | 2013 | Female | 68.71 | 67.73 |
| 710 | #15 | 60 | 2014 | Female | 67.30 | 68.84 |
| 711 | #15 | 47 | 2014 | Male | 66.53 | 66.62 |
| 712 | #15 | 28 | 2013 | Male | 68.12 | 65.93 |
| 713 | #15 | 53 | 2014 | Female | 65.94 | 70.27 |
| 714 | #15 | 12 | 2013 | Female | 64.11 | 55.60 |
| 715 | #15 | 58 | 2014 | Female | 64.58 | 69.37 |
| 716 | #15 | 6 | 2013 | Female | 63.19 | 52.04 |
| 717 | #15 | 19 | 2014 | Female | 64.97 | 57.54 |
| 718 | #15 | 28 | 2015 | Female | 66.89 | 63.57 |
| 719 | #15 | 39 | 2014 | Male | 64.86 | 65.81 |
| 720 | #15 | 29 | 2014 | Male | 63.19 | 60.68 |
| 721 | #15 | 61 | 2014 | Female | 69.22 | 70.90 |
| 722 | #15 | 27 | 2014 | Female | 65.58 | 65.26 |
| 723 | #15 | 71 | 2014 | Female | 66.12 | 64.10 |
| 724 | #15 | 53 | 2014 | Male | 67.31 | 69.26 |
| 725 | #15 | 16 | 2014 | Male | 66.03 | 58.52 |
| 726 | #15 | 25 | 2014 | Female | 67.51 | 65.63 |
| 727 | #10 | 26 | 2014 | Male | 69.14 | 65.73 |
| 728 | #10 | 23 | 2013 | Female | 67.32 | 60.74 |
| 729 | #10 | 60 | 2014 | Male | 66.88 | 69.81 |
| 730 | #10 | 54 | 2014 | Female | 67.48 | 65.56 |
| 731 | #10 | 30 | 2013 | Female | 66.18 | 62.54 |
| 732 | #10 | 34 | 2014 | Male | 68.00 | 66.81 |
| 733 | #10 | 62 | 2014 | Female | 67.63 | 67.23 |
| 734 | #10 | 46 | 2014 | Male | 67.11 | 67.91 |
| 735 | #10 | 52 | 2013 | Female | 65.33 | 68.43 |
| 736 | #10 | 25 | 2015 | Female | 68.75 | 70.38 |
| 737 | #10 | 39 | 2014 | Male | 66.71 | 63.70 |
| 738 | #10 | 18 | 2015 | Male | 65.15 | 62.07 |
| 739 | #10 | 69 | 2013 | Male | 69.01 | 80.11 |
| 740 | #10 | 73 | 2013 | Female | 67.12 | 74.94 |
| 741 | #10 | 39 | 2015 | Male | 64.13 | 62.74 |
| 742 | #10 | 73 | 2015 | Male | 67.14 | 65.37 |
| 743 | #10 | 60 | 2015 | Female | 66.35 | 63.93 |
| 744 | #10 | 52 | 2015 | Female | 64.03 | 66.18 |
| 745 | #10 | 29 | 2015 | Male | 64.87 | 60.39 |
| 746 | #10 | 24 | 2015 | Male | 65.13 | 62.39 |
| 747 | #10 | 4 | 2015 | Male | 66.31 | 60.58 |
| 748 | #10 | 45 | 2015 | Male | 65.13 | 65.27 |
| 749 | #10 | 29 | 2015 | Male | 68.79 | 62.69 |
| 750 | #10 | 72 | 2013 | Male | 67.38 | 72.80 |
| 751 | #10 | 29 | 2014 | Male | 65.38 | 57.16 |
| 752 | #10 | 36 | 2014 | Male | 67.26 | 63.39 |
| 753 | #10 | 72 | 2014 | Female | 66.49 | 69.16 |
| 754 | #10 | 19 | 2013 | Female | 66.98 | 56.34 |
| 755 | #10 | 58 | 2013 | Female | 66.38 | 67.13 |
| 756 | #10 | 73 | 2013 | Female | 66.54 | 67.00 |
| 757 | #10 | 31 | 2015 | Female | 65.44 | 63.58 |
| 758 | #10 | 69 | 2013 | Male | 63.19 | 74.89 |
| 759 | #10 | 37 | 2014 | Male | 67.14 | 63.95 |
| 760 | #14 | 7 | 2013 | Male | 66.40 | 57.14 |
| 761 | #14 | 67 | 2014 | Male | 66.44 | 66.88 |
| 762 | #14 | 7 | 2013 | Female | 67.81 | 47.40 |
| 763 | #14 | 54 | 2014 | Female | 67.58 | 67.07 |
| 764 | #14 | 31 | 2013 | Male | 67.18 | 62.91 |
| 765 | #14 | 33 | 2013 | Male | 66.67 | 63.60 |
| 766 | #14 | 38 | 2013 | Female | 66.32 | 66.13 |
| 767 | #14 | 48 | 2013 | Female | 66.52 | 67.86 |
| 768 | #14 | 10 | 2015 | Male | 64.13 | 58.43 |
| 769 | #14 | 67 | 2014 | Female | 66.28 | 67.48 |
| 770 | #14 | 67 | 2014 | Female | 67.48 | 70.49 |
| 771 | #14 | 61 | 2013 | Male | 65.30 | 60.73 |
| 772 | #14 | 69 | 2013 | Female | 66.21 | 65.72 |
| 773 | #14 | 53 | 2014 | Female | 63.99 | 63.64 |
| 774 | #14 | 12 | 2014 | Female | 66.54 | 52.80 |
| 775 | #14 | 26 | 2014 | Female | 66.87 | 61.35 |
| 776 | #14 | 46 | 2014 | Female | 66.21 | 66.12 |
| 777 | #14 | 60 | 2014 | Male | 67.73 | 70.06 |
| 778 | #14 | 44 | 2014 | Male | 66.29 | 67.47 |
| 779 | #14 | 61 | 2014 | Female | 65.13 | 61.25 |
| 780 | #14 | 53 | 2014 | Female | 65.01 | 66.02 |
| 781 | #14 | 55 | 2014 | Female | 63.21 | 68.91 |
| 782 | #14 | 72 | 2014 | Male | 66.76 | 65.76 |
| 783 | #14 | 74 | 2014 | Female | 66.45 | 67.00 |
| 784 | #14 | 59 | 2014 | Female | 67.07 | 67.47 |
| 785 | #14 | 39 | 2014 | Male | 66.41 | 64.55 |
| 786 | #14 | 62 | 2014 | Female | 65.67 | 68.55 |
| 787 | #14 | 24 | 2013 | Male | 67.26 | 56.90 |
| 788 | #14 | 44 | 2014 | Male | 67.39 | 68.66 |
| 789 | #14 | 39 | 2014 | Male | 67.51 | 64.48 |
| 790 | #14 | 50 | 2014 | Male | 65.84 | 62.70 |
| 791 | #14 | 27 | 2013 | Female | 66.88 | 61.78 |
| 792 | #14 | 41 | 2013 | Male | 67.31 | 63.98 |
| 793 | #14 | 71 | 2014 | Male | 68.83 | 73.16 |
| 794 | #14 | 7 | 2013 | Female | 66.49 | 50.69 |
| 795 | #14 | 44 | 2014 | Female | 65.24 | 60.07 |
| 796 | #14 | 47 | 2013 | Male | 65.10 | 68.46 |
| 797 | #14 | 44 | 2013 | Female | 67.89 | 65.62 |
| 798 | #14 | 72 | 2013 | Female | 66.43 | 69.44 |
| 799 | #14 | 48 | 2014 | Female | 65.13 | 62.93 |
| 800 | #14 | 54 | 2013 | Female | 67.57 | 67.36 |
| 801 | #14 | 70 | 2013 | Female | 67.46 | 61.86 |
| 802 | #14 | 40 | 2015 | Male | 66.97 | 62.61 |
| 803 | #14 | 43 | 2014 | Male | 64.07 | 56.59 |
| 804 | #14 | 72 | 2014 | Female | 64.16 | 61.86 |
| 805 | #14 | 58 | 2014 | Female | 65.28 | 69.48 |
| 806 | #14 | 55 | 2013 | Male | 66.91 | 64.55 |
| 807 | #14 | 68 | 2014 | Male | 66.47 | 67.29 |
| 808 | #14 | 68 | 2014 | Male | 66.53 | 66.71 |
| 809 | #14 | 21 | 2013 | Female | 68.04 | 60.73 |
| 810 | #14 | 51 | 2014 | Male | 67.49 | 68.13 |
| 811 | #14 | 34 | 2013 | Male | 66.56 | 75.43 |
| 812 | #14 | 74 | 2014 | Female | 65.12 | 69.69 |
| 813 | #14 | 73 | 2013 | Male | 67.52 | 67.53 |
| 814 | #14 | 62 | 2013 | Female | 67.17 | 67.79 |
| 815 | #14 | 62 | 2013 | Male | 66.02 | 75.37 |
| 816 | #14 | 48 | 2013 | Female | 68.52 | 72.60 |
| 817 | #16 | 72 | 2013 | Female | 64.05 | 64.09 |
| 818 | #16 | 69 | 2014 | Female | 64.66 | 71.37 |
| 819 | #16 | 54 | 2013 | Female | 70.15 | 69.03 |
| 820 | #16 | 37 | 2014 | Male | 68.78 | 67.95 |
| 821 | #16 | 59 | 2015 | Female | 65.13 | 69.30 |
| 822 | #16 | 58 | 2014 | Male | 69.90 | 74.43 |
| 823 | #16 | 46 | 2014 | Female | 65.88 | 65.98 |
| 824 | #16 | 61 | 2013 | Male | 66.15 | 65.01 |
| 825 | #16 | 74 | 2014 | Male | 66.66 | 72.09 |
| 826 | #16 | 72 | 2013 | Female | 66.94 | 70.90 |
| 827 | #16 | 62 | 2013 | Female | 67.48 | 67.80 |
| 828 | #16 | 72 | 2013 | Female | 66.94 | 74.34 |
| 829 | #16 | 60 | 2014 | Male | 68.83 | 67.88 |
| 830 | #16 | 47 | 2014 | Male | 67.99 | 67.84 |
| 831 | #16 | 52 | 2015 | Female | 67.05 | 66.52 |
| 832 | #16 | 34 | 2013 | Female | 67.48 | 66.64 |
| 833 | #16 | 68 | 2013 | Female | 63.18 | 66.70 |
| 834 | #16 | 73 | 2013 | Female | 63.18 | 68.11 |
| 835 | #16 | 58 | 2013 | Female | 66.37 | 65.68 |
| 836 | #16 | 47 | 2013 | Female | 69.15 | 70.14 |
| 837 | #16 | 46 | 2014 | Male | 68.36 | 62.32 |
| 838 | #16 | 72 | 2013 | Female | 65.89 | 68.62 |
| 839 | #16 | 47 | 2014 | Female | 66.01 | 70.89 |
| 840 | #16 | 53 | 2015 | Female | 68.72 | 75.33 |
| 841 | #16 | 73 | 2013 | Female | 64.19 | 66.97 |
| 842 | #16 | 73 | 2013 | Male | 64.56 | 68.68 |
| 843 | #16 | 72 | 2013 | Female | 63.91 | 67.11 |
| 844 | #16 | 74 | 2014 | Male | 69.84 | 70.56 |
| 845 | #16 | 45 | 2013 | Male | 68.15 | 69.84 |
| 846 | #16 | 55 | 2013 | Female | 66.34 | 73.69 |
| 847 | #16 | 68 | 2013 | Male | 64.52 | 64.66 |
| 848 | #16 | 51 | 2013 | Female | 62.01 | 57.86 |
| 849 | #16 | 74 | 2014 | Female | 69.33 | 69.68 |
| 850 | #16 | 52 | 2013 | Male | 63.25 | 66.33 |
| 851 | #16 | 52 | 2013 | Female | 63.27 | 64.88 |
| 852 | #16 | 54 | 2013 | Male | 65.29 | 68.87 |
| 853 | #16 | 54 | 2013 | Female | 62.87 | 63.28 |
| 854 | #16 | 54 | 2013 | Female | 64.13 | 64.06 |
| 855 | #16 | 68 | 2013 | Female | 64.36 | 66.78 |
| 856 | #16 | 58 | 2013 | Male | 64.91 | 68.63 |
| 857 | #16 | 74 | 2014 | Male | 64.75 | 73.66 |
| 858 | #16 | 65 | 2013 | Female | 70.12 | 76.19 |
| 859 | #16 | 74 | 2014 | Female | 64.36 | 68.58 |
| 860 | #16 | 67 | 2014 | Female | 65.62 | 67.37 |
| 861 | #16 | 67 | 2014 | Male | 67.74 | 69.64 |
| 862 | #16 | 71 | 2014 | Male | 65.40 | 71.64 |
| 863 | #16 | 64 | 2014 | Female | 64.19 | 66.39 |
| 864 | #16 | 53 | 2014 | Male | 68.05 | 71.01 |
| 865 | #16 | 40 | 2014 | Female | 65.32 | 69.88 |
| 866 | #16 | 74 | 2014 | Male | 65.04 | 65.82 |
| 867 | #16 | 47 | 2014 | Female | 62.37 | 65.76 |
| 868 | #1 | 28 | 2013 | Female | 66.58 | 55.28 |
| 869 | #1 | 52 | 2013 | Female | 67.42 | 71.64 |
| 870 | #1 | 61 | 2014 | Female | 66.36 | 67.61 |
| 871 | #1 | 46 | 2014 | Male | 67.21 | 66.32 |
| 872 | #1 | 33 | 2014 | Female | 67.22 | 67.11 |
| 873 | #1 | 33 | 2015 | Male | 69.06 | 63.27 |
| 874 | #1 | 24 | 2015 | Female | 70.73 | 61.54 |
| 875 | #1 | 33 | 2015 | Female | 64.54 | 61.52 |
| 876 | #1 | 47 | 2013 | Female | 63.73 | 64.95 |
| 877 | #1 | 51 | 2013 | Female | 65.28 | 70.33 |
| 878 | #1 | 4 | 2015 | Female | 66.79 | 50.77 |
| 879 | #1 | 64 | 2014 | Male | 67.80 | 66.83 |
| 880 | #1 | 16 | 2014 | Female | 70.18 | 63.40 |
| 881 | #1 | 55 | 2014 | Female | 67.76 | 69.45 |
| 882 | #1 | 72 | 2014 | Male | 67.64 | 67.37 |
| 883 | #1 | 72 | 2013 | Female | 68.05 | 69.95 |
| 884 | #1 | 46 | 2014 | Male | 69.71 | 74.70 |
| 885 | #1 | 60 | 2014 | Female | 68.12 | 70.01 |
| 886 | #1 | 10 | 2015 | Male | 67.15 | 53.26 |
| 887 | #1 | 62 | 2013 | Female | 66.01 | 72.14 |
| 888 | #1 | 39 | 2014 | Female | 66.45 | 70.50 |
| 889 | #1 | 57 | 2014 | Male | 66.34 | 67.20 |
| 890 | #1 | 49 | 2013 | Female | 66.91 | 64.71 |
| 891 | #1 | 24 | 2013 | Male | 67.88 | 64.36 |
| 892 | #1 | 47 | 2013 | Male | 66.89 | 75.30 |
| 893 | #1 | 72 | 2013 | Female | 67.28 | 67.11 |
| 894 | #1 | 44 | 2013 | Male | 63.52 | 64.86 |
| 895 | #1 | 26 | 2014 | Female | 67.82 | 61.21 |
| 896 | #11 | 12 | 2014 | Male | 66.67 | 59.24 |
| 897 | #11 | 27 | 2014 | Female | 66.71 | 62.68 |
| 898 | #11 | 54 | 2014 | Female | 67.31 | 70.87 |
| 899 | #11 | 41 | 2014 | Female | 67.02 | 69.65 |
| 900 | #11 | 57 | 2014 | Female | 66.55 | 71.16 |
| 901 | #11 | 53 | 2014 | Female | 66.74 | 67.59 |
| 902 | #11 | 11 | 2014 | Female | 66.98 | 56.67 |
| 903 | #11 | 18 | 2014 | Female | 67.56 | 55.11 |
| 904 | #11 | 67 | 2014 | Female | 67.55 | 68.13 |
| 905 | #11 | 74 | 2014 | Male | 67.39 | 68.61 |
| 906 | #11 | 46 | 2014 | Female | 68.46 | 70.65 |
| 907 | #11 | 25 | 2014 | Male | 67.23 | 61.39 |
| 908 | #11 | 20 | 2014 | Female | 66.19 | 57.17 |
| 909 | #11 | 46 | 2014 | Male | 67.52 | 68.11 |
| 910 | #11 | 54 | 2014 | Female | 68.39 | 73.66 |
| 911 | #11 | 19 | 2014 | Male | 66.54 | 60.48 |
| 912 | #11 | 5 | 2014 | Male | 66.32 | 57.13 |
| 913 | #11 | 23 | 2014 | Female | 67.52 | 64.26 |
| 914 | #11 | 32 | 2014 | Female | 66.41 | 65.06 |
| 915 | #11 | 68 | 2013 | Male | 67.88 | 69.24 |
| 916 | #11 | 29 | 2015 | Female | 64.86 | 53.81 |
| 917 | #11 | 26 | 2014 | Female | 68.05 | 61.87 |
| 918 | #11 | 36 | 2014 | Female | 66.94 | 64.68 |
| 919 | #11 | 60 | 2014 | Female | 66.19 | 70.96 |
| 920 | #11 | 7 | 2013 | Female | 65.12 | 44.84 |
| 921 | #11 | 6 | 2013 | Male | 67.67 | 60.65 |
| 922 | #11 | 56 | 2013 | Male | 70.99 | 72.78 |
| 923 | #11 | 27 | 2013 | Female | 67.90 | 61.69 |
| 924 | #11 | 27 | 2013 | Male | 66.85 | 66.09 |
| 925 | #11 | 22 | 2014 | Female | 67.61 | 58.41 |
| 926 | #11 | 37 | 2013 | Male | 66.60 | 67.18 |
| 927 | #11 | 38 | 2013 | Male | 68.56 | 70.17 |
| 928 | #11 | 67 | 2015 | Male | NA | NA |
| 929 | #11 | 17 | 2013 | Male | 67.31 | 60.17 |
| 930 | #11 | 41 | 2014 | Female | 65.49 | 65.65 |
| 931 | #11 | 29 | 2014 | Male | 67.33 | 63.49 |
| 932 | #11 | 7 | 2013 | Male | 64.74 | 58.73 |
| 933 | #11 | 5 | 2013 | Female | 67.33 | 53.18 |
| 934 | #11 | 20 | 2013 | Male | 68.91 | 66.10 |
| 935 | #11 | 51 | 2013 | Female | 66.54 | 74.26 |
| 936 | #8 | 40 | 2013 | Female | 63.52 | 63.86 |
| 937 | #8 | 62 | 2014 | Female | 65.84 | 68.51 |
| 938 | #8 | 61 | 2014 | Female | 66.42 | 67.53 |
| 939 | #8 | 68 | 2014 | Male | 68.88 | 72.14 |
| 940 | #8 | 73 | 2013 | Female | 67.88 | 70.95 |
| 941 | #8 | 69 | 2014 | Male | 68.02 | 66.25 |
| 942 | #8 | 64 | 2014 | Male | 70.48 | 74.60 |
| 943 | #8 | 12 | 2013 | Male | 66.91 | 62.71 |
| 944 | #8 | 68 | 2014 | Female | 65.12 | 68.82 |
| 945 | #8 | 68 | 2013 | Female | 67.58 | 66.39 |
| 946 | #8 | 40 | 2013 | Female | 64.96 | 69.86 |
| 947 | #8 | 69 | 2013 | Female | 67.56 | 72.72 |
| 948 | #8 | 33 | 2013 | Female | 67.81 | 69.10 |
| 949 | #8 | 47 | 2013 | Female | 66.02 | 63.40 |
| 950 | #8 | 72 | 2013 | Female | 69.03 | 71.15 |
| 951 | #19 | 32 | 2015 | Female | 70.51 | 76.62 |
| 952 | #19 | 72 | 2014 | Male | 67.87 | 68.21 |
| 953 | #19 | 53 | 2014 | Female | 66.13 | 63.30 |
| 954 | #19 | 18 | 2015 | Male | 69.12 | 62.96 |
| 955 | #19 | 46 | 2014 | Female | 65.83 | 62.71 |
| 956 | #19 | 48 | 2013 | Female | 66.54 | 76.79 |
| 957 | #19 | 41 | 2014 | Female | 67.96 | 66.69 |
| 958 | #19 | 69 | 2014 | Female | NA | NA |
| 959 | #19 | 20 | 2013 | Female | 67.09 | 63.33 |
| 960 | #19 | 5 | 2013 | Male | 66.65 | 67.78 |
| 961 | #19 | 41 | 2013 | Male | 65.48 | 67.03 |
| 962 | #19 | 20 | 2013 | Male | 67.31 | 67.86 |
| 963 | #19 | 9 | 2014 | Male | 64.02 | 57.15 |
| 964 | #19 | 47 | 2014 | Female | 66.96 | 67.48 |
| 965 | #19 | 27 | 2013 | Female | 66.36 | 69.29 |
| 966 | #19 | 9 | 2013 | Female | 65.43 | 54.54 |
| 967 | #19 | 9 | 2013 | Male | 65.21 | 58.22 |
| 968 | #19 | 17 | 2015 | Female | 64.55 | 62.59 |
| 969 | #19 | 12 | 2013 | Male | 67.84 | 57.37 |
| 970 | #19 | 39 | 2015 | Female | 66.37 | 71.79 |
| 971 | #17 | 71 | 2014 | Female | 65.49 | 64.38 |
| 972 | #17 | 73 | 2015 | Male | 64.17 | 68.34 |
| 973 | #17 | 48 | 2014 | Female | 66.14 | 63.70 |
| 974 | #17 | 47 | 2014 | Female | 66.94 | 64.64 |
| 975 | #17 | 54 | 2014 | Female | 67.01 | 63.21 |
| 976 | #17 | 47 | 2014 | Female | 66.59 | 63.58 |
| 977 | #17 | 28 | 2015 | Female | 64.28 | 57.69 |
| 978 | #17 | 59 | 2015 | Male | 66.12 | 71.69 |
| 979 | #17 | 71 | 2015 | Female | 63.97 | 65.17 |
| 980 | #17 | 40 | 2014 | Male | 66.31 | 63.27 |
| 981 | #17 | 68 | 2014 | Male | 65.13 | 71.02 |
| 982 | #17 | 17 | 2015 | Female | 63.97 | 53.48 |
| 983 | #17 | 73 | 2015 | Female | 64.55 | 64.42 |
| 984 | #17 | 22 | 2015 | Female | 65.03 | 61.05 |
| 985 | #17 | 39 | 2015 | Female | 65.14 | 65.83 |
| 986 | #17 | 53 | 2014 | Female | 68.94 | 71.28 |
| 987 | #1 | 39 | 2014 | Female | 66.13 | 68.40 |
| 988 | #1 | 11 | 2015 | Male | 70.40 | 59.16 |
| 989 | #1 | 37 | 2014 | Male | 66.86 | 64.40 |
| 990 | #1 | 13 | 2014 | Female | 67.43 | 58.79 |
| 991 | #1 | 50 | 2014 | Male | 67.64 | 69.79 |
| 992 | #1 | 42 | 2015 | Male | 69.30 | 66.84 |
| 993 | #1 | 36 | 2014 | Male | 67.76 | 61.97 |
| 994 | #1 | 54 | 2014 | Female | 67.86 | 68.71 |
| 995 | #1 | 5 | 2015 | Female | 66.12 | 49.28 |
| 996 | #1 | 53 | 2014 | Female | 65.79 | 67.79 |
| 997 | #1 | 32 | 2014 | Female | 67.27 | 68.32 |
| 998 | #1 | 67 | 2014 | Female | 67.54 | 70.22 |
| 999 | #1 | 35 | 2015 | Male | 73.27 | 71.24 |
| 1000 | #1 | 11 | 2014 | Male | 67.98 | 60.82 |
| 1001 | #1 | 37 | 2014 | Male | 67.65 | 62.92 |
| 1002 | #1 | 46 | 2014 | Female | 67.83 | 70.20 |
| 1003 | #1 | 50 | 2014 | Male | 66.21 | 69.40 |
| 1004 | #1 | 29 | 2014 | Male | 67.32 | 62.43 |
| 1005 | #1 | 29 | 2014 | Male | 66.51 | 61.80 |
| 1006 | #1 | 43 | 2014 | Male | 66.42 | 65.85 |
| 1007 | #1 | 43 | 2014 | Female | 66.55 | 66.09 |
| 1008 | #1 | 71 | 2014 | Female | 65.77 | 73.55 |
| 1009 | #1 | 25 | 2014 | Female | 65.99 | 64.58 |
| 1010 | #1 | 30 | 2014 | Male | 68.32 | 60.40 |
| 1011 | #1 | 68 | 2014 | Male | 67.89 | 70.52 |
| 1012 | #1 | 38 | 2015 | Male | 68.05 | 65.35 |
| 1013 | #1 | 67 | 2014 | Male | 68.18 | 69.68 |
| 1014 | #13 | 41 | 2014 | Male | 72.67 | 68.56 |
| 1015 | #13 | 47 | 2014 | Female | 69.44 | 68.10 |
| 1016 | #13 | 71 | 2014 | Female | 68.32 | 64.71 |
| 1017 | #13 | 50 | 2014 | Female | 68.76 | 67.83 |
| 1018 | #13 | 68 | 2014 | Female | 68.43 | 69.41 |
| 1019 | #13 | 55 | 2014 | Male | 68.35 | 69.77 |
| 1020 | #13 | 26 | 2014 | Male | 80.72 | 53.61 |
| 1021 | #13 | 23 | 2014 | Male | 65.42 | 59.89 |
| 1022 | #13 | 25 | 2014 | Female | 66.45 | 61.76 |
| 1023 | #13 | 32 | 2014 | Female | 62.15 | 60.79 |
| 1024 | #13 | 11 | 2014 | Female | 67.45 | 57.16 |
| 1025 | #3 | 38 | 2015 | Male | 68.60 | 65.79 |
| 1026 | #3 | 67 | 2014 | Female | 66.27 | 67.50 |
| 1027 | #3 | 72 | 2014 | Male | 66.15 | 71.67 |
| 1028 | #3 | 31 | 2015 | Female | 70.63 | 66.19 |
| 1029 | #3 | 19 | 2014 | Male | 66.52 | 60.09 |
| 1030 | #3 | 40 | 2015 | Female | 67.27 | 59.51 |
| 1031 | #3 | 52 | 2015 | Male | 74.02 | 70.55 |
| 1032 | #3 | 54 | 2014 | Female | 66.65 | 67.00 |
| 1033 | #3 | 39 | 2014 | Male | 67.73 | 63.70 |
| 1034 | #3 | 16 | 2014 | Male | 67.18 | 61.80 |
| 1035 | #3 | 46 | 2014 | Female | 66.92 | 65.73 |
| 1036 | #4 | 25 | 2015 | Male | 67.94 | 60.56 |
| 1037 | #4 | 58 | 2014 | Female | 64.99 | 65.15 |
| 1038 | #4 | 57 | 2014 | Male | 67.83 | 67.21 |
| 1039 | #4 | 2 | 2014 | Female | 63.18 | 51.07 |
| 1040 | #6 | 1 | 2015 | Female | 67.82 | 50.25 |
| 1041 | #6 | 52 | 2015 | Female | 63.98 | 58.03 |
| 1042 | #4 | 1 | 2015 | Male | 69.11 | 59.93 |
| 1043 | #6 | 66 | 2015 | Female | 64.61 | 69.20 |
| 1044 | #6 | 74 | 2015 | Female | 60.15 | 70.10 |
| 1045 | #6 | 8 | 2015 | Male | NA | NA |
| 1046 | #4 | 4 | 2015 | Female | 62.88 | 45.00 |
| 1047 | #6 | 47 | 2015 | Female | 67.56 | 67.77 |
| 1048 | #6 | 4 | 2015 | Female | 67.88 | 45.76 |
| 1049 | #6 | 47 | 2015 | Female | 66.56 | 67.87 |
| 1050 | #6 | 59 | 2015 | Female | NA | NA |
| 1051 | #5 | 47 | 2015 | Male | 65.90 | 59.66 |
| 1052 | #5 | 52 | 2015 | Male | 71.56 | 65.41 |
| 1053 | #5 | 52 | 2015 | Female | 67.02 | 63.73 |
| 1054 | #5 | 24 | 2015 | Female | NA | NA |
| 1055 | #5 | 33 | 2015 | Male | 68.22 | 57.52 |
| 1056 | #5 | 56 | 2015 | Female | 64.73 | 69.35 |
| 1057 | #5 | 56 | 2015 | Female | 65.61 | 69.37 |
| 1058 | #5 | 43 | 2014 | Female | 66.23 | 63.92 |
| 1059 | #5 | 1 | 2014 | Female | 66.52 | 51.17 |
| 1060 | #5 | 19 | 2015 | Female | 63.68 | 50.76 |
| 1061 | #5 | 22 | 2014 | Female | 67.12 | 61.62 |
| 1062 | #5 | 55 | 2014 | Female | 66.92 | 67.40 |
| 1063 | #5 | 61 | 2014 | Male | 67.23 | 67.76 |
| 1064 | #5 | 28 | 2015 | Male | 70.11 | 59.82 |
| 1065 | #5 | 53 | 2014 | Male | 66.45 | 65.79 |
| 1066 | #5 | 50 | 2014 | Female | 67.06 | 65.39 |
| 1067 | #5 | 34 | 2014 | Female | 66.72 | 63.51 |
| 1068 | #5 | 48 | 2014 | Female | 66.68 | 64.29 |
| 1069 | #5 | 1 | 2015 | Female | 65.87 | 46.82 |
| 1070 | #5 | 33 | 2015 | Female | 68.52 | 65.39 |
| 1071 | #5 | 64 | 2014 | Female | 66.67 | 67.49 |
| 1072 | #5 | 22 | 2014 | Male | 67.24 | 60.90 |
| 1073 | #5 | 33 | 2014 | Female | 66.03 | 62.29 |
| 1074 | #5 | 64 | 2014 | Male | 67.44 | 67.26 |
| 1075 | #5 | 68 | 2014 | Female | 67.83 | 66.74 |
| 1076 | #5 | 44 | 2014 | Female | 66.43 | 66.28 |
| 1077 | #5 | 7 | 2015 | Male | 70.92 | 56.58 |
| 1078 | #5 | 38 | 2015 | Male | 71.52 | 67.31 |
| 1079 | #5 | 71 | 2015 | Female | 79.25 | 61.97 |
| 1080 | #5 | 2 | 2014 | Male | 66.49 | 56.93 |
| 1081 | #6 | 5 | 2014 | Male | 63.25 | 54.17 |
| 1082 | #6 | 53 | 2014 | Female | 66.45 | 70.69 |
| 1083 | #6 | 58 | 2014 | Male | 63.45 | 60.44 |
| 1084 | #6 | 22 | 2015 | Female | 71.14 | 64.76 |
| 1085 | #6 | 55 | 2014 | Female | 66.92 | 65.67 |
| 1086 | #6 | 8 | 2014 | Male | 66.87 | 58.27 |
| 1087 | #6 | 64 | 2014 | Female | 66.20 | 69.67 |
| 1088 | #6 | 74 | 2014 | Male | 70.21 | 73.81 |
| 1089 | #6 | 18 | 2014 | Female | 67.63 | 61.02 |
| 1090 | #6 | 60 | 2015 | Male | 63.46 | 68.93 |
| 1091 | #6 | 74 | 2014 | Female | 68.39 | 70.04 |
| 1092 | #6 | 27 | 2014 | Female | 68.19 | 64.63 |
| 1093 | #6 | 65 | 2014 | Male | 68.21 | 71.06 |
| 1094 | #6 | 62 | 2014 | Male | 69.36 | 71.14 |
| 1095 | #6 | 63 | 2015 | Female | 68.45 | 74.71 |
| 1096 | #6 | 28 | 2015 | Female | 68.58 | 61.05 |
| 1097 | #6 | 46 | 2015 | Male | 66.72 | 70.15 |
| 1098 | #6 | 25 | 2015 | Male | 62.96 | 57.67 |
| 1099 | #6 | 25 | 2015 | Male | NA | NA |
| 1100 | #6 | 29 | 2015 | Male | 67.26 | 61.93 |
| 1101 | #6 | 19 | 2015 | Female | 66.30 | 57.39 |
| 1102 | #6 | 66 | 2015 | Female | 66.05 | 71.72 |
| 1103 | #4 | 67 | 2015 | Female | 65.23 | 71.36 |
| 1104 | #6 | 59 | 2015 | Female | 66.21 | 69.27 |
| 1105 | #6 | 25 | 2015 | Female | 67.93 | 61.07 |
| 1106 | #7 | 17 | 2015 | Male | 66.54 | 47.29 |
| 1107 | #7 | 61 | 2014 | Female | 66.54 | 70.91 |
| 1108 | #7 | 61 | 2014 | Female | 68.42 | 68.80 |
| 1109 | #7 | 26 | 2014 | Male | 66.67 | 60.54 |
| 1110 | #7 | 47 | 2014 | Female | 67.21 | 65.10 |
| 1111 | #7 | 68 | 2014 | Female | 63.01 | 59.33 |
| 1112 | #7 | 71 | 2014 | Female | 67.02 | 66.03 |
| 1113 | #7 | 47 | 2015 | Female | 63.48 | 65.32 |
| 1114 | #7 | 45 | 2015 | Female | 67.99 | 59.72 |
| 1115 | #7 | 33 | 2015 | Male | 70.02 | 63.46 |
| 1116 | #7 | 53 | 2015 | Female | 69.38 | 65.01 |
| 1117 | #7 | 70 | 2015 | Male | 63.60 | 64.15 |
| 1118 | #7 | 24 | 2015 | Female | 65.04 | 59.36 |
| 1119 | #7 | 70 | 2015 | Male | 67.67 | 63.50 |
| 1120 | #7 | 52 | 2015 | Male | 65.25 | 67.14 |
| 1121 | #7 | 67 | 2015 | Male | 67.23 | 67.91 |
| 1122 | #7 | 67 | 2015 | Male | 69.19 | 66.22 |
| 1123 | #7 | 24 | 2015 | Male | 68.46 | 60.91 |
| 1124 | #7 | 60 | 2015 | Female | 66.86 | 62.52 |
| 1125 | #7 | 38 | 2015 | Male | 67.73 | 63.37 |
| 1126 | #7 | 42 | 2015 | Male | 64.50 | 64.37 |
| 1127 | #7 | 15 | 2015 | Male | 66.19 | 59.82 |
| 1128 | #7 | 60 | 2015 | Female | NA | NA |
| 1129 | #7 | 73 | 2015 | Male | 68.06 | 68.43 |
| 1130 | #7 | 73 | 2015 | Male | 70.21 | 68.35 |
| 1131 | #7 | 61 | 2015 | Male | 64.15 | 63.90 |
| 1132 | #7 | 47 | 2015 | Female | 69.07 | 68.68 |
| 1133 | #7 | 50 | 2015 | Male | 65.10 | 64.59 |
| 1134 | #14 | 71 | 2015 | Female | 69.31 | 64.15 |
| 1135 | #14 | 66 | 2015 | Female | 66.38 | 67.29 |
| 1136 | #16 | 64 | 2014 | Female | 71.62 | 70.32 |
| 1137 | #7 | 58 | 2014 | Female | 64.12 | 68.75 |
| 1138 | #7 | 6 | 2014 | Male | 66.45 | 57.47 |
| 1139 | #7 | 69 | 2014 | Male | 66.55 | 67.96 |
| 1140 | #7 | 26 | 2014 | Male | 62.97 | 52.06 |
| 1141 | #7 | 4 | 2014 | Male | 66.54 | 59.78 |
| 1142 | #7 | 71 | 2014 | Male | 66.02 | 67.89 |
| 1143 | #7 | 73 | 2015 | Female | 69.04 | 69.21 |
| 1144 | #7 | 64 | 2014 | Female | 67.31 | 66.87 |
| 1145 | #18 | 53 | 2014 | Male | 64.63 | 75.56 |
| 1146 | #18 | 3 | 2015 | Female | 61.63 | 55.95 |
| 1147 | #18 | 40 | 2014 | Male | 66.19 | 63.85 |
| 1148 | #18 | 66 | 2015 | Female | 66.44 | 68.43 |
| 1149 | #18 | 4 | 2014 | Male | 65.91 | 61.02 |
| 1150 | #18 | 48 | 2014 | Female | 67.82 | 70.70 |
| 1151 | #18 | 64 | 2014 | Female | 65.41 | 66.80 |
| 1152 | #18 | 37 | 2014 | Male | 66.38 | 66.63 |
| 1153 | #18 | 53 | 2014 | Male | 69.13 | 73.82 |
| 1154 | #18 | 54 | 2014 | Male | 65.25 | 71.72 |
| 1155 | #18 | 44 | 2014 | Male | 61.97 | 73.66 |
| 1156 | #18 | 18 | 2015 | Male | 68.58 | 58.46 |
| 1157 | #18 | 43 | 2015 | Male | 70.07 | 66.78 |
| 1158 | #18 | 46 | 2015 | Male | NA | NA |
| 1159 | #18 | 63 | 2015 | Male | 69.56 | 68.10 |
| 1160 | #18 | 70 | 2015 | Male | 69.14 | 71.43 |
| 1161 | #18 | 56 | 2015 | Female | 67.20 | 67.75 |
| 1162 | #18 | 38 | 2015 | Female | 65.00 | 66.70 |
| 1163 | #18 | 66 | 2015 | Female | 67.37 | 71.70 |
| 1164 | #18 | 63 | 2015 | Female | 64.30 | 64.11 |
| 1165 | #18 | 18 | 2015 | Female | 69.46 | 62.23 |
| 1166 | #2 | 54 | 2014 | Male | 67.81 | 65.91 |
| 1167 | #2 | 60 | 2015 | Male | 69.50 | 69.04 |
| 1168 | #2 | 73 | 2015 | Female | 68.08 | 71.94 |
| 1169 | #2 | 44 | 2014 | Female | 66.29 | 66.15 |
| 1170 | #2 | 74 | 2015 | Male | 70.73 | 71.13 |
| 1171 | #2 | 26 | 2014 | Male | 68.76 | 62.29 |
| 1172 | #2 | 39 | 2014 | Male | 67.78 | 64.83 |
| 1173 | #2 | 68 | 2014 | Male | 64.54 | 67.90 |
| 1174 | #2 | 68 | 2014 | Male | 65.23 | 69.68 |
| 1175 | #2 | 67 | 2014 | Female | 68.27 | 68.15 |
| 1176 | #2 | 72 | 2014 | Male | 68.29 | 68.02 |
| 1177 | #2 | 10 | 2015 | Female | 67.71 | 45.06 |
| 1178 | #2 | 64 | 2015 | Male | 64.40 | 65.72 |
| 1179 | #2 | 46 | 2014 | Male | 64.15 | 63.01 |
| 1180 | #2 | 74 | 2014 | Male | 65.34 | 67.67 |
| 1181 | #2 | 38 | 2015 | Male | 67.73 | 67.07 |
| 1182 | #2 | 29 | 2015 | Female | 65.28 | 58.54 |
| 1183 | #2 | 53 | 2015 | Female | 70.62 | 65.71 |
| 1184 | #2 | 68 | 2015 | Male | 67.53 | 66.02 |
| 1185 | #2 | 52 | 2015 | Male | 70.50 | 67.48 |
| 1186 | #2 | 25 | 2015 | Male | 69.00 | 62.60 |
| 1187 | #2 | 60 | 2015 | Female | 67.99 | 66.83 |
| 1188 | #2 | 12 | 2015 | Male | 65.36 | 50.71 |
| 1189 | #2 | 74 | 2015 | Female | 68.98 | 68.38 |
| 1190 | #2 | 59 | 2015 | Male | 72.69 | 70.09 |
| 1191 | #2 | 4 | 2015 | Male | 66.93 | 55.28 |
| 1192 | #2 | 5 | 2015 | Male | 68.60 | 56.48 |
| 1193 | #2 | 5 | 2015 | Male | 69.38 | 56.64 |
| 1194 | #2 | 4 | 2015 | Male | 65.12 | 53.05 |
| 1195 | #2 | 60 | 2015 | Female | NA | NA |
| 1196 | #2 | 53 | 2014 | Male | 69.36 | 65.02 |
| 1197 | #2 | 25 | 2015 | Male | 74.04 | 68.65 |
| 1198 | #2 | 61 | 2015 | Male | 68.15 | 64.31 |
| 1199 | #2 | 62 | 2014 | Female | 68.63 | 64.78 |
| 1200 | #2 | 65 | 2014 | Female | 65.83 | 67.89 |
| 1201 | #2 | 67 | 2014 | Male | 65.32 | 68.32 |
| 1202 | #2 | 4 | 2014 | Male | 68.23 | 54.04 |
| 1203 | #2 | 13 | 2014 | Female | 66.34 | 55.48 |
| 1204 | #2 | 25 | 2014 | Male | 63.52 | 59.86 |
| 1205 | #2 | 11 | 2014 | Male | 65.91 | 61.46 |
| 1206 | #2 | 73 | 2015 | Male | 66.31 | 67.51 |
| 1207 | #2 | 28 | 2015 | Male | 68.44 | 60.54 |
| 1208 | #2 | 70 | 2015 | Female | 68.49 | 62.78 |
| 1209 | #17 | 71 | 2015 | Female | 63.28 | 73.73 |
| 1210 | #17 | 59 | 2015 | Female | 68.79 | 70.24 |
| 1211 | #17 | 73 | 2015 | Male | 65.31 | 70.38 |
| 1212 | #3 | 33 | 2015 | Female | 68.50 | 62.86 |
| 1213 | #3 | 60 | 2014 | Male | 66.08 | 68.48 |
| 1214 | #3 | 9 | 2014 | Male | 67.15 | 58.50 |
| 1215 | #3 | 38 | 2015 | Male | 67.78 | 63.95 |
| 1216 | #3 | 43 | 2014 | Female | 66.31 | 65.67 |
| 1217 | #3 | 60 | 2014 | Female | 64.16 | 63.02 |
| 1218 | #3 | 48 | 2014 | Male | 66.28 | 70.93 |
| 1219 | #3 | 73 | 2015 | Male | 72.80 | 68.98 |
| 1220 | #3 | 64 | 2014 | Male | 67.85 | 67.47 |
| 1221 | #3 | 58 | 2014 | Female | 68.22 | 65.50 |
| 1222 | #3 | 54 | 2015 | Female | 69.90 | 66.42 |
| 1223 | #12 | 10 | 2015 | Female | 71.55 | 58.03 |
| 1224 | #12 | 67 | 2014 | Female | 66.91 | 70.80 |
| 1225 | #12 | 48 | 2015 | Female | 68.32 | 67.19 |
| 1226 | #12 | 49 | 2015 | Male | 65.70 | 66.17 |
| 1227 | #12 | 67 | 2014 | Female | 66.07 | 68.21 |
| 1228 | #12 | 74 | 2014 | Male | 67.81 | 66.07 |
| 1229 | #12 | 46 | 2014 | Male | 66.19 | 70.93 |
| 1230 | #12 | 6 | 2014 | Male | 67.87 | 54.55 |
| 1231 | #12 | 61 | 2014 | Female | 66.45 | 67.09 |
| 1232 | #12 | 61 | 2014 | Male | 66.52 | 67.57 |
| 1233 | #12 | 37 | 2014 | Male | 67.32 | 63.21 |
| 1234 | #12 | 50 | 2014 | Male | 66.43 | 66.40 |
| 1235 | #12 | 45 | 2015 | Female | 66.52 | 68.02 |
| 1236 | #12 | 29 | 2014 | Female | 67.89 | 60.82 |
| 1237 | #12 | 43 | 2015 | Female | 69.25 | 68.19 |
| 1238 | #12 | 59 | 2015 | Female | 63.88 | 65.96 |
| 1239 | #12 | 61 | 2014 | Male | 67.28 | 70.23 |
| 1240 | #12 | 54 | 2014 | Male | 66.58 | 67.16 |
| 1241 | #12 | 25 | 2014 | Male | 66.21 | 62.01 |
| 1242 | #12 | 12 | 2015 | Male | 66.44 | 55.01 |
| 1243 | #12 | 60 | 2014 | Female | 66.81 | 67.21 |
| 1244 | #12 | 71 | 2014 | Male | 70.02 | 72.99 |
| 1245 | #12 | 17 | 2015 | Male | 65.89 | 61.12 |
| 1246 | #12 | 38 | 2015 | Female | 66.96 | 62.71 |
| 1247 | #12 | 73 | 2015 | Female | 68.88 | 68.22 |
| 1248 | #12 | 49 | 2015 | Male | 71.68 | 72.57 |
| 1249 | #12 | 71 | 2015 | Male | 65.20 | 68.67 |
| 1250 | #12 | 11 | 2015 | Male | 67.07 | 58.52 |
| 1251 | #12 | 46 | 2015 | Male | 67.42 | 66.56 |
| 1252 | #12 | 61 | 2015 | Male | 69.65 | 67.95 |
| 1253 | #12 | 15 | 2015 | Female | 66.87 | 56.96 |
| 1254 | #12 | 32 | 2015 | Female | 64.39 | 65.73 |
| 1255 | #12 | 73 | 2015 | Male | 64.55 | 74.17 |
| 1256 | #12 | 73 | 2015 | Male | 64.97 | 75.12 |
| 1257 | #12 | 73 | 2015 | Female | 66.79 | 69.61 |
| 1258 | #12 | 63 | 2015 | Male | 71.02 | 70.67 |
| 1259 | #12 | 73 | 2015 | Male | NA | NA |
| 1260 | #12 | 46 | 2015 | Male | 63.38 | 70.93 |
| 1261 | #12 | 67 | 2015 | Female | 67.34 | 70.34 |
| 1262 | #12 | 67 | 2015 | Female | 65.35 | 72.73 |
| 1263 | #20 | 17 | 2015 | Female | 69.84 | 57.19 |
| 1264 | #20 | 4 | 2015 | Male | 70.67 | 56.44 |
| 1265 | #20 | 38 | 2015 | Male | 68.73 | 61.24 |
| 1266 | #20 | 67 | 2015 | Female | 67.39 | 73.87 |
| 1267 | #20 | 38 | 2015 | Male | 68.77 | 65.30 |
| 1268 | #20 | 38 | 2015 | Male | 66.62 | 67.84 |
| 1269 | #20 | 74 | 2015 | Male | 63.26 | 67.24 |
| 1270 | #20 | 53 | 2015 | Female | 69.55 | 66.63 |
| 1271 | #20 | 53 | 2015 | Male | 69.60 | 68.11 |
| 1272 | #20 | 38 | 2015 | Female | 68.81 | 66.12 |
| 1273 | #20 | 39 | 2015 | Male | 70.30 | 71.42 |
| 1274 | #20 | 35 | 2015 | Male | 68.11 | 63.65 |
| 1275 | #20 | 52 | 2014 | Female | 66.01 | 62.74 |
| 1276 | #20 | 57 | 2014 | Female | 67.86 | 73.97 |
| 1277 | #20 | 56 | 2014 | Male | 65.56 | 63.20 |
| 1278 | #20 | 36 | 2014 | Female | 64.06 | 62.37 |
| 1279 | #20 | 65 | 2014 | Male | 67.16 | 69.05 |
| 1280 | #20 | 47 | 2014 | Male | 67.98 | 63.96 |
| 1281 | #20 | 40 | 2014 | Male | 64.67 | 58.56 |
| 1282 | #20 | 32 | 2014 | Female | 66.23 | 63.01 |
| 1283 | #20 | 18 | 2014 | Female | 67.72 | 56.01 |
| 1284 | #21 | 40 | 2014 | Male | 67.82 | 64.05 |
| 1285 | #21 | 24 | 2015 | Female | 64.13 | 57.25 |
| 1286 | #21 | 39 | 2014 | Male | 66.56 | 65.87 |
| 1287 | #21 | 33 | 2014 | Male | 66.69 | 63.92 |
| 1288 | #21 | 54 | 2014 | Male | 66.58 | 66.44 |
| 1289 | #21 | 26 | 2014 | Female | 66.12 | 61.03 |
| 1290 | #21 | 40 | 2014 | Female | 66.42 | 58.63 |
| 1291 | #21 | 72 | 2014 | Male | 66.01 | 70.07 |
| 1292 | #21 | 52 | 2015 | Male | 67.94 | 62.08 |
| 1293 | #21 | 18 | 2015 | Male | 64.65 | 57.86 |
| 1294 | #21 | 67 | 2014 | Female | 66.57 | 67.08 |
| 1295 | #21 | 62 | 2014 | Female | 66.43 | 68.39 |
| 1296 | #21 | 71 | 2014 | Male | 66.35 | 68.87 |
| 1297 | #21 | 53 | 2014 | Female | 66.31 | 65.81 |
| 1298 | #21 | 39 | 2015 | Female | 66.87 | 68.28 |
| 1299 | #21 | 70 | 2015 | Female | 67.19 | 65.32 |
| 1300 | #21 | 55 | 2014 | Female | 66.22 | 65.85 |
| 1301 | #21 | 47 | 2015 | Male | 65.31 | 63.43 |
| 1302 | #21 | 18 | 2015 | Female | 63.15 | 57.87 |
| 1303 | #21 | 73 | 2015 | Male | 65.27 | 71.14 |
| 1304 | #21 | 60 | 2014 | Male | 67.13 | 66.41 |
| 1305 | #21 | 53 | 2014 | Male | 65.76 | 62.23 |
| 1306 | #21 | 71 | 2015 | Female | 64.58 | 64.50 |
| 1307 | #21 | 61 | 2015 | Female | 64.38 | 64.75 |
| 1308 | #21 | 67 | 2015 | Female | 63.49 | 62.86 |
| 1309 | #21 | 61 | 2015 | Female | 66.34 | 66.17 |
| 1310 | #21 | 70 | 2015 | Female | 63.15 | 66.64 |
| 1311 | #13 | 74 | 2014 | Female | 68.32 | 68.99 |
| 1312 | #13 | 24 | 2015 | Male | 64.12 | 55.31 |
| 1313 | #13 | 24 | 2015 | Female | 63.89 | 57.25 |
| 1314 | #13 | 59 | 2015 | Female | 66.98 | 68.28 |
| 1315 | #13 | 67 | 2014 | Female | 67.84 | 69.07 |
| 1316 | #13 | 74 | 2015 | Male | 67.19 | 69.09 |
| 1317 | #13 | 67 | 2014 | Female | 67.62 | 64.97 |
| 1318 | #13 | 2 | 2014 | Female | 61.40 | 51.32 |
| 1319 | #13 | 53 | 2014 | Male | 71.10 | 70.86 |
| 1320 | #13 | 61 | 2014 | Male | 74.26 | 64.05 |
| 1321 | #13 | 54 | 2014 | Male | 68.02 | 68.23 |
| 1322 | #13 | 67 | 2014 | Female | 67.25 | 67.94 |
| 1323 | #13 | 68 | 2014 | Female | 64.30 | 61.84 |
| 1324 | #13 | 52 | 2015 | Female | 65.87 | 79.26 |
| 1325 | #13 | 61 | 2014 | Female | 66.82 | 66.63 |
| 1326 | #13 | 22 | 2015 | Female | 63.91 | 59.26 |
| 1327 | #13 | 43 | 2015 | Female | 65.93 | 72.94 |
| 1328 | #13 | 71 | 2014 | Male | 67.12 | 71.32 |
| 1329 | #13 | 54 | 2014 | Female | 68.55 | 67.43 |
| 1330 | #13 | 64 | 2015 | Female | 66.74 | 70.40 |
| 1331 | #13 | 59 | 2015 | Female | 66.12 | 72.99 |
| 1332 | #13 | 15 | 2015 | Male | 65.22 | 63.22 |
| 1333 | #13 | 42 | 2015 | Female | 66.37 | 65.88 |
| 1334 | #13 | 73 | 2015 | Male | 66.37 | 72.54 |
| 1335 | #13 | 59 | 2015 | Female | 66.24 | 71.93 |
| 1336 | #13 | 67 | 2015 | Female | 65.14 | 68.13 |
| 1337 | #13 | 61 | 2015 | Female | 66.91 | 73.24 |
| 1338 | #13 | 50 | 2015 | Female | 66.57 | 70.26 |
| 1339 | #13 | 63 | 2015 | Female | 65.88 | 68.14 |
| 1340 | #13 | 47 | 2015 | Male | 65.13 | 63.87 |
| 1341 | #13 | 42 | 2015 | Male | 66.81 | 67.40 |
| 1342 | #13 | 46 | 2015 | Female | 65.94 | 67.24 |
| 1343 | #13 | 33 | 2015 | Female | 67.18 | 70.53 |
| 1344 | #13 | 68 | 2015 | Male | 66.98 | 68.64 |
| 1345 | #13 | 73 | 2015 | Female | 67.45 | 72.58 |
| 1346 | #13 | 26 | 2015 | Female | 65.11 | 68.76 |
| 1347 | #13 | 4 | 2015 | Male | 64.12 | 51.86 |
| 1348 | #13 | 46 | 2015 | Female | 66.12 | 65.76 |
| 1349 | #13 | 70 | 2015 | Male | 66.97 | 68.04 |
| 1350 | #13 | 70 | 2015 | Female | 68.24 | 72.05 |
| 1351 | #13 | 66 | 2015 | Female | 68.79 | 74.18 |
| 1352 | #15 | 31 | 2015 | Female | 67.48 | 60.38 |
| 1353 | #15 | 24 | 2015 | Male | 68.41 | 56.61 |
| 1354 | #15 | 25 | 2015 | Female | 67.48 | 63.46 |
| 1355 | #15 | 38 | 2015 | Male | 65.29 | 67.60 |
| 1356 | #15 | 45 | 2015 | Female | 66.89 | 71.12 |
| 1357 | #15 | 22 | 2015 | Male | 68.11 | 62.30 |
| 1358 | #15 | 59 | 2015 | Male | 68.75 | 70.04 |
| 1359 | #15 | 61 | 2015 | Male | 68.73 | 73.58 |
| 1360 | #15 | 63 | 2015 | Female | 66.49 | 62.65 |
| 1361 | #15 | 58 | 2014 | Female | 66.23 | 68.28 |
| 1362 | #15 | 48 | 2014 | Female | 65.85 | 62.82 |
| 1363 | #15 | 37 | 2014 | Female | 65.49 | 66.02 |
| 1364 | #15 | 25 | 2014 | Female | 67.91 | 66.24 |
| 1365 | #15 | 67 | 2014 | Female | 70.44 | 72.12 |
| 1366 | #15 | 32 | 2014 | Male | 67.19 | 66.40 |
| 1367 | #15 | 46 | 2014 | Male | 66.97 | 66.54 |
| 1368 | #15 | 15 | 2014 | Male | 65.03 | 57.49 |
| 1369 | #15 | 26 | 2014 | Female | 63.84 | 60.15 |
| 1370 | #15 | 50 | 2014 | Female | 68.27 | 68.90 |
| 1371 | #15 | 20 | 2014 | Female | 65.57 | 60.95 |
| 1372 | #15 | 67 | 2014 | Male | 67.43 | 62.84 |
| 1373 | #15 | 70 | 2015 | Male | 67.89 | 63.39 |
| 1374 | #15 | 25 | 2014 | Male | 62.01 | 55.58 |
| 1375 | #15 | 74 | 2014 | Male | 66.97 | 64.55 |
| 1376 | #15 | 64 | 2015 | Male | 68.47 | 67.02 |
| 1377 | #15 | 54 | 2014 | Female | 66.59 | 62.87 |
| 1378 | #15 | 26 | 2015 | Female | 65.98 | 54.15 |
| 1379 | #15 | 18 | 2014 | Female | 64.57 | 55.68 |
| 1380 | #15 | 44 | 2014 | Male | 68.74 | 68.48 |
| 1381 | #15 | 44 | 2014 | Male | 70.11 | 69.17 |
| 1382 | #15 | 69 | 2014 | Male | 66.38 | 66.16 |
| 1383 | #10 | 59 | 2015 | Female | 66.13 | 66.38 |
| 1384 | #10 | 74 | 2015 | Male | 67.48 | 68.95 |
| 1385 | #10 | 67 | 2014 | Female | 66.51 | 68.04 |
| 1386 | #10 | 32 | 2014 | Female | 67.73 | 64.53 |
| 1387 | #10 | 12 | 2014 | Female | 67.54 | 56.59 |
| 1388 | #10 | 61 | 2014 | Male | 66.75 | 69.84 |
| 1389 | #10 | 47 | 2014 | Female | 67.81 | 67.01 |
| 1390 | #10 | 60 | 2014 | Female | 67.82 | 71.32 |
| 1391 | #10 | 27 | 2014 | Male | 67.89 | 62.53 |
| 1392 | #10 | 66 | 2015 | Male | 68.97 | 68.44 |
| 1393 | #10 | 56 | 2015 | Female | 66.31 | 64.77 |
| 1394 | #10 | 74 | 2014 | Female | 68.04 | 71.98 |
| 1395 | #10 | 48 | 2014 | Female | 66.07 | 68.38 |
| 1396 | #10 | 44 | 2014 | Female | 67.02 | 66.50 |
| 1397 | #10 | 35 | 2015 | Female | 63.88 | 62.91 |
| 1398 | #10 | 42 | 2015 | Male | 64.19 | 64.60 |
| 1399 | #10 | 74 | 2015 | Female | 68.14 | 68.47 |
| 1400 | #10 | 46 | 2015 | Male | 65.14 | 67.71 |
| 1401 | #10 | 33 | 2014 | Female | 67.29 | 64.43 |
| 1402 | #10 | 60 | 2014 | Female | 69.11 | 64.60 |
| 1403 | #10 | 39 | 2014 | Male | 65.93 | 64.94 |
| 1404 | #10 | 67 | 2014 | Female | 69.41 | 69.15 |
| 1405 | #10 | 37 | 2014 | Male | 66.52 | 63.57 |
| 1406 | #10 | 32 | 2015 | Female | 66.15 | 61.62 |
| 1407 | #10 | 46 | 2014 | Female | 67.22 | 67.85 |
| 1408 | #10 | 68 | 2014 | Female | 66.59 | 66.99 |
| 1409 | #10 | 51 | 2014 | Female | 67.89 | 64.65 |
| 1410 | #10 | 27 | 2014 | Female | 66.32 | 64.99 |
| 1411 | #10 | 15 | 2014 | Male | 67.02 | 60.85 |
| 1412 | #10 | 25 | 2014 | Female | 67.85 | 61.13 |
| 1413 | #10 | 69 | 2014 | Female | 66.27 | 67.36 |
| 1414 | #10 | 37 | 2014 | Female | 67.66 | 67.10 |
| 1415 | #10 | 74 | 2014 | Female | 66.33 | 69.96 |
| 1416 | #10 | 39 | 2014 | Female | 67.89 | 66.20 |
| 1417 | #10 | 61 | 2014 | Male | 69.44 | 70.04 |
| 1418 | #10 | 67 | 2014 | Female | 70.55 | 69.50 |
| 1419 | #10 | 37 | 2014 | Female | 67.27 | 63.96 |
| 1420 | #10 | 42 | 2015 | Female | 66.87 | 65.69 |
| 1421 | #10 | 73 | 2015 | Male | 68.31 | 68.83 |
| 1422 | #10 | 73 | 2015 | Male | 68.15 | 70.26 |
| 1423 | #10 | 54 | 2015 | Female | 68.47 | 68.02 |
| 1424 | #10 | 61 | 2014 | Male | 68.55 | 66.53 |
| 1425 | #10 | 72 | 2014 | Female | 66.71 | 72.83 |
| 1426 | #10 | 47 | 2014 | Male | 66.37 | 66.10 |
| 1427 | #10 | 43 | 2014 | Female | 65.92 | 66.80 |
| 1428 | #10 | 17 | 2015 | Female | 66.31 | 57.23 |
| 1429 | #10 | 54 | 2014 | Male | 67.89 | 64.76 |
| 1430 | #10 | 73 | 2015 | Male | 66.31 | 67.97 |
| 1431 | #10 | 19 | 2015 | Male | 65.13 | 61.61 |
| 1432 | #10 | 44 | 2014 | Male | 66.32 | 67.55 |
| 1433 | #10 | 63 | 2015 | Female | 63.52 | 67.50 |
| 1434 | #10 | 3 | 2015 | Female | 63.54 | 52.71 |
| 1435 | #10 | 3 | 2015 | Female | 64.12 | 53.13 |
| 1436 | #10 | 54 | 2015 | Male | 66.35 | 66.19 |
| 1437 | #10 | 19 | 2014 | Male | 67.79 | 60.99 |
| 1438 | #14 | 34 | 2014 | Female | 66.22 | 65.09 |
| 1439 | #14 | 6 | 2014 | Female | 67.98 | 49.23 |
| 1440 | #14 | 67 | 2014 | Male | 66.43 | 67.04 |
| 1441 | #14 | 12 | 2014 | Male | 66.43 | 52.11 |
| 1442 | #14 | 36 | 2014 | Male | 66.87 | 65.06 |
| 1443 | #14 | 74 | 2014 | Female | 68.37 | 71.91 |
| 1444 | #14 | 6 | 2014 | Female | 66.73 | 58.25 |
| 1445 | #14 | 39 | 2014 | Female | 67.03 | 64.92 |
| 1446 | #14 | 61 | 2014 | Male | 67.31 | 68.79 |
| 1447 | #14 | 46 | 2015 | Male | 67.18 | 60.58 |
| 1448 | #14 | 53 | 2014 | Male | 67.78 | 67.40 |
| 1449 | #14 | 71 | 2014 | Male | 67.05 | 70.03 |
| 1450 | #14 | 24 | 2015 | Female | 65.12 | 63.16 |
| 1451 | #14 | 54 | 2014 | Female | 66.78 | 64.85 |
| 1452 | #14 | 1 | 2014 | Male | 66.54 | 59.22 |
| 1453 | #14 | 21 | 2015 | Female | 64.19 | 62.95 |
| 1454 | #14 | 21 | 2015 | Female | 66.13 | 62.41 |
| 1455 | #14 | 22 | 2014 | Female | 66.57 | 61.87 |
| 1456 | #14 | 61 | 2014 | Male | 65.31 | 63.94 |
| 1457 | #14 | 53 | 2014 | Female | 62.65 | 59.43 |
| 1458 | #14 | 39 | 2014 | Female | 64.74 | 63.02 |
| 1459 | #14 | 47 | 2014 | Male | 65.79 | 64.39 |
| 1460 | #14 | 68 | 2014 | Male | 66.92 | 66.41 |
| 1461 | #14 | 25 | 2014 | Female | 66.32 | 63.43 |
| 1462 | #14 | 26 | 2014 | Female | 66.98 | 62.98 |
| 1463 | #14 | 32 | 2014 | Male | 68.28 | 70.37 |
| 1464 | #14 | 57 | 2014 | Male | 69.08 | 72.93 |
| 1465 | #14 | 61 | 2014 | Female | 65.87 | 62.17 |
| 1466 | #14 | 48 | 2014 | Female | 66.11 | 63.32 |
| 1467 | #14 | 19 | 2014 | Male | 65.32 | 53.07 |
| 1468 | #14 | 32 | 2014 | Male | 66.41 | 62.94 |
| 1469 | #14 | 53 | 2014 | Female | 66.02 | 66.16 |
| 1470 | #14 | 2 | 2014 | Male | 66.31 | 57.45 |
| 1471 | #14 | 68 | 2014 | Female | 65.44 | 64.38 |
| 1472 | #14 | 22 | 2014 | Female | 64.96 | 58.58 |
| 1473 | #14 | 58 | 2014 | Female | 64.85 | 61.21 |
| 1474 | #14 | 47 | 2014 | Male | 68.79 | 72.35 |
| 1475 | #14 | 47 | 2014 | Male | 66.47 | 69.26 |
| 1476 | #14 | 42 | 2015 | Male | 68.01 | 60.93 |
| 1477 | #14 | 46 | 2014 | Female | 67.64 | 72.05 |
| 1478 | #14 | 74 | 2014 | Female | 69.01 | 74.70 |
| 1479 | #14 | 66 | 2015 | Female | 69.81 | 70.40 |
| 1480 | #14 | 61 | 2014 | Male | 64.18 | 63.90 |
| 1481 | #14 | 10 | 2015 | Male | 65.31 | 58.88 |
| 1482 | #14 | 68 | 2014 | Female | 66.32 | 71.66 |
| 1483 | #14 | 39 | 2014 | Female | 66.08 | 62.44 |
| 1484 | #14 | 50 | 2015 | Male | 68.15 | 63.53 |
| 1485 | #14 | 69 | 2014 | Female | 65.56 | 63.99 |
| 1486 | #14 | 45 | 2015 | Male | 68.17 | 64.10 |
| 1487 | #14 | 29 | 2015 | Female | 66.38 | 64.03 |
| 1488 | #14 | 43 | 2014 | Female | 66.34 | 64.81 |
| 1489 | #14 | 54 | 2014 | Female | 66.76 | 69.99 |
| 1490 | #14 | 74 | 2014 | Male | 68.43 | 74.14 |
| 1491 | #14 | 61 | 2015 | Male | 69.85 | 72.18 |
| 1492 | #14 | 67 | 2014 | Male | 67.14 | 71.22 |
| 1493 | #14 | 67 | 2014 | Female | 66.41 | 66.32 |
| 1494 | #14 | 71 | 2014 | Male | 65.47 | 63.49 |
| 1495 | #14 | 55 | 2014 | Male | 65.03 | 59.98 |
| 1496 | #14 | 74 | 2014 | Male | 64.10 | 59.61 |
| 1497 | #14 | 22 | 2014 | Female | 65.21 | 63.00 |
| 1498 | #14 | 57 | 2014 | Female | 65.71 | 65.50 |
| 1499 | #16 | 57 | 2014 | Female | 63.69 | 71.21 |
| 1500 | #16 | 55 | 2014 | Male | 69.59 | 67.69 |
| 1501 | #16 | 58 | 2014 | Male | 65.93 | 66.25 |
| 1502 | #16 | 62 | 2014 | Female | 61.48 | 73.78 |
| 1503 | #16 | 40 | 2014 | Female | 68.24 | 64.23 |
| 1504 | #16 | 71 | 2014 | Male | 67.14 | 70.36 |
| 1505 | #16 | 61 | 2014 | Female | 66.16 | 67.48 |
| 1506 | #16 | 74 | 2014 | Male | 69.89 | 76.93 |
| 1507 | #16 | 74 | 2014 | Male | 69.92 | 74.39 |
| 1508 | #16 | 71 | 2014 | Male | 67.35 | 64.19 |
| 1509 | #16 | 60 | 2014 | Female | 65.47 | 69.66 |
| 1510 | #16 | 29 | 2014 | Female | 67.65 | 60.12 |
| 1511 | #16 | 64 | 2014 | Female | 66.21 | 66.10 |
| 1512 | #16 | 58 | 2014 | Female | 71.29 | 68.82 |
| 1513 | #16 | 54 | 2014 | Male | 66.07 | 67.49 |
| 1514 | #16 | 44 | 2014 | Female | 72.90 | 70.28 |
| 1515 | #16 | 69 | 2014 | Male | 67.57 | 73.39 |
| 1516 | #16 | 70 | 2015 | Male | 65.50 | 66.60 |
| 1517 | #16 | 50 | 2014 | Male | 65.34 | 73.53 |
| 1518 | #16 | 57 | 2014 | Male | 58.12 | 67.90 |
| 1519 | #16 | 67 | 2014 | Female | 68.01 | 66.38 |
| 1520 | #16 | 25 | 2014 | Male | 68.67 | 62.64 |
| 1521 | #16 | 26 | 2014 | Female | 65.28 | 63.03 |
| 1522 | #16 | 39 | 2014 | Female | 69.99 | 65.56 |
| 1523 | #16 | 47 | 2014 | Male | 66.70 | 70.44 |
| 1524 | #16 | 72 | 2014 | Female | 64.84 | 72.57 |
| 1525 | #16 | 64 | 2015 | Female | 68.04 | 68.66 |
| 1526 | #16 | 53 | 2014 | Female | 67.77 | 71.93 |
| 1527 | #16 | 65 | 2014 | Female | 65.24 | 66.52 |
| 1528 | #16 | 67 | 2014 | Female | 68.95 | 70.84 |
| 1529 | #16 | 46 | 2015 | Male | 66.25 | 65.41 |
| 1530 | #16 | 71 | 2015 | Male | 67.35 | 71.44 |
| 1531 | #16 | 43 | 2015 | Female | NA | NA |
| 1532 | #16 | 50 | 2015 | Male | 66.97 | 71.49 |
| 1533 | #16 | 60 | 2015 | Male | 70.04 | 74.87 |
| 1534 | #16 | 68 | 2015 | Male | 68.88 | 69.46 |
| 1535 | #16 | 38 | 2015 | Female | 72.79 | 69.19 |
| 1536 | #16 | 42 | 2015 | Male | 65.14 | 68.84 |
| 1537 | #16 | 49 | 2015 | Male | 72.50 | 71.24 |
| 1538 | #16 | 57 | 2015 | Female | 63.71 | 68.45 |
| 1539 | #16 | 63 | 2015 | Male | 65.69 | 67.75 |
| 1540 | #16 | 52 | 2015 | Male | NA | NA |
| 1541 | #16 | 60 | 2015 | Female | 65.89 | 66.56 |
| 1542 | #16 | 66 | 2015 | Female | 68.42 | 66.88 |
| 1543 | #16 | 39 | 2015 | Female | 64.52 | 59.02 |
| 1544 | #16 | 31 | 2015 | Female | 70.47 | 63.73 |
| 1545 | #16 | 53 | 2015 | Male | 66.03 | 70.01 |
| 1546 | #1 | 74 | 2014 | Male | 67.61 | 65.93 |
| 1547 | #1 | 38 | 2015 | Male | 83.16 | 51.88 |
| 1548 | #1 | 33 | 2014 | Female | 67.02 | 66.65 |
| 1549 | #1 | 54 | 2014 | Male | 67.53 | 67.23 |
| 1550 | #1 | 49 | 2015 | Female | 68.35 | 68.43 |
| 1551 | #1 | 39 | 2014 | Male | 68.20 | 67.82 |
| 1552 | #1 | 54 | 2014 | Male | 67.18 | 70.65 |
| 1553 | #1 | 61 | 2014 | Female | 67.37 | 67.18 |
| 1554 | #1 | 68 | 2015 | Female | 65.77 | 63.39 |
| 1555 | #1 | 74 | 2014 | Female | 66.11 | 67.70 |
| 1556 | #1 | 40 | 2014 | Female | 66.04 | 66.47 |
| 1557 | #1 | 47 | 2014 | Male | 67.58 | 65.41 |
| 1558 | #1 | 69 | 2014 | Female | 70.03 | 68.12 |
| 1559 | #1 | 53 | 2014 | Male | 66.92 | 66.82 |
| 1560 | #1 | 47 | 2014 | Female | 67.59 | 66.13 |
| 1561 | #1 | 64 | 2014 | Male | 67.92 | 67.39 |
| 1562 | #1 | 67 | 2014 | Male | 66.98 | 66.71 |
| 1563 | #1 | 53 | 2014 | Female | 67.06 | 67.85 |
| 1564 | #1 | 1 | 2014 | Female | 67.31 | 45.95 |
| 1565 | #1 | 23 | 2014 | Female | 66.71 | 66.12 |
| 1566 | #1 | 41 | 2014 | Female | 67.44 | 66.71 |
| 1567 | #1 | 48 | 2014 | Male | 66.46 | 66.81 |
| 1568 | #1 | 71 | 2014 | Male | 66.59 | 66.69 |
| 1569 | #1 | 37 | 2014 | Male | 66.55 | 64.56 |
| 1570 | #1 | 40 | 2014 | Male | 67.42 | 65.50 |
| 1571 | #1 | 46 | 2014 | Male | 67.23 | 72.53 |
| 1572 | #1 | 72 | 2014 | Female | 66.25 | 68.26 |
| 1573 | #1 | 32 | 2014 | Female | 67.02 | 67.31 |
| 1574 | #1 | 33 | 2014 | Male | 67.39 | 62.48 |
| 1575 | #1 | 54 | 2014 | Female | 68.97 | 70.51 |
| 1576 | #1 | 73 | 2015 | Female | NA | NA |
| 1577 | #1 | 40 | 2014 | Female | 69.38 | 68.23 |
| 1578 | #1 | 40 | 2015 | Female | 73.40 | 66.52 |
| 1579 | #1 | 40 | 2015 | Female | 70.13 | 69.23 |
| 1580 | #1 | 47 | 2014 | Female | 67.27 | 68.16 |
| 1581 | #1 | 39 | 2014 | Female | 67.72 | 66.69 |
| 1582 | #1 | 26 | 2015 | Female | 68.78 | 65.02 |
| 1583 | #1 | 51 | 2014 | Female | 67.41 | 68.41 |
| 1584 | #1 | 56 | 2015 | Male | 64.13 | 62.99 |
| 1585 | #1 | 55 | 2014 | Female | 67.19 | 66.38 |
| 1586 | #1 | 43 | 2015 | Female | 62.60 | 62.13 |
| 1587 | #1 | 53 | 2014 | Male | 66.98 | 66.87 |
| 1588 | #1 | 61 | 2014 | Male | 67.07 | 68.70 |
| 1589 | #1 | 43 | 2014 | Male | 66.62 | 66.53 |
| 1590 | #1 | 43 | 2015 | Female | 68.86 | 69.88 |
| 1591 | #1 | 74 | 2014 | Male | 67.32 | 69.33 |
| 1592 | #1 | 22 | 2015 | Female | 64.12 | 62.13 |
| 1593 | #1 | 71 | 2014 | Male | 67.44 | 67.95 |
| 1594 | #1 | 71 | 2015 | Male | NA | NA |
| 1595 | #1 | 5 | 2015 | Male | 63.38 | 56.20 |
| 1596 | #1 | 24 | 2015 | Male | NA | NA |
| 1597 | #1 | 24 | 2015 | Male | NA | NA |
| 1598 | #1 | 73 | 2015 | Male | 69.96 | 70.29 |
| 1599 | #1 | 63 | 2015 | Male | 68.32 | 58.09 |
| 1600 | #1 | 66 | 2015 | Female | 71.81 | 72.85 |
| 1601 | #1 | 8 | 2015 | Male | 63.01 | 57.98 |
| 1602 | #1 | 73 | 2015 | Female | 64.71 | 59.65 |
| 1603 | #1 | 73 | 2015 | Male | 64.40 | 65.68 |
| 1604 | #1 | 11 | 2015 | Female | 64.15 | 57.13 |
| 1605 | #1 | 59 | 2015 | Male | 67.93 | 72.73 |
| 1606 | #1 | 57 | 2015 | Male | 72.69 | 71.66 |
| 1607 | #1 | 35 | 2015 | Male | 71.91 | 66.21 |
| 1608 | #1 | 73 | 2015 | Female | 66.55 | 68.02 |
| 1609 | #1 | 24 | 2015 | Female | 65.34 | 60.68 |
| 1610 | #1 | 67 | 2015 | Female | 69.33 | 74.21 |
| 1611 | #1 | 67 | 2015 | Female | 68.02 | 67.17 |
| 1612 | #1 | 7 | 2015 | Male | NA | NA |
| 1613 | #1 | 38 | 2015 | Female | 68.86 | 66.55 |
| 1614 | #1 | 49 | 2015 | Female | 69.65 | 65.67 |
| 1615 | #1 | 67 | 2015 | Male | 67.92 | 69.52 |
| 1616 | #1 | 73 | 2015 | Female | 69.40 | 71.38 |
| 1617 | #1 | 36 | 2015 | Female | 66.90 | 67.60 |
| 1618 | #1 | 25 | 2015 | Female | 63.71 | 57.41 |
| 1619 | #1 | 42 | 2015 | Male | 69.32 | 69.31 |
| 1620 | #1 | 67 | 2015 | Female | 66.01 | 65.98 |
| 1621 | #1 | 24 | 2015 | Male | 69.87 | 65.08 |
| 1622 | #1 | 24 | 2015 | Male | 68.63 | 62.52 |
| 1623 | #1 | 4 | 2015 | Male | 69.45 | 54.45 |
| 1624 | #1 | 38 | 2015 | Female | NA | NA |
| 1625 | #1 | 17 | 2015 | Male | 67.40 | 58.96 |
| 1626 | #1 | 25 | 2015 | Male | 66.22 | 58.16 |
| 1627 | #1 | 59 | 2015 | Male | NA | NA |
| 1628 | #1 | 47 | 2015 | Male | 68.72 | 65.41 |
| 1629 | #1 | 54 | 2015 | Female | 64.79 | 64.66 |
| 1630 | #1 | 64 | 2015 | Female | 65.95 | 66.91 |
| 1631 | #1 | 45 | 2015 | Male | 67.56 | 66.05 |
| 1632 | #1 | 50 | 2015 | Male | 68.10 | 66.07 |
| 1633 | #1 | 28 | 2015 | Female | 63.29 | 61.60 |
| 1634 | #1 | 56 | 2015 | Male | 69.02 | 68.26 |
| 1635 | #1 | 21 | 2015 | Male | 68.57 | 68.02 |
| 1636 | #1 | 56 | 2015 | Female | 68.45 | 70.91 |
| 1637 | #1 | 60 | 2015 | Male | 56.02 | 70.92 |
| 1638 | #1 | 59 | 2015 | Male | 68.26 | 66.18 |
| 1639 | #1 | 15 | 2015 | Female | 69.60 | 62.46 |
| 1640 | #1 | 17 | 2015 | Male | 71.40 | 61.40 |
| 1641 | #1 | 38 | 2015 | Female | 68.29 | 71.07 |
| 1642 | #1 | 59 | 2015 | Female | 66.51 | 66.71 |
| 1643 | #1 | 28 | 2015 | Male | 68.51 | 66.16 |
| 1644 | #1 | 66 | 2015 | Female | 62.96 | 61.44 |
| 1645 | #1 | 66 | 2015 | Male | 67.90 | 67.37 |
| 1646 | #1 | 4 | 2015 | Female | 82.83 | 29.76 |
| 1647 | #1 | 73 | 2015 | Male | 65.15 | 69.60 |
| 1648 | #1 | 38 | 2015 | Female | 65.92 | 60.60 |
| 1649 | #1 | 45 | 2015 | Male | 70.29 | 63.77 |
| 1650 | #1 | 59 | 2015 | Male | 65.83 | 61.23 |
| 1651 | #1 | 39 | 2015 | Female | 66.93 | 62.08 |
| 1652 | #11 | 21 | 2015 | Male | 70.28 | 61.72 |
| 1653 | #11 | 10 | 2015 | Male | 70.97 | 53.90 |
| 1654 | #11 | 5 | 2015 | Male | 68.30 | 58.80 |
| 1655 | #11 | 4 | 2015 | Female | 71.70 | 47.39 |
| 1656 | #11 | 67 | 2015 | Female | 71.25 | 70.94 |
| 1657 | #11 | 22 | 2015 | Female | 71.71 | 63.89 |
| 1658 | #11 | 14 | 2015 | Male | 69.08 | 60.16 |
| 1659 | #11 | 59 | 2015 | Female | 63.89 | 68.18 |
| 1660 | #11 | 60 | 2015 | Male | 69.76 | 68.51 |
| 1661 | #11 | 32 | 2015 | Female | 69.25 | 65.79 |
| 1662 | #11 | 10 | 2015 | Female | 62.57 | 45.43 |
| 1663 | #11 | 24 | 2015 | Female | 63.91 | 57.16 |
| 1664 | #11 | 52 | 2015 | Female | 74.72 | 68.05 |
| 1665 | #11 | 66 | 2015 | Female | 68.26 | 69.95 |
| 1666 | #11 | 68 | 2014 | Male | 68.21 | 69.36 |
| 1667 | #11 | 4 | 2015 | Female | 68.46 | 49.53 |
| 1668 | #11 | 32 | 2015 | Male | 67.82 | 59.33 |
| 1669 | #11 | 4 | 2015 | Female | 71.29 | 51.93 |
| 1670 | #11 | 60 | 2014 | Male | 69.34 | 71.89 |
| 1671 | #11 | 32 | 2015 | Male | 69.52 | 65.03 |
| 1672 | #11 | 67 | 2015 | Female | 67.89 | 68.35 |
| 1673 | #11 | 55 | 2014 | Female | 65.98 | 67.59 |
| 1674 | #11 | 39 | 2014 | Female | 66.75 | 65.23 |
| 1675 | #11 | 39 | 2014 | Male | 67.43 | 64.36 |
| 1676 | #11 | 47 | 2014 | Male | 67.56 | 67.52 |
| 1677 | #11 | 68 | 2014 | Male | 66.34 | 68.92 |
| 1678 | #11 | 47 | 2014 | Female | 68.31 | 68.54 |
| 1679 | #11 | 25 | 2014 | Female | 66.28 | 66.72 |
| 1680 | #11 | 2 | 2014 | Male | 66.82 | 60.97 |
| 1681 | #11 | 4 | 2014 | Male | 67.32 | 60.26 |
| 1682 | #11 | 25 | 2014 | Female | 67.43 | 65.62 |
| 1683 | #11 | 57 | 2014 | Male | 68.15 | 64.36 |
| 1684 | #11 | 61 | 2014 | Female | 66.97 | 67.02 |
| 1685 | #11 | 71 | 2015 | Female | 68.16 | 64.79 |
| 1686 | #11 | 3 | 2015 | Male | 71.41 | 58.72 |
| 1687 | #11 | 11 | 2014 | Male | 68.20 | 63.01 |
| 1688 | #11 | 36 | 2014 | Male | 67.32 | 65.22 |
| 1689 | #11 | 25 | 2014 | Female | 67.34 | 63.39 |
| 1690 | #11 | 53 | 2014 | Female | 67.54 | 67.97 |
| 1691 | #11 | 39 | 2014 | Female | 66.43 | 65.01 |
| 1692 | #11 | 20 | 2014 | Male | 66.86 | 59.83 |
| 1693 | #11 | 22 | 2014 | Male | 66.95 | 62.13 |
| 1694 | #11 | 68 | 2014 | Male | 65.14 | 63.80 |
| 1695 | #11 | 1 | 2015 | Female | 69.32 | 51.25 |
| 1696 | #11 | 40 | 2014 | Male | 66.92 | 64.00 |
| 1697 | #11 | 65 | 2014 | Male | 68.52 | 73.78 |
| 1698 | #11 | 68 | 2014 | Female | 67.81 | 73.04 |
| 1699 | #11 | 22 | 2015 | Female | 68.71 | 62.68 |
| 1700 | #11 | 61 | 2014 | Male | 68.24 | 68.47 |
| 1701 | #11 | 18 | 2014 | Female | 65.37 | 50.79 |
| 1702 | #11 | 55 | 2014 | Female | 66.75 | 67.10 |
| 1703 | #11 | 8 | 2014 | Male | 66.84 | 56.58 |
| 1704 | #11 | 20 | 2014 | Male | 66.57 | 59.46 |
| 1705 | #11 | 54 | 2014 | Female | 67.86 | 66.37 |
| 1706 | #11 | 72 | 2014 | Male | 66.94 | 67.92 |
| 1707 | #11 | 5 | 2014 | Male | 66.92 | 52.05 |
| 1708 | #11 | 70 | 2015 | Male | 66.39 | 64.49 |
| 1709 | #11 | 25 | 2014 | Female | 67.38 | 67.37 |
| 1710 | #11 | 25 | 2014 | Male | 67.03 | 66.48 |
| 1711 | #11 | 39 | 2014 | Female | 66.92 | 64.57 |
| 1712 | #11 | 31 | 2015 | Female | 65.70 | 61.84 |
| 1713 | #11 | 8 | 2015 | Male | 68.40 | 56.75 |
| 1714 | #11 | 1 | 2015 | Male | 67.89 | 58.08 |
| 1715 | #11 | 64 | 2015 | Female | 68.34 | 72.15 |
| 1716 | #11 | 73 | 2015 | Female | 69.20 | 71.27 |
| 1717 | #11 | 10 | 2015 | Female | 68.38 | 49.40 |
| 1718 | #11 | 25 | 2015 | Female | 69.46 | 66.97 |
| 1719 | #11 | 53 | 2014 | Male | 68.31 | 68.11 |
| 1720 | #11 | 29 | 2014 | Male | 68.24 | 65.88 |
| 1721 | #8 | 60 | 2014 | Female | 68.31 | 76.35 |
| 1722 | #8 | 60 | 2014 | Female | 72.52 | 73.22 |
| 1723 | #8 | 46 | 2014 | Male | 64.30 | 61.11 |
| 1724 | #8 | 67 | 2014 | Female | 70.87 | 69.59 |
| 1725 | #8 | 67 | 2014 | Female | 69.09 | 69.18 |
| 1726 | #8 | 72 | 2014 | Male | 67.42 | 66.45 |
| 1727 | #8 | 26 | 2014 | Female | 66.78 | 62.14 |
| 1728 | #8 | 25 | 2014 | Female | 65.90 | 62.13 |
| 1729 | #8 | 46 | 2014 | Female | 66.74 | 65.74 |
| 1730 | #8 | 62 | 2014 | Female | 66.54 | 69.43 |
| 1731 | #8 | 22 | 2015 | Male | 68.44 | 62.56 |
| 1732 | #8 | 74 | 2014 | Male | 66.82 | 68.50 |
| 1733 | #8 | 55 | 2014 | Female | 66.34 | 66.92 |
| 1734 | #8 | 54 | 2015 | Female | 67.95 | 70.17 |
| 1735 | #8 | 60 | 2014 | Female | 68.48 | 66.24 |
| 1736 | #8 | 65 | 2014 | Female | 69.87 | 70.96 |
| 1737 | #8 | 25 | 2014 | Female | 66.05 | 56.97 |
| 1738 | #8 | 25 | 2014 | Female | 67.66 | 63.88 |
| 1739 | #8 | 53 | 2014 | Female | 64.38 | 63.99 |
| 1740 | #8 | 64 | 2014 | Male | 66.88 | 69.62 |
| 1741 | #8 | 46 | 2015 | Female | 68.33 | 68.08 |
| 1742 | #8 | 52 | 2015 | Female | 70.09 | 72.17 |
| 1743 | #8 | 46 | 2014 | Female | 66.75 | 67.50 |
| 1744 | #8 | 71 | 2014 | Female | 66.74 | 65.98 |
| 1745 | #8 | 25 | 2014 | Male | 66.95 | 62.30 |
| 1746 | #8 | 19 | 2014 | Female | 63.75 | 55.92 |
| 1747 | #8 | 57 | 2014 | Male | 70.82 | 74.20 |
| 1748 | #8 | 61 | 2015 | Female | 67.91 | 68.12 |
| 1749 | #8 | 32 | 2015 | Male | 67.47 | 63.34 |
| 1750 | #8 | 36 | 2014 | Female | 66.62 | 67.59 |
| 1751 | #8 | 74 | 2014 | Female | 66.78 | 72.18 |
| 1752 | #8 | 74 | 2014 | Male | 70.59 | 70.99 |
| 1753 | #8 | 6 | 2014 | Female | 65.34 | 49.53 |
| 1754 | #8 | 74 | 2014 | Female | 63.43 | 65.68 |
| 1755 | #8 | 58 | 2014 | Male | 66.35 | 67.19 |
| 1756 | #8 | 47 | 2015 | Male | 68.03 | 66.59 |
| 1757 | #8 | 58 | 2014 | Male | 68.69 | 64.84 |
| 1758 | #8 | 37 | 2014 | Female | 64.57 | 66.65 |
| 1759 | #8 | 74 | 2015 | Female | 69.32 | 66.00 |
| 1760 | #8 | 11 | 2015 | Male | 66.39 | 60.28 |
| 1761 | #8 | 38 | 2015 | Male | 70.00 | 64.24 |
| 1762 | #19 | 59 | 2015 | Female | 67.19 | 72.32 |
| 1763 | #19 | 59 | 2015 | Female | 67.29 | 72.98 |
| 1764 | #19 | 12 | 2015 | Female | 65.94 | 62.81 |
| 1765 | #19 | 21 | 2015 | Female | 66.02 | 62.56 |
| 1766 | #19 | 25 | 2015 | Female | 66.12 | 68.96 |
| 1767 | #19 | 36 | 2015 | Female | 67.81 | 62.04 |
| 1768 | #19 | 54 | 2015 | Male | 66.15 | 71.96 |
| 1769 | #19 | 54 | 2015 | Female | 65.87 | 70.38 |
| 1770 | #19 | 28 | 2015 | Female | 64.51 | 66.35 |
| 1771 | #19 | 73 | 2015 | Male | 68.43 | 68.08 |
| 1772 | #19 | 38 | 2015 | Female | 66.40 | 70.71 |
| 1773 | #19 | 52 | 2015 | Male | 70.05 | 77.16 |
| 1774 | #19 | 17 | 2015 | Female | 66.39 | 65.55 |
| 1775 | #19 | 67 | 2014 | Female | 66.54 | 62.89 |
| 1776 | #19 | 68 | 2014 | Female | 65.21 | 64.34 |
| 1777 | #19 | 1 | 2015 | Male | 66.12 | 58.90 |
| 1778 | #19 | 6 | 2014 | Female | 67.32 | 55.33 |
| 1779 | #19 | 61 | 2014 | Female | 66.09 | 69.56 |
| 1780 | #19 | 59 | 2015 | Female | 68.53 | 75.86 |
| 1781 | #19 | 63 | 2015 | Female | 68.16 | 71.69 |
| 1782 | #19 | 34 | 2014 | Male | 67.01 | 66.68 |
| 1783 | #19 | 47 | 2014 | Female | 66.71 | 74.05 |
| 1784 | #19 | 58 | 2014 | Male | 67.65 | 71.11 |
| 1785 | #19 | 56 | 2015 | Male | 68.41 | 73.90 |
| 1786 | #19 | 40 | 2014 | Male | 68.09 | 64.95 |
| 1787 | #19 | 40 | 2014 | Male | 67.41 | 65.35 |
| 1788 | #19 | 26 | 2014 | Female | 65.91 | 58.22 |
| 1789 | #19 | 26 | 2014 | Male | 66.32 | 57.11 |
| 1790 | #19 | 47 | 2014 | Female | 66.43 | 62.33 |
| 1791 | #19 | 33 | 2014 | Female | 64.87 | 67.56 |
| 1792 | #19 | 28 | 2015 | Male | 63.51 | 56.83 |
| 1793 | #19 | 55 | 2014 | Female | 67.88 | 62.85 |
| 1794 | #19 | 11 | 2015 | Male | 66.97 | 64.74 |
| 1795 | #19 | 57 | 2014 | Male | 66.76 | 71.51 |
| 1796 | #19 | 44 | 2014 | Male | 66.88 | 66.20 |
| 1797 | #19 | 18 | 2014 | Female | 66.38 | 51.16 |
| 1798 | #19 | 32 | 2015 | Female | 69.81 | 70.57 |
| 1799 | #19 | 59 | 2015 | Female | 68.34 | 73.71 |
| 1800 | #19 | 40 | 2015 | Female | 68.94 | 74.08 |
| 1801 | #19 | 53 | 2014 | Female | 66.13 | 71.53 |
| 1802 | #19 | 54 | 2014 | Male | 67.54 | 70.48 |
| 1803 | #19 | 15 | 2014 | Male | 65.33 | 60.48 |
| 1804 | #19 | 71 | 2014 | Female | 64.51 | 64.25 |
| 1805 | #19 | 57 | 2014 | Male | 66.23 | 77.77 |
| 1806 | #19 | 71 | 2014 | Female | 65.87 | 65.30 |
| 1807 | #19 | 22 | 2014 | Female | 67.54 | 61.11 |
| 1808 | #19 | 71 | 2014 | Female | 65.98 | 69.46 |
| 1809 | #19 | 23 | 2014 | Female | 66.21 | 63.06 |
| 1810 | #19 | 47 | 2014 | Female | 66.94 | 75.09 |
| 1811 | #19 | 44 | 2014 | Male | 65.32 | 63.40 |
| 1812 | #19 | 41 | 2014 | Male | 66.13 | 61.88 |
| 1813 | #19 | 72 | 2014 | Male | 67.89 | 62.26 |
| 1814 | #19 | 6 | 2014 | Male | 66.54 | 49.21 |
| 1815 | #19 | 44 | 2014 | Female | 66.43 | 62.37 |
| 1816 | #19 | 44 | 2014 | Female | 66.31 | 61.71 |
| 1817 | #19 | 54 | 2015 | Male | 69.94 | 77.03 |
| 1818 | #19 | 43 | 2015 | Female | 66.94 | 68.18 |
| 1819 | #19 | 68 | 2014 | Male | 66.55 | 66.54 |
| 1820 | #19 | 72 | 2014 | Female | 66.09 | 72.56 |
| 1821 | #19 | 74 | 2014 | Female | 67.19 | 61.86 |
| 1822 | #19 | 69 | 2014 | Female | 67.56 | 69.13 |
| 1823 | #19 | 69 | 2014 | Male | 68.36 | 73.40 |
| 1824 | #19 | 27 | 2014 | Male | 67.29 | 67.75 |
| 1825 | #19 | 33 | 2014 | Male | 66.74 | 63.51 |
| 1826 | #5 | 17 | 2015 | Female | 75.08 | 57.59 |
| 1827 | #5 | 11 | 2015 | Male | 69.17 | 51.66 |
| 1828 | #5 | 13 | 2015 | Male | 64.19 | 56.25 |
| 1829 | #5 | 60 | 2015 | Female | 67.86 | 67.21 |
| 1830 | #5 | 50 | 2015 | Female | 66.44 | 62.71 |
| 1831 | #22 | 22 | 2015 | Male | 68.14 | 59.99 |
| 1832 | #22 | 22 | 2015 | Male | NA | NA |
| 1833 | #22 | 21 | 2015 | Female | 68.86 | 61.03 |
| 1834 | #22 | 59 | 2015 | Male | 67.95 | 63.02 |
| 1835 | #22 | 59 | 2015 | Female | 65.24 | 69.15 |
| 1836 | #22 | 40 | 2015 | Female | NA | NA |
| 1837 | #22 | 70 | 2015 | Female | 67.67 | 67.45 |
| 1838 | #22 | 67 | 2015 | Female | 70.30 | 72.03 |
| 1839 | #22 | 68 | 2015 | Male | 66.12 | 68.83 |
| 1840 | #22 | 68 | 2015 | Male | 69.71 | 69.36 |
| 1841 | #22 | 60 | 2015 | Male | 62.62 | 68.38 |
| 1842 | #22 | 68 | 2015 | Female | 72.32 | 70.32 |
| 1843 | #22 | 66 | 2015 | Female | 64.14 | 63.38 |
| 1844 | #22 | 60 | 2015 | Male | 64.05 | 62.25 |
| 1845 | #18 | 12 | 2015 | Male | 64.31 | 59.63 |
| 1846 | #18 | 60 | 2015 | Female | 67.20 | 68.89 |
| 1847 | #18 | 5 | 2015 | Female | NA | NA |
| 1848 | #18 | 68 | 2015 | Male | 66.96 | 69.21 |
| 1849 | #18 | 66 | 2015 | Female | 65.28 | 69.96 |
| 1850 | #18 | 66 | 2015 | Male | 65.85 | 71.10 |
| 1851 | #2 | 24 | 2015 | Female | 66.31 | 64.89 |
| 1852 | #2 | 22 | 2015 | Male | 71.43 | 62.28 |
| 1853 | #2 | 29 | 2015 | Female | 72.63 | 62.38 |
| 1854 | #2 | 5 | 2015 | Female | 64.01 | 42.85 |
| 1855 | #2 | 70 | 2015 | Female | 67.99 | 72.26 |
| 1856 | #2 | 24 | 2015 | Male | 65.61 | 58.64 |
| 1857 | #2 | 73 | 2015 | Male | 69.99 | 69.58 |
| 1858 | #2 | 7 | 2015 | Male | 71.60 | 55.69 |
| 1859 | #2 | 66 | 2015 | Male | 67.86 | 72.43 |
| 1860 | #2 | 50 | 2015 | Male | 65.63 | 66.90 |
| 1861 | #2 | 5 | 2015 | Female | 68.14 | 50.75 |
| 1862 | #2 | 59 | 2015 | Male | 66.30 | 66.33 |
| 1863 | #2 | 59 | 2015 | Male | 68.35 | 68.57 |
| 1864 | #2 | 70 | 2015 | Male | 69.66 | 65.90 |
| 1865 | #2 | 70 | 2015 | Female | 67.95 | 70.00 |
| 1866 | #2 | 73 | 2015 | Male | 70.81 | 68.04 |
| 1867 | #2 | 45 | 2015 | Female | 68.46 | 64.52 |
| 1868 | #17 | 14 | 2015 | Male | 63.12 | 58.75 |
| 1869 | #17 | 68 | 2015 | Male | 65.26 | 63.51 |
| 1870 | #17 | 42 | 2015 | Female | 66.39 | 63.69 |
| 1871 | #17 | 19 | 2015 | Female | 65.31 | 61.84 |
| 1872 | #17 | 57 | 2015 | Female | 67.14 | 63.14 |
| 1873 | #3 | 5 | 2015 | Male | 66.48 | 54.11 |
| 1874 | #3 | 66 | 2015 | Male | 68.79 | 67.10 |
| 1875 | #3 | 56 | 2015 | Male | 69.76 | 71.25 |
| 1876 | #3 | 54 | 2015 | Male | 72.34 | 69.14 |
| 1877 | #3 | 56 | 2015 | Female | 68.43 | 70.51 |
| 1878 | #3 | 17 | 2015 | Female | 66.98 | 55.60 |
| 1879 | #3 | 39 | 2015 | Female | 66.35 | 62.53 |
| 1880 | #3 | 38 | 2015 | Female | 68.87 | 68.32 |
| 1881 | #3 | 74 | 2015 | Female | 68.06 | 66.80 |
| 1882 | #3 | 68 | 2015 | Female | 70.94 | 68.02 |
| 1883 | #3 | 38 | 2015 | Female | 69.03 | 67.88 |
| 1884 | #3 | 4 | 2015 | Male | 71.64 | 55.51 |
| 1885 | #3 | 63 | 2015 | Female | 69.51 | 66.59 |
| 1886 | #3 | 50 | 2015 | Female | 67.87 | 63.57 |
| 1887 | #12 | 24 | 2015 | Male | 69.65 | 65.14 |
| 1888 | #12 | 24 | 2015 | Male | 65.33 | 58.88 |
| 1889 | #12 | 10 | 2015 | Female | 64.88 | 41.82 |
| 1890 | #12 | 73 | 2015 | Male | 67.54 | 72.83 |
| 1891 | #12 | 21 | 2015 | Male | 63.27 | 57.41 |
| 1892 | #12 | 54 | 2015 | Male | 65.62 | 64.68 |
| 1893 | #12 | 29 | 2015 | Female | 69.43 | 55.68 |
| 1894 | #12 | 22 | 2015 | Female | 65.49 | 60.83 |
| 1895 | #12 | 71 | 2015 | Female | 66.55 | 72.62 |
| 1896 | #12 | 39 | 2015 | Female | 68.90 | 67.27 |
| 1897 | #12 | 10 | 2015 | Female | 64.92 | 42.84 |
| 1898 | #20 | 74 | 2015 | Male | 66.67 | 65.09 |
| 1899 | #20 | 54 | 2015 | Female | 69.55 | 67.67 |
| 1900 | #20 | 24 | 2015 | Female | 66.77 | 66.44 |
| 1901 | #20 | 45 | 2015 | Male | 68.59 | 65.98 |
| 1902 | #20 | 31 | 2015 | Female | 70.92 | 64.69 |
| 1903 | #20 | 73 | 2015 | Male | 67.91 | 57.13 |
| 1904 | #20 | 17 | 2015 | Female | 72.37 | 57.93 |
| 1905 | #9 | 40 | 2015 | Male | 71.96 | 70.17 |
| 1906 | #9 | 24 | 2015 | Male | 68.35 | 62.42 |
| 1907 | #9 | 73 | 2015 | Male | 69.12 | 73.32 |
| 1908 | #9 | 74 | 2015 | Female | 66.57 | 68.66 |
| 1909 | #9 | 70 | 2015 | Female | 67.92 | 68.64 |
| 1910 | #9 | 63 | 2015 | Male | 66.98 | 69.04 |
| 1911 | #9 | 45 | 2015 | Male | 64.23 | 65.43 |
| 1912 | #9 | 49 | 2015 | Male | 63.29 | 66.98 |
| 1913 | #9 | 71 | 2015 | Male | 65.39 | 67.82 |
| 1914 | #9 | 1 | 2015 | Male | 66.63 | 57.03 |
| 1915 | #21 | 57 | 2015 | Female | 65.22 | 64.80 |
| 1916 | #21 | 25 | 2015 | Female | 63.84 | 69.31 |
| 1917 | #21 | 60 | 2015 | Female | 64.07 | 62.47 |
| 1918 | #21 | 59 | 2015 | Female | 65.14 | 63.00 |
| 1919 | #21 | 42 | 2015 | Male | 63.12 | 62.26 |
| 1920 | #21 | 49 | 2015 | Female | 65.89 | 66.38 |
| 1921 | #21 | 50 | 2015 | Female | 68.01 | 70.60 |
| 1922 | #21 | 66 | 2015 | Male | 68.72 | 68.47 |
| 1923 | #21 | 70 | 2015 | Male | 67.14 | 63.14 |
| 1924 | #21 | 74 | 2015 | Female | 63.29 | 63.24 |
| 1925 | #21 | 24 | 2015 | Male | 64.18 | 59.84 |
| 1926 | #21 | 59 | 2015 | Female | 68.01 | 69.40 |
| 1927 | #21 | 73 | 2015 | Female | 62.15 | 59.24 |
| 1928 | #15 | 73 | 2015 | Male | NA | NA |
| 1929 | #15 | 70 | 2015 | Female | 68.01 | 66.86 |
| 1930 | #15 | 71 | 2015 | Female | NA | NA |
| 1931 | #15 | 73 | 2015 | Female | 69.86 | 70.16 |
| 1932 | #15 | 38 | 2015 | Female | 64.23 | 56.15 |
| 1933 | #15 | 11 | 2015 | Female | 64.15 | 51.88 |
| 1934 | #15 | 67 | 2015 | Female | 68.72 | 70.25 |
| 1935 | #15 | 73 | 2015 | Male | 66.89 | 65.62 |
| 1936 | #15 | 32 | 2015 | Female | 68.97 | 62.70 |
| 1937 | #15 | 11 | 2015 | Male | 66.13 | 59.56 |
| 1938 | #15 | 60 | 2015 | Female | 66.39 | 63.39 |
| 1939 | #15 | 61 | 2015 | Female | 66.31 | 63.97 |
| 1940 | #15 | 36 | 2015 | Male | 70.15 | 68.14 |
| 1941 | #15 | 43 | 2015 | Male | 66.95 | 62.33 |
| 1942 | #15 | 74 | 2015 | Male | 68.79 | 68.70 |
| 1943 | #15 | 10 | 2015 | Male | 67.49 | 58.47 |
| 1944 | #15 | 59 | 2015 | Female | 70.51 | 70.58 |
| 1945 | #15 | 19 | 2015 | Female | 66.82 | 57.93 |
| 1946 | #15 | 71 | 2015 | Male | 69.13 | 76.13 |
| 1947 | #15 | 14 | 2015 | Female | 65.47 | 54.82 |
| 1948 | #15 | 38 | 2015 | Female | 66.15 | 65.95 |
| 1949 | #10 | 71 | 2015 | Male | 61.23 | 66.81 |
| 1950 | #10 | 46 | 2015 | Female | 67.14 | 67.70 |
| 1951 | #10 | 60 | 2015 | Male | 65.43 | 68.76 |
| 1952 | #10 | 71 | 2015 | Female | 66.23 | 69.01 |
| 1953 | #10 | 71 | 2015 | Female | 63.59 | 66.69 |
| 1954 | #10 | 42 | 2015 | Female | 68.17 | 68.31 |
| 1955 | #10 | 68 | 2015 | Male | 68.71 | 68.43 |
| 1956 | #10 | 46 | 2015 | Female | 66.23 | 64.02 |
| 1957 | #10 | 50 | 2015 | Female | 65.15 | 64.13 |
| 1958 | #10 | 66 | 2015 | Male | 64.53 | 69.62 |
| 1959 | #10 | 45 | 2015 | Female | 67.13 | 66.63 |
| 1960 | #10 | 63 | 2015 | Female | 65.12 | 71.30 |
| 1961 | #10 | 66 | 2015 | Female | 68.79 | 67.68 |
| 1962 | #14 | 71 | 2015 | Female | 68.17 | 68.80 |
| 1963 | #14 | 70 | 2015 | Male | 68.23 | 67.68 |
| 1964 | #14 | 54 | 2015 | Female | 67.18 | 67.08 |
| 1965 | #14 | 59 | 2015 | Female | 68.12 | 68.75 |
| 1966 | #14 | 66 | 2015 | Male | 67.12 | 69.92 |
| 1967 | #14 | 73 | 2015 | Female | 66.38 | 67.74 |
| 1968 | #14 | 4 | 2015 | Female | 67.84 | 52.30 |
| 1969 | #14 | 59 | 2015 | Female | 68.17 | 67.25 |
| 1970 | #14 | 25 | 2015 | Female | 67.89 | 62.40 |
| 1971 | #14 | 46 | 2015 | Male | 67.41 | 64.85 |
| 1972 | #14 | 24 | 2014 | Male | 66.55 | 60.96 |
| 1973 | #14 | 46 | 2015 | Female | 68.15 | 67.82 |
| 1974 | #14 | 66 | 2015 | Male | 65.13 | 64.70 |
| 1975 | #14 | 39 | 2015 | Female | 68.72 | 65.10 |
| 1976 | #14 | 46 | 2015 | Female | 66.37 | 65.14 |
| 1977 | #14 | 40 | 2015 | Female | 64.13 | 63.04 |
| 1978 | #14 | 53 | 2015 | Female | 69.31 | 69.18 |
| 1979 | #14 | 38 | 2015 | Female | 65.34 | 69.48 |
| 1980 | #14 | 57 | 2015 | Female | 68.17 | 70.80 |
| 1981 | #14 | 60 | 2015 | Male | 66.11 | 64.68 |
| 1982 | #14 | 39 | 2015 | Male | 65.31 | 65.25 |
| 1983 | #14 | 8 | 2015 | Male | 65.29 | 59.83 |
| 1984 | #14 | 5 | 2015 | Male | 68.79 | 61.92 |
| 1985 | #14 | 63 | 2015 | Male | 69.41 | 69.30 |
| 1986 | #14 | 28 | 2015 | Male | 67.91 | 64.84 |
| 1987 | #14 | 33 | 2015 | Female | 63.15 | 66.90 |
| 1988 | #14 | 10 | 2015 | Female | 66.31 | 52.96 |
| 1989 | #14 | 70 | 2015 | Male | 69.73 | 69.41 |
| 1990 | #14 | 5 | 2015 | Female | 65.49 | 50.62 |
| 1991 | #14 | 68 | 2015 | Male | 68.13 | 70.58 |
| 1992 | #14 | 74 | 2015 | Male | 68.15 | 71.01 |
| 1993 | #14 | 15 | 2015 | Female | 66.38 | 58.23 |
| 1994 | #14 | 19 | 2015 | Female | 62.11 | 57.30 |
| 1995 | #14 | 3 | 2015 | Female | 63.21 | 48.65 |
| 1996 | #14 | 53 | 2015 | Male | 66.89 | 63.39 |
| 1997 | #14 | 40 | 2015 | Female | 67.46 | 61.06 |
| 1998 | #14 | 60 | 2015 | Female | 67.89 | 72.29 |
| 1999 | #14 | 38 | 2015 | Male | 66.97 | 60.28 |
| 2000 | #14 | 45 | 2015 | Male | 68.99 | 69.15 |
| 2001 | #14 | 11 | 2015 | Female | 69.01 | 59.96 |
| 2002 | #14 | 73 | 2015 | Female | 66.03 | 63.11 |
| 2003 | #14 | 11 | 2015 | Female | 63.24 | 53.55 |
| 2004 | #14 | 17 | 2015 | Male | 63.54 | 56.63 |
| 2005 | #14 | 52 | 2015 | Male | 67.14 | 68.48 |
| 2006 | #14 | 25 | 2015 | Female | NA | NA |
| 2007 | #14 | 63 | 2015 | Female | 66.39 | 70.77 |
| 2008 | #14 | 35 | 2015 | Female | 65.34 | 65.95 |
| 2009 | #14 | 35 | 2015 | Female | 68.79 | 68.92 |
| 2010 | #14 | 38 | 2015 | Female | 67.34 | 64.22 |
| 2011 | #14 | 59 | 2015 | Male | 68.41 | 68.11 |
| 2012 | #14 | 57 | 2015 | Male | 69.75 | 69.89 |
| 2013 | #14 | 67 | 2015 | Male | 68.01 | 69.81 |
| 2014 | #14 | 22 | 2015 | Male | 63.59 | 60.28 |
| 2015 | #14 | 46 | 2015 | Male | 67.42 | 66.84 |
| 2016 | #14 | 50 | 2015 | Female | 65.34 | 64.87 |
| 2017 | #14 | 73 | 2015 | Female | 66.91 | 68.10 |
| 2018 | #14 | 67 | 2015 | Male | 68.79 | 71.37 |
| 2019 | #14 | 66 | 2015 | Male | 69.86 | 71.43 |
| 2020 | #14 | 5 | 2015 | Male | 63.11 | 57.17 |
| 2021 | #14 | 49 | 2015 | Male | 66.31 | 68.53 |
| 2022 | #16 | 68 | 2015 | Male | 68.01 | 69.26 |
| 2023 | #16 | 73 | 2015 | Female | 67.35 | 67.16 |
| 2024 | #16 | 15 | 2015 | Female | 64.13 | 53.46 |
| 2025 | #16 | 10 | 2015 | Male | 66.01 | 56.11 |
| 2026 | #16 | 10 | 2015 | Female | 67.90 | 50.84 |
| 2027 | #16 | 71 | 2015 | Female | 67.35 | 68.85 |
| 2028 | #16 | 50 | 2015 | Male | 66.81 | 66.20 |
| 2029 | #16 | 68 | 2015 | Female | 68.18 | 69.32 |
| 2030 | #16 | 68 | 2015 | Male | 67.10 | 70.38 |
| 2031 | #16 | 49 | 2015 | Male | 69.94 | 71.73 |
| 2032 | #16 | 46 | 2015 | Female | 64.97 | 66.02 |
| 2033 | #16 | 52 | 2015 | Male | 67.15 | 72.72 |
| 2034 | #16 | 71 | 2015 | Female | 68.74 | 67.53 |
| 2035 | #16 | 52 | 2015 | Female | 65.26 | 71.29 |
| 2036 | #16 | 60 | 2015 | Female | 69.89 | 73.75 |
| 2037 | #16 | 64 | 2015 | Female | 70.19 | 75.79 |
| 2038 | #16 | 73 | 2015 | Female | 69.24 | 66.60 |
| 2039 | #16 | 24 | 2015 | Male | 67.51 | 63.99 |
| 2040 | #16 | 64 | 2015 | Male | 68.17 | 67.18 |
| 2041 | #16 | 60 | 2015 | Male | 65.64 | 67.21 |
| 2042 | #16 | 66 | 2015 | Female | 66.54 | 66.98 |
| 2043 | #16 | 60 | 2015 | Female | 67.24 | 62.60 |
| 2044 | #16 | 18 | 2015 | Female | 65.95 | 50.42 |
| 2045 | #16 | 59 | 2015 | Female | 68.75 | 73.92 |
| 2046 | #16 | 45 | 2015 | Female | 65.91 | 61.65 |
| 2047 | #16 | 56 | 2015 | Female | 69.55 | 68.63 |
| 2048 | #16 | 45 | 2015 | Female | 73.36 | 71.88 |
| 2049 | #16 | 26 | 2015 | Female | 64.41 | 54.25 |
| 2050 | #16 | 71 | 2015 | Female | 67.45 | 62.35 |
| 2051 | #16 | 17 | 2015 | Female | 67.59 | 54.55 |
| 2052 | #16 | 66 | 2015 | Male | 67.29 | 66.61 |
| 2053 | #16 | 66 | 2015 | Female | 65.88 | 67.38 |
| 2054 | #16 | 66 | 2015 | Female | 65.69 | 69.63 |
| 2055 | #16 | 45 | 2015 | Female | 65.93 | 66.98 |
| 2056 | #16 | 32 | 2015 | Male | 66.91 | 65.74 |
| 2057 | #16 | 45 | 2015 | Male | 69.72 | 68.01 |
| 2058 | #16 | 52 | 2015 | Male | 64.08 | 66.54 |
| 2059 | #16 | 45 | 2015 | Female | 63.25 | 67.76 |
| 2060 | #16 | 63 | 2015 | Male | 67.90 | 68.28 |
| 2061 | #16 | 45 | 2015 | Female | 65.25 | 71.62 |
| 2062 | #16 | 45 | 2015 | Male | 68.58 | 67.31 |
| 2063 | #16 | 60 | 2015 | Female | 66.22 | 69.06 |
| 2064 | #16 | 66 | 2015 | Female | 68.60 | 70.88 |
| 2065 | #16 | 70 | 2015 | Female | 70.04 | 72.28 |
| 2066 | #16 | 50 | 2015 | Female | 65.30 | 62.69 |
| 2067 | #16 | 63 | 2015 | Male | 69.53 | 70.09 |
| 2068 | #16 | 71 | 2015 | Female | 65.73 | 72.43 |
| 2069 | #16 | 35 | 2015 | Female | 67.46 | 67.70 |
| 2070 | #16 | 74 | 2015 | Female | 61.08 | 60.36 |
| 2071 | #16 | 70 | 2015 | Female | 63.33 | 67.22 |
| 2072 | #16 | 74 | 2015 | Male | 70.02 | 74.06 |
| 2073 | #16 | 74 | 2015 | Male | 65.82 | 65.67 |
| 2074 | #1 | 71 | 2015 | Male | 68.37 | 68.04 |
| 2075 | #1 | 66 | 2015 | Female | 68.85 | 69.08 |
| 2076 | #11 | 43 | 2015 | Female | 63.69 | 64.42 |
| 2077 | #11 | 66 | 2015 | Male | 72.20 | 69.43 |
| 2078 | #11 | 53 | 2015 | Female | 68.31 | 73.87 |
| 2079 | #11 | 46 | 2015 | Male | 67.74 | 69.39 |
| 2080 | #11 | 14 | 2015 | Male | 62.37 | 65.60 |
| 2081 | #11 | 24 | 2015 | Female | 64.27 | 67.84 |
| 2082 | #11 | 39 | 2015 | Male | 69.79 | 69.41 |
| 2083 | #11 | 74 | 2015 | Female | 68.81 | 71.38 |
| 2084 | #11 | 15 | 2015 | Female | 67.50 | 56.40 |
| 2085 | #11 | 11 | 2015 | Male | 69.77 | 63.61 |
| 2086 | #11 | 1 | 2015 | Female | 65.66 | 43.68 |
| 2087 | #11 | 4 | 2015 | Male | 71.82 | 54.34 |
| 2088 | #11 | 4 | 2015 | Female | 62.94 | 41.55 |
| 2089 | #11 | 67 | 2015 | Male | 70.22 | 70.33 |
| 2090 | #11 | 68 | 2015 | Male | 68.99 | 68.53 |
| 2091 | #11 | 43 | 2015 | Female | 64.58 | 62.49 |
| 2092 | #11 | 74 | 2015 | Female | 70.67 | 64.38 |
| 2093 | #11 | 60 | 2015 | Female | 71.45 | 71.25 |
| 2094 | #11 | 49 | 2015 | Male | 68.53 | 71.77 |
| 2095 | #11 | 46 | 2015 | Male | 68.86 | 71.99 |
| 2096 | #11 | 19 | 2015 | Female | 69.43 | 58.88 |
| 2097 | #11 | 31 | 2015 | Male | 68.49 | 60.11 |
| 2098 | #11 | 36 | 2015 | Female | 68.33 | 62.08 |
| 2099 | #11 | 10 | 2015 | Female | 66.93 | 46.97 |
| 2100 | #11 | 14 | 2015 | Male | 65.64 | 56.35 |
| 2101 | #11 | 52 | 2015 | Male | 68.48 | 64.94 |
| 2102 | #11 | 47 | 2015 | Female | 65.24 | 66.60 |
| 2103 | #11 | 71 | 2015 | Male | 70.32 | 69.41 |
| 2104 | #11 | 52 | 2015 | Female | 70.24 | 64.22 |
| 2105 | #11 | 24 | 2015 | Male | 70.60 | 61.17 |
| 2106 | #11 | 25 | 2015 | Male | 69.06 | 61.76 |
| 2107 | #11 | 32 | 2015 | Male | 67.62 | 63.56 |
| 2108 | #11 | 60 | 2015 | Male | 70.52 | 67.11 |
| 2109 | #11 | 31 | 2015 | Male | 65.91 | 61.04 |
| 2110 | #11 | 60 | 2015 | Female | 71.46 | 75.59 |
| 2111 | #11 | 67 | 2015 | Female | 66.99 | 68.23 |
| 2112 | #11 | 32 | 2015 | Female | 71.16 | 67.85 |
| 2113 | #8 | 28 | 2015 | Male | 69.02 | 62.63 |
| 2114 | #8 | 63 | 2015 | Male | 70.02 | 70.32 |
| 2115 | #8 | 53 | 2015 | Male | 67.49 | 69.38 |
| 2116 | #8 | 53 | 2015 | Male | 68.88 | 68.99 |
| 2117 | #8 | 53 | 2015 | Male | 66.92 | 67.42 |
| 2118 | #8 | 54 | 2015 | Male | 71.61 | 72.05 |
| 2119 | #8 | 35 | 2015 | Female | 68.68 | 62.10 |
| 2120 | #8 | 29 | 2015 | Male | 67.89 | 62.13 |
| 2121 | #8 | 42 | 2015 | Male | 70.64 | 72.74 |
| 2122 | #8 | 73 | 2015 | Male | 69.51 | 72.77 |
| 2123 | #8 | 17 | 2015 | Male | 70.18 | 61.96 |
| 2124 | #8 | 49 | 2015 | Male | 68.79 | 67.20 |
| 2125 | #8 | 67 | 2015 | Male | 70.14 | 68.35 |
| 2126 | #8 | 70 | 2015 | Female | 64.20 | 66.58 |
| 2127 | #8 | 15 | 2015 | Female | 63.96 | 54.45 |
| 2128 | #8 | 25 | 2015 | Female | 66.45 | 58.83 |
| 2129 | #8 | 74 | 2015 | Female | 68.28 | 65.66 |
| 2130 | #8 | 73 | 2015 | Female | 67.80 | 72.09 |
| 2131 | #8 | 67 | 2015 | Female | 71.06 | 68.22 |
| 2132 | #8 | 50 | 2015 | Female | 69.82 | 70.72 |
| 2133 | #8 | 66 | 2015 | Female | 70.25 | 73.34 |
| 2134 | #8 | 66 | 2015 | Male | 71.03 | 73.88 |
| 2135 | #8 | 29 | 2015 | Male | 70.15 | 68.14 |
| 2136 | #19 | 18 | 2015 | Female | 70.05 | 66.84 |
| 2137 | #19 | 18 | 2015 | Female | 66.98 | 62.78 |
| 2138 | #19 | 17 | 2015 | Male | 66.12 | 64.79 |
| 2139 | #19 | 50 | 2015 | Male | 69.15 | 75.08 |
| 2140 | #19 | 66 | 2015 | Male | 70.96 | 78.06 |
| 2141 | #19 | 60 | 2015 | Female | 67.14 | 68.07 |
| 2142 | #19 | 43 | 2015 | Male | 65.17 | 69.03 |
| 2143 | #19 | 18 | 2015 | Female | 66.30 | 61.85 |
| 2144 | #19 | 73 | 2015 | Female | 71.11 | 78.26 |
| 2145 | #19 | 67 | 2015 | Male | 70.53 | 75.16 |
| 2146 | #19 | 25 | 2015 | Male | 65.13 | 60.15 |
| 2147 | #19 | 46 | 2015 | Female | 64.19 | 62.24 |
| 2148 | #19 | 15 | 2015 | Female | 64.12 | 62.29 |
| 2149 | #19 | 68 | 2015 | Male | 68.97 | 74.04 |
| 2150 | #19 | 28 | 2015 | Male | 62.98 | 52.86 |
| 2151 | #19 | 24 | 2015 | Female | 66.37 | 70.94 |
| 2152 | #19 | 11 | 2015 | Female | 66.87 | 58.96 |
| 2153 | #19 | 73 | 2015 | Female | 70.28 | 76.07 |
| 2154 | #19 | 42 | 2015 | Male | 69.77 | 76.35 |
| 2155 | #19 | 52 | 2015 | Male | 67.41 | 69.37 |
| 2156 | #19 | 56 | 2015 | Male | 68.74 | 76.28 |
| 2157 | #19 | 45 | 2015 | Female | 66.13 | 72.93 |
| 2158 | #6 | 38 | 2015 | Male | 66.43 | 62.01 |
| 2159 | #14 | 70 | 2015 | Male | 65.12 | 70.72 |
| 2160 | #14 | 53 | 2015 | Male | 69.86 | 69.87 |
| 2161 | #14 | 66 | 2015 | Female | 68.17 | 69.25 |
