# Supplementary material for: Ecological conditions experienced by offspring during pregnancy and early post-natal life determine mandible size in roe deer
Source: PLoS One. 2019 Sep 11;14(9):e0222150. doi: 10.1371/journal.pone.0222150 (PMC6738612; doi:10.1371/journal.pone.0222150)
Supplement: S4 Table — Variables associated to roe deer mandible growth recorded in the 2013–2015 period in each hunting zone of Arezzo province (Tuscany, Central Italy). Pregnancy corresponds to post-diapause period. (DOCX) [file pone.0222150.s006.docx]

**Ecological conditions experienced by offspring during pregnancy and early post-natal life determine mandible growth in roe deer.**

PLoS ONE

Anna Maria De Marinis, Roberta Chirichella^*^, Elisa Bottero, Marco Apollonio

** Department of Veterinary Medicine, University of Sassari, via Vienna 2, I-07100 Sassari, Italy;* [*rchirichella@uniss.it*](mailto:rchirichella@uniss.it)

**S4 Table. List and description of independent variables.** Variables associated to roe deer mandible growth recorded in the 2013-2015 period in each hunting zone of Arezzo province (Tuscany, Central Italy). Pregnancy corresponds to post-diapause period.

| **Independent variable** | **Description** | **Period of data collection** | **Roe deer life cycle** | | **Data availability** |
| --- | --- | --- | --- | --- | --- |
|  |  |  |  |  |  |
|  |  |  | **Adult female (F)** | **Offspring (O)** |  |
| JDATE | The number of elapsed days since the January 1^st^ | January-March | - | - | Fish and Wildlife Service  of the Arezzo province |
| SEX | Juvenile sex | January-March | - | - | Fish and Wildlife Service  of the Arezzo province |
| JBM | Juvenile eviscerated body mass | January-March | - | - | Fish and Wildlife Service  of the Arezzo province |
| FBM | Mean dressed body mass of adult females (≥2 years) culled in each hunting zone per hunting season | January-March | - | - | Fish and Wildlife Service  of the Arezzo province |
| Autumn_T (F) | Daily average temperature (°C) | September-November | Post-rut |  | 2 weather stations of the National Forestry Service (UTB, 2015) and 8 weather stations of the Regional Agency for Agricultural Development and Innovation in Tuscany (ARSIA, 2015) |
| Winter_T (F) | Daily average temperature (°C) | December-February | Pregnancy |  |  |
| Spring_T (F) | Daily average temperature (°C) | March-May | Final part of pregnancy and birth period |  |  |
| Summer_T (F,O) | Daily average temperature (°C) | June-August |  | Lactation |  |
| Autumn_T (O) | Daily average temperature (°C) | September-November |  | Post-lactation |  |
| Autumn_P (F) | Cumulated daily precipitation (mm) | September-November | Post-rut |  |  |
| Winter_P (F) | Cumulated daily precipitation (mm) | December-February | Pregnancy |  |  |
| Spring_P (F) | Cumulated daily precipitation (mm) | March-May | Final part of pregnancy and birth period |  |  |
| Summer_P (F,O) | Cumulated daily precipitation (mm) | June-August |  | Lactation |  |
| Autumn_P (O) | Cumulated daily precipitation (mm) | September-November |  | Post-lactation |  |
| MEAN SNOW COVER | Average of maximum percentual snow cover extent derived from a remote index of snow cover presence (binary variable, 8 day intervals at 500 m resolution) | December-March | Pregnancy |  | MOD10A2 - https://search.earthdata.nasa.gov/ |
| DD SNOW COVER | Number of days in which maximum percentual snow cover extent derived from a remote index of snow cover presence (binary variable, 8 day intervals at 500 m resolution) was ≥ 40% | December-March | Pregnancy |  |  |
| fPAR_TOTAL | Total sum of values of Fraction of Photosynthetically Active Radiation absorbed by vegetation (fPAR; dimensionless; 1,000 m spatial resolution; 8 days temporal granularity) | March-October  year t-1 and year t | Pre-rut, rut, and post-rut period | Birth period, lactation, and post-lactation | MOD15A2 (Terra platform) - http://reverb.echo.nasa.gov/ |
| fPAR_MEAN | Mean value of Fraction of Photosynthetically Active Radiation absorbed by vegetation (fPAR; dimensionless; 1,000 m spatial resolution; 8 days temporal granularity) | March-October  year t-1 and year t | Pre-rut, rut, and post-rut period | Birth period, lactation, and post-lactation |  |
| AGRICULTURAL LAND | Percentage of agricultural land (Corine Land Cover class = 2) | - | - | - | Corine Land Cover map (2012) - http://land.copernicus.eu/pan-european/corine-land-cover/clc-2012 |
| DENSITY | Number of roe deer/100 ha | May-June | - | - | Fish and Wildlife Service  of the Arezzo province |
