# Supplementary material for: Ecological conditions experienced by offspring during pregnancy and early post-natal life determine mandible size in roe deer
Source: PLoS One. 2019 Sep 11;14(9):e0222150. doi: 10.1371/journal.pone.0222150 (PMC6738612; doi:10.1371/journal.pone.0222150)
Supplement: S5 Table — Hunting area, number, extent and percentage of areas monitored by drive censuses in each hunting district of the Arezzo province (Tuscany, Central Italy) in May and June. See S1 Fig for the location of the network of permanent sample areas. (DOCX) [file pone.0222150.s007.docx]

**Ecological conditions experienced by offspring during pregnancy and early post-natal life determine mandible size in roe deer.**

PLoS ONE

Anna Maria De Marinis, Roberta Chirichella^*^, Elisa Bottero, Marco Apollonio

** Department of Veterinary Medicine, University of Sassari, via Vienna 2, I-07100 Sassari, Italy;* [*rchirichella@uniss.it*](mailto:rchirichella@uniss.it)

**S5 Table. Network of permanent monitoring sample areas for roe deer.** Hunting area, number, extent and percentage of areas monitored by drive censuses in each hunting district of the Arezzo province (Tuscany, Central Italy) in May and June. See S1 Fig. for the location of the network of permanent sample areas.

| **Hunting district**  **code** | **Hunting area**  **(km^2^)** | **Number of**  **monitoring plots** | **Monitoring area**  **(km^2^)** | **Monitoring area**  **(%)** |
| --- | --- | --- | --- | --- |
| # 1 | 103.25 | 16 | 7.04 | 6.82 |
| # 2 | 132.58 | 9 | 3.89 | 2.93 |
| # 3 | 76.25 | 8 | 3.40 | 4.46 |
| # 4 | 46.70 | 4 | 1.70 | 3.64 |
| # 5 | 105.10 | 7 | 3.08 | 2.93 |
| # 6 | 92.27 | 11 | 4.75 | 5.15 |
| # 7 | 63.81 | 4 | 1.70 | 2.66 |
| # 8 | 90.76 | 10 | 4.42 | 4.87 |
| # 9 | 80.44 | 10 | 4.07 | 5.06 |
| # 10 | 212.89 | 12 | 5.28 | 2.48 |
| # 11 | 106.73 | 7 | 3.04 | 2.85 |
| # 12 | 101.05 | 8 | 3.52 | 3.48 |
| # 13 | 84.96 | 7 | 3.06 | 3.60 |
| # 14 | 113.44 | 8 | 3.50 | 3.09 |
| # 15 | 101.96 | 8 | 3.49 | 3.42 |
| # 16 | 84.84 | 8 | 3.52 | 4.15 |
| # 17 | 69.92 | 7 | 3.07 | 4.39 |
| # 18 | 99.20 | 7 | 3.08 | 3.10 |
| # 19 | 83.74 | 14 | 6.01 | 7.18 |
| # 20 | 86.93 | 8 | 3.52 | 4.05 |
| # 21 | 61.35 | 7 | 3.00 | 4.89 |
| # 22 | 90.47 | 7 | 3.02 | 3.34 |
| **Total** | **2088.63** | **187** | **81.16** | **3.89** |
