# Supplementary material for: Ecological conditions experienced by offspring during pregnancy and early post-natal life determine mandible size in roe deer
Source: PLoS One. 2019 Sep 11;14(9):e0222150. doi: 10.1371/journal.pone.0222150 (PMC6738612; doi:10.1371/journal.pone.0222150)
Supplement: S6 Table — Pearson correlation coefficient (rp) among environmental and climatic variables associated to roe deer mandible growth recorded in the 2013–2015 period in each hunting ground of Arezzo province (Tuscany, Central Italy). See S4 Table for the description of each variable. (DOCX) [file pone.0222150.s008.docx]

**Ecological conditions experienced by offspring during pregnancy and early post-natal life determine mandible size in roe deer.**

PLoS ONE

Anna Maria De Marinis, Roberta Chirichella^*^, Elisa Bottero, Marco Apollonio

** Department of Veterinary Medicine, University of Sassari, via Vienna 2, I-07100 Sassari, Italy;* [*rchirichella@uniss.it*](mailto:rchirichella@uniss.it)

**S6 Table. Correlation matrix for selected independent variables.** Pearson correlation coefficient (*r_p_*) among environmental and climatic variables associated to roe deer mandible growth recorded in the 2013-2015 period in each hunting ground of Arezzo province (Tuscany, Central Italy). See S4 Table for the description of each variable.

|  | **Autumn_T_(F)_** | **Winter_T_(F)_** | **Spring_T_(F)_** | **Summer_T_(F,O)_** | **Autumn_T _(O)_** | **Autumn_P_(F)_** | **Winter_P _(F)_** | **Spring_P_(F)_** | **Summer_P_(F,O)_** | **Autumn_P_(O)_** | **M SNOW COVER** | **DD SNOW COVER *** | **fPAR_MEAN** | **fPAR_TOTAL **** | **AGRIC LAND** |
| --- | --- | --- | --- | --- | --- | --- | --- | --- | --- | --- | --- | --- | --- | --- | --- |
| **Autumn_T_(F)_** |  | 0.53 | 0.45 | 0.33 | 0.47 | 0.01 | -0.11 | -0.31 | -0.08 | 0.03 | -0.3 | -0.3 | -0.03 | -0.03 | 0.04 |
| **Winter_T _(F)_** |  |  | 0.48 | 0.39 | 0.28 | 0.2 | 0.07 | -0.21 | 0.07 | -0.42 | -0.25 | -0.25 | 0.38 | 0.38 | 0.12 |
| **Spring_T_(F)_** |  |  |  | 0.48 | 0.31 | -0.04 | -0.16 | -0.4 | -0.09 | -0.07 | -0.16 | -0.16 | 0.41 | 0.41 | 0.07 |
| **Summer_T_(F,O)_** |  |  |  |  | 0.38 | -0.4 | -0.12 | -0.22 | -0.43 | 0.28 | -0.45 | -0.45 | 0.27 | 0.27 | 0.15 |
| **Autumn_T_(O)_** |  |  |  |  |  | 0.09 | -0.05 | -0.35 | -0.05 | -0.03 | -0.31 | -0.31 | -0.11 | -0.1 | 0.05 |
| **Autumn_P_(F)_** |  |  |  |  |  |  | 0.3 | 0.08 | 0.18 | -0.04 | -0.07 | -0.07 | -0.09 | -0.09 | 0.01 |
| **Winter_P_(F)_** |  |  |  |  |  |  |  | 0.5 | 0.3 | 0.15 | 0.11 | 0.11 | -0.01 | -0.01 | -0.03 |
| **Spring_P_(F)_** |  |  |  |  |  |  |  |  | -0.31 | 0.45 | -0.19 | -0.19 | 0.44 | 0.44 | 0.01 |
| **Summer_P_(F,O)_** |  |  |  |  |  |  |  |  |  | 0.11 | -0.04 | -0.04 | 0.33 | 0.33 | -0.01 |
| **Autumn_P_(O)_** |  |  |  |  |  |  |  |  |  |  | 0.08 | 0.08 | 0.05 | 0.05 | 0.02 |
| **M SNOW COVER** |  |  |  |  |  |  |  |  |  |  |  | **0.99** | 0.08 | 0.08 | -0.21 |
| **DD SNOW COVER** |  |  |  |  |  |  |  |  |  |  |  |  | 0.08 | 0.08 | -0.21 |
| **fPAR_MEAN** |  |  |  |  |  |  |  |  |  |  |  |  |  | **0.99** | -0.11 |
| **fPAR_TOTAL** |  |  |  |  |  |  |  |  |  |  |  |  |  |  | -0.11 |
| **AGRIC LAND** |  |  |  |  |  |  |  |  |  |  |  |  |  |  |  |

* = The number of days in which maximum percentual snow cover extent was ≥ 40% (DD SNOW COVER) was excluded from model selection according to its collinearity with the average of maximum percentual snow cover extent (M SNOW COVER)

** = The total sum of values of Fraction of Photosynthetically Active Radiation absorbed by vegetation (fPAR_TOTAL) was excluded from model selection according to its collinearity with the mean value of Fraction of Photosynthetically Active Radiation absorbed by vegetation (fPAR_MEAN)
