# Supplementary material for: Ecological conditions experienced by offspring during pregnancy and early post-natal life determine mandible size in roe deer
Source: PLoS One. 2019 Sep 11;14(9):e0222150. doi: 10.1371/journal.pone.0222150 (PMC6738612; doi:10.1371/journal.pone.0222150)
Supplement: S7 Table — Top model set containing models explaining the variation in the length of the anterior (A) and posterior (B) sections of the mandible for juvenile roe deer (8–10 months) hunted within the Arezzo Province from January to March over three consecutive hunting seasons (2013–2015) with a ΔAIC that is ≤2. The best model in bold. [AIC = Akaike Information Criterion; 𝚫AIC = AIC (model)–AIC (best model); Wt = AIC weights]. See S4 Table for the description of independent variables. (DOCX) [file pone.0222150.s009.docx]

**Ecological conditions experienced by offspring during pregnancy and early post-natal life determine mandible size in roe deer.**

PLoS ONE

Anna Maria De Marinis, Roberta Chirichella^*^, Elisa Bottero, Marco Apollonio

** Department of Veterinary Medicine, University of Sassari, via Vienna 2, I-07100 Sassari, Italy;* [*rchirichella@uniss.it*](mailto:rchirichella@uniss.it)

**S7 Table.** Set of most parsimonious models. Top model set containing models explaining the variation in the length of the anterior (A) and posterior (B) sections of the mandible for juvenile roe deer (8-10 months) hunted within the Arezzo Province from January to March over three consecutive hunting seasons (2013-2015) with a ΔAIC that is ≤2. The best model in bold. [AIC = Akaike Information Criterion; 𝚫AIC = AIC (model) – AIC (best model); Wt = AIC weights]. See S4 Table for the description of independent variables.

**A)**

| **Component models**  **(Dependent variable: Anterior section)** | **AIC** | **𝚫AIC** | **Wt** |
| --- | --- | --- | --- |
| **[1] Sex + JBM + FBM + Winter_T + Summer_T + fPAR_MEAN + Agric Land + Density** | **9142.20** | **0.00** | **0.47** |
| **[2]** J Date + Sex + JBM + FBM + Winter_T + Summer_T + Spring_P + fPAR_MEAN + Agric Land + Density | 9142.98 | 0.79 | 0.32 |
| **[3]** J Date + Sex + JBM + FBM + Winter_T + Summer_T + Spring_P + fPAR_MEAN + Agric Land + Density | 9143.89 | 1.69 | 0.20 |

**B)**

| **Component models**  **(Dependent variable: Posterior section)** | **AIC** | **𝚫AIC** | **Wt** |
| --- | --- | --- | --- |
| **[1] J Date + Sex + JBM + Summer_T + Autumn_P + fPAR_MEAN + Agric Land + Density + Sex:Summer_T** | **11099.16** | **0.00** | **0.38** |
| **[2]** J Date + Sex + JBM + Summer_T + Autumn_T + Autumn_P + fPAR_MEAN + Agric Land + Density + Sex:Summer_T | 11099.88 | 0.72 | 0.26 |
| **[3]** J Date + Sex + JBM + Summer_T + Autumn_P + fPAR_MEAN + Agric Land + Density + Sex:Summer_T + Sex:Agric Land | 11100.27 | 1.11 | 0.22 |
| **[4]** J Date + Sex + JBM + Summer_T + Autumn_T + Autumn_P + fPAR_MEAN + Agric Land + Density + Sex:Summer_T + Sex:Agric Land | 11101.09 | 1.93 | 0.14 |
